# Accurate and efficient machine learning interatomic potentials for finite temperature modelling of molecular crystals†

**DOI:** 10.1039/d5sc01325a

**Published:** 2025-05-23

**Authors:** Flaviano Della Pia, Benjamin X. Shi, Venkat Kapil, Andrea Zen, Dario Alfè, Angelos Michaelides

**Affiliations:** a Yusuf Hamied Department of Chemistry, University of Cambridge Cambridge CB2 1EW UK am452@cam.ac.uk v.kapil@ucl.ac.uk; b Department of Physics and Astronomy, University College London London UK; c Thomas Young Centre and London Centre for Nanotechnology, University College London London WC1E 6BT UK; d Dipartimento di Fisica Ettore Pancini, Università di Napoli Federico II Monte S. Angelo I-80126 Napoli Italy; e Department of Earth Sciences, University College London London WC1E 6BT UK

## Abstract

As with many parts of the natural sciences, machine learning interatomic potentials (MLIPs) are revolutionizing the modelling of molecular crystals. However, challenges remain for the accurate and efficient calculation of sublimation enthalpies – a key thermodynamic quantity measuring the stability of a molecular crystal. Specifically, two key stumbling blocks are: (i) the need for thousands of *ab initio* quality reference structures to generate training data; and (ii) the sometimes unreliable nature of density functional theory, the main technique for generating such data. Exploiting recent developments in foundation models for chemistry and materials science alongside accurate quantum diffusion Monte Carlo benchmarks, offers a promising path forward. Herein, we demonstrate the generation of MLIPs capable of describing molecular crystals at finite temperature and pressure with sub-chemical accuracy, using as few as ∼200 data structures; an order of magnitude improvement over the current state-of-the-art. We apply this framework to compute the sublimation enthalpies of the X23 dataset, accounting for anharmonicity and nuclear quantum effects, achieving sub-chemical accuracy with respect to experiment. Importantly, we show that our framework can be generalized to crystals of pharmaceutical relevance, including paracetamol and aspirin. Nuclear quantum effects are also accurately captured as shown for the case of squaric acid. By enabling accurate modelling at ambient conditions, this work paves the way for deeper insights into pharmaceutical and biological systems.

## Abstract 2

We fine-tune machine learning interatomic potentials to accurately model molecular crystals at finite temperature with the inclusion of nuclear quantum effects.

## Graphical abstract

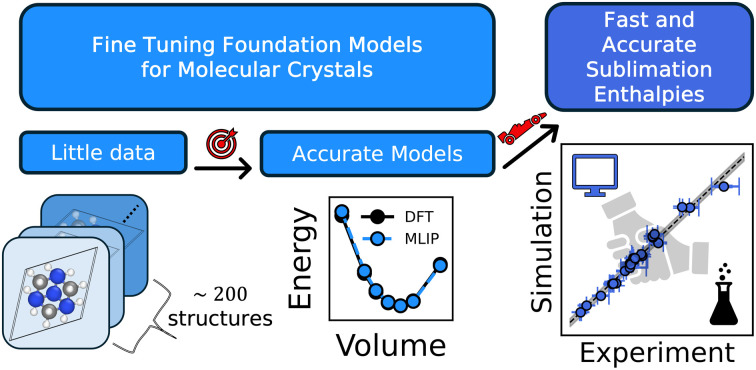

## 1. Introduction

Research and development in molecular crystals drives innovation across several impactful fields, from organic semiconductors^1,2^ and optoelectronics^3^ to life-saving pharmaceuticals.^4,5^ In pharmaceuticals, the structures of molecular crystals dictate not just the stability of compounds, but also how effectively a drug can be absorbed, its efficacy, and even its safety. Computational approaches have become essential for aiding experimental structure determination.^6–10^ Accurate predictions are especially important for sublimation processes, as the sublimation enthalpy of pharmaceutical compounds affects stability and drug solubility, which in turn influences therapeutic dosage, toxicity, and bioavailability.^11–15^

Unfortunately, the routine modelling of molecular crystals is constrained by a cost-accuracy trade-off. Classical force fields are a commonly adopted approach for modelling the potential energy surface (PES) of molecular crystals, offering computational efficiency and enabling the estimation of sublimation enthalpies under ambient conditions. Substantial advancements have been made using empirical descriptions of intermolecular interactions.^6,16–18^ However, their reliance on empirical parametrization sometimes compromises accuracy, undermining predictive reliability.^6,18,19^ Significant progress has been achieved in modelling the PES of molecular crystals using electronic structure theory approaches.^9,10,16,20–34^ Density Functional Theory (DFT) represents the first step up the accuracy-cost ladder beyond empirical force fields. However, the higher cost of DFT force evaluations typically implies approximations for the vibrational contributions, such as the harmonic or quasi-harmonic approximation (QHA). Even within the QHA framework, computational costs scale significantly with system size, requiring up to 3N force calculations for N atoms in the simulation cell (for a system with no symmetry). In addition, the QHA inherently lacks a full description of anharmonicity and nuclear quantum effects (NQEs), which can be critical for molecular crystals, especially in pharmaceutical applications.^19,35^ While anharmonicity can be incorporated *via* finite-temperature classical molecular dynamics (MD), and both effects are captured by path integral MD (PIMD), these methods are computationally prohibitive. Hundreds of thousands of force evaluations are generally needed, making them impractical for large systems. In addition, even DFT approximations often fall short in accuracy, struggling to capture the complex intermolecular interactions that characterize molecular crystals. Such interactions, particularly in systems with competing polymorphs where small energy differences dictate stability, often require the accuracy of expensive beyond DFT methods.^27,29–31,36^

Machine learning interatomic potentials (MLIPs) represent a promising alternative, aiming to combine the accuracy of *ab initio* approaches with the efficiency of less computationally intensive force evaluations. MLIPs, either trained with periodic unit cells or molecular cluster approaches, have provided a significant leap towards the calculation of accurate thermodynamic stabilities of molecular crystals,^10,35,37–45^ although often previous calculations have been restricted to zero temperature lattice energies. Nonetheless, the widespread application of MLIPs was still constrained by notable limitations. Training an MLIP typically necessitates costly *ab initio* MD (AIMD) simulations to generate the required training datasets. In addition, even models trained on thousands of structures may yield training errors comparable to chemical accuracy (conventionally ∼4 kJ mol^−1^), potentially undermining the reliability of their predictions for the relative stabilities of molecular crystals. However, recent algorithmic improvements have transformed the landscape of MLIP development.^46–48^ Improvement in data efficiency and reductions in training errors have facilitated the creation of foundation models for chemistry and materials science.^46,48–55^ These models provide qualitative – and in many cases, quantitative – accuracy across a substantial portion of the periodic table, and they have the promising potential to be fine-tuned to high accuracy for specific applications with minimal additional data.^56^

In this work, we exploit the training performance of the MACE MLIP architecture^47,57^ to deliver data-efficient MLIPs that achieve sub-chemical accuracy for molecular crystals with respect to the underlying DFT PES with as few as ∼200 data points, an approximately order of magnitude data efficiency improvement compared to previous work.^35^ In detail, we fine-tune the MACE-MP-0b3 (ref. 57) foundation model for each molecular crystal in the X23 dataset. X23 is a diverse dataset of 23 molecular crystals characterised by a delicate interplay of intermolecular interactions including hydrogen bonding and dispersion forces, for which a large number of experimental measurements of the sublimation enthalpy is available.^58–61^ On the other hand, accurate estimates of the sublimation enthalpies *via* computational approaches have been sought for decades.^24,62,63^ Our fine-tuned models achieve excellent accuracy on lattice energies, equation of state (EOS), and quasi-harmonic vibrational energies compared to the reference DFT functional (vdW-DF2), which was chosen based on a benchmark against DMC reference lattice energies^30^ reported in Appendix A. We apply the fine-tuned models to compute the vibrational contribution to the sublimation enthalpies of the X23 dataset with the inclusion of anharmonicity and NQEs, which is added to the reference DMC lattice energy to obtain the final sublimation enthalpies. The sublimation enthalpies computed in this work agree with available experimental estimates with an average error <4 kJ mol^−1^, and come at a cost within the recently suggested threshold for applicability of a computational method to be economically viable for routine screening of molecular crystals stabilities.^40^

In addition, we showcase the reliability and robustness of our framework by fine tuning MLIPs that achieve excellent accuracy (with respect to vdW-DF2) for systems of pharmaceutical interest such as paracetamol, aspirin, and squaric acid. This work highlights how state-of-the-art MLIPs facilitate the routine modelling of molecular crystals at finite temperatures and pressures with sub-chemical accuracy. We hope this work will contribute to achieving first-principles accuracy in the study of systems relevant to pharmaceuticals and biology.

## 2. Framework for data-efficient MLIPs with sub-chemical accuracy

We begin by describing the procedure used to fine-tune so-called foundation machine learning models to produce accurate MLIPs for molecular crystals. Our approach relies on foundation models for chemistry and materials science, *i.e.* MLIPs trained on large DFT datasets that qualitatively reproduce the underlying PES for a wide range of materials. Specifically, we use the MACE-MP-0b3 (ref. 57) model, pre-trained on MPtrj, a subset of optimised inorganic crystals from the Materials Project database.^64^ This model has been shown to have PBE-level accuracy for numerous systems, and serves as a useful starting point for improving the potential for a given problem with minimal data.

**Fig. 1:**
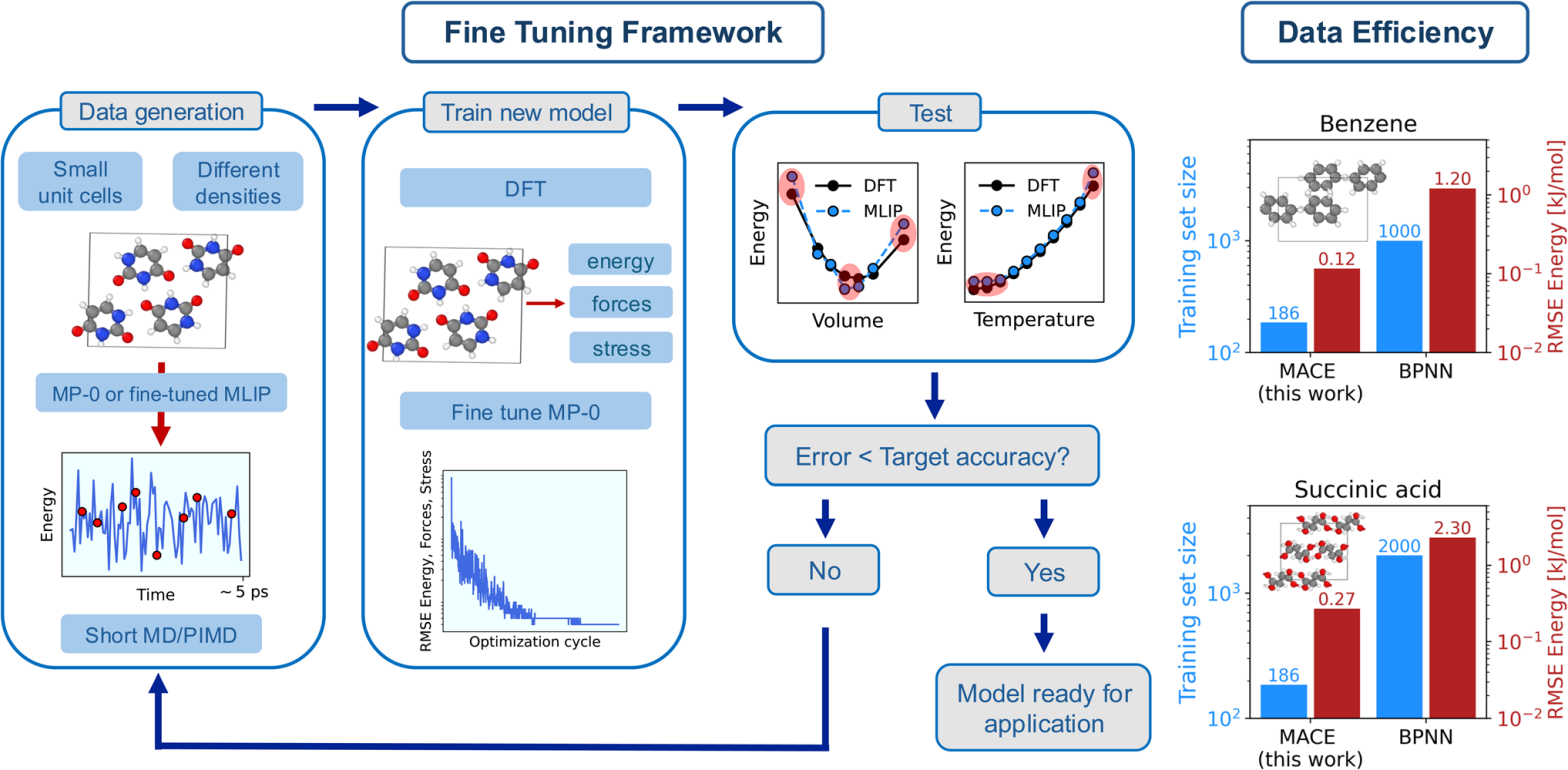
(left) Framework used in this work to fine-tune MACE-MP-0b3 to reproduce the potential energy surface of molecular crystals with sub-chemical accuracy. Each step of ‘Fine Tuning Framework’, *i.e.* ‘Data generation’, ‘Train new model’, and ‘Test’ is described in the text, with additional computational details reported in the Methods section and in Sec. S3 of the ESI.† (right) Data efficiency and energy errors of the fine-tuned models. The figure reports a comparison on the training set size (blue bars) and the root mean square error (RMSE) of the energy in the validation set (red bars) for benzene and succinic acid between this work and state-of-the-art Behler Parrinello Neural Network (BPNN) MLIPs.^35^

The main idea behind the current approach is summarised in Fig. 1, with each step of the fine tuning framework described in the following.

Our goal is to develop an accurate potential for NPT simulations to simulate molecular crystals at desired temperatures and pressures rigorously, hence a correct description of a system at different densities is required. Therefore, we first generate a minimal training set by sampling a molecular crystal phase space around the equilibrium volume at low temperatures (‘Data generation’ in Fig. 1). In particular, we run short MD simulations for different cells across the EOS, as described in Methods and in Sec. S2 of the (ref. 65) (ESI).† The key aspect here is that the MD simulations are directly run with the foundation model in the first iteration, and with the fine-tuned model in subsequent training iterations. This allows us to avoid the extremely costly step of producing data with AIMD. The initial training set is then generated by sampling (randomly) a few structures (∼10 per volume) from the MD trajectories. The MACE-MP-0b3 model is then fine-tuned by optimizing its parameters to minimize errors on energy, forces, and stress (‘Train new model’ in Fig. 1). Subsequently, we test the fine-tuned model (‘Test’ in Fig. 1). In particular, we test the models on the EOS (total electronic energy per molecule of the solid as a function of the volume) and its vibrational energy (total energy per molecule as a function of the temperature) in the quasi-harmonic approximation (QHA). The training set is then gradually augmented (with ∼5 structures per volume) until the tested properties are obtained with chemical (or sub-chemical) accuracy. Details of the models performance on the EOS and QHA vibrational properties are reported in Sec. S10 of the ESI.†

We further demonstrate the potential and applicability of the fine-tuned models for the simulation of molecular crystals at ambient temperature with the inclusion of anharmonicity and NQEs. For this reason, the training set of each molecular crystal has been additionally augmented with the inclusion of structures sampled at higher temperatures in PIMD simulations as well as structures for the gas phase. Additional computational details on each step of the fine tuning framework are reported in the Methods section. The breakdown of the cost of each step of the framework (including the calculations of reference DFT EOS, vibrational properties, and training data, as well as the cost of the fine tuning of each model) and the number of structures in the training set for each system is reported in Sec. S3 of the ESI.†

Finally, we discuss the data efficiency of the framework. In fact, we achieve a sub-chemical accuracy reproduction of the PES of molecular crystals by using training sets with an average of approximately ∼200 data points and a computational costs of ∼30 CPU node-hours. The computational cost (estimated on one Ice Lake node on the Cambridge Service for Data Driven Discovery (CSD3)^66^ with 76 cores and 256 GB of RAM) includes the calculations of the DFT energy, forces and stresses for the training set, and it does not include the calculation of the reference EOS and vibrational frequencies. As showcased in Fig. 1 for the cases of benzene and succinic acid (‘Data efficiency’), this represents about an order of magnitude improvement on data efficiency (*i.e.*, the amount of data needed to achieve the desired accuracy on the training errors) and energy training errors (see Table S27 of the ESI†) compared to Behler Parrinello Neural Network (BPNN) MLIPs for molecular crystals.^35^

## 3. Anharmonic sublimation enthalpies of molecular crystals with nuclear quantum effects

The fine tuning procedure described above delivers data efficient and accurate MLIPs for molecular crystals. The efficacy and accuracy of the fine-tuned models is now showcased by tackling a long standing challenge in the computational study of molecular crystals: a fast and accurate computation of fully anharmonic finite temperature thermodynamic stabilities. In particular, we consider the X23 dataset,^24,62,63^ the most widely used dataset for molecular crystals. A large number of experimental measurements of the sublimation enthalpies of molecules in the X23 dataset is available,^58–61^ and it was shown that for several systems the experimental uncertainty is larger than ∼4 kJ mol^−1^, and it can be as large as ∼20 kJ mol^−1^.^30^

The X23 sublimation enthalpies have been previously computed with DFT with the QHA^62,63^ and with the inclusion of thermal expansion.^24^ Hence, the accuracy of the available estimates can in principle be affected by both the accuracy of the electronic structure method (*e.g.*, the choice of the functional in the DFT calculations) and the statistical mechanics description of the nuclei (*i.e.*, lack of anharmonicity and NQEs). Here, we leverage recent reference DMC values of the X23 lattice energies^30^ to benchmark several DFT approximations and determine a functional that achieves chemical accuracy on the dataset (see Sec. S1 of the ESI†). Subsequently, we train 23 fine-tuned MACE models, one for each molecular crystal in X23. The fine-tuned models achieve sub-chemical accuracy errors compared to the reference functional (vdW-DF2) on the lattice energy, the EOS, and the quasi harmonic vibrational properties (see Sec S10 of the ESI†). We then use the fine-tuned models to compute the vibrational contribution to the sublimation enthalpies of X23 with three different approximations: (i) the QHA; (ii) the inclusion of anharmonicity with a classical description of the nuclei (referred to as MD); and (iii) the inclusion of anharmonicity with a quantum description of the nuclei (referred to as PIMD). The zero temperature electronic contribution to the sublimation enthalpies, *i.e.* the lattice energy, is finally corrected to the DMC accuracy as described in Methods. We note here that although the DFT functional was selected based on a lattice energy benchmark, the lattice energy typically represents the major contribution (∼80%) of the sublimation enthalpy. In addition, the choice of the functional (among “reliable” ones) plays a minor role in the determination of the vibrational contribution, as shown in Sec. S11 of the ESI.†

**Fig. 2:**
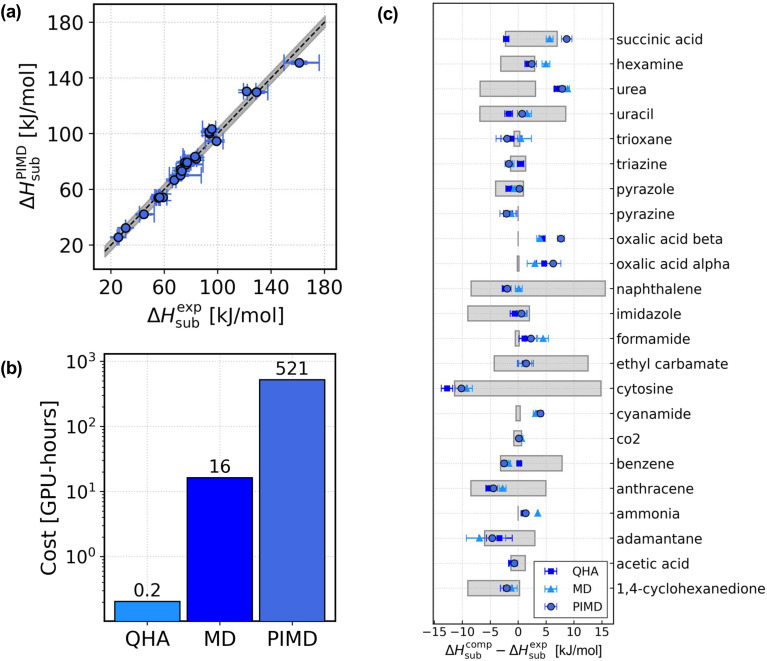
Sublimation enthalpies of the X23 dataset. (a) Scatter plot of the sublimation enthalpies of the X23 dataset computed with the PIMD approach against the median of the experimental value for each system.^58–61^ The horizontal error bars represent the experimental uncertainty and go from the minimum to the maximum measured value. (b) Estimated computational cost of the sublimation enthalpies for a single molecular crystal with the three different approaches used in this work, QHA, MD and PIMD. The cost is estimated for 1,4-cyclohexanedione with ∼200 atoms in the simulated supercell. The reported cost of the sublimation enthalpy calculations does not include the training of the model. (c) Comparison between experiments and the computational sublimation enthalpies obtained with the three different approaches considered in this manuscript. The figure shows, for each system in X23, the difference between the computational sublimation enthalpies Δ*H*^comp^_sub_ and the median of the experimental sublimation enthalpies Δ*H*^exp^_sub_, for the QHA (blue squares), the MD (light blue triangles) and the PIMD (navy circles). The grey shaded bars represent the uncertainty on the experimental estimates.

In Fig. 2, we report the analysis of the sublimation enthalpies of the X23 dataset. In Fig. 2(a), we show the scatter plot of the sublimation enthalpies computed with the PIMD approach against the median of the experimental values. The vertical error bars take into account the uncertainty on the DMC lattice energy and the statistical sampling error of the PIMD simulations, computed with reblocking. The horizontal bars represent the uncertainty on the experimental numbers and go from the minimum to the maximum experimental value. The grey shaded area represents an uncertainty of ∼4 kJ mol^−1^. The figure shows that the MLIPs trained in this work reproduce the experimental sublimation enthalpies with chemical accuracy. In Fig. 2(c) we report the sublimation enthalpies computed with the three different approaches for each system in X23 and compare with the median of the experimental values. The grey shaded bars represent the uncertainty on the experimental estimates, which often reflects the number of available measurements, as discussed in ref. 30. Importantly, when taking into account the large uncertainty on the experimental numbers as well as the error bars on the computational sublimation enthalpies, we find that the sublimation enthalpies of the X23 dataset are well reproduced also with the MD approach and even at the QHA level. Measuring the performance of the computational approaches as a mean absolute error (MAE) with respect to the median of the experimental data, we obtain MAE^QHA^ ∼ 2.7 ± 0.8 kJ mol^−1^, MAE^MD^ ∼ 3.0 ± 0.8 kJ mol^−1^, and MAE^PIMD^ ∼ 3.3 ± 0.9 kJ mol^−1^. On average, the sublimation enthalpies are predicted with chemical accuracy with all three approaches, with all three approaches equivalent within the error bars. Overall, the large uncertainties on the experimental sublimation enthalpies^30^ and the error bars on the computational estimates do not allow for a rigorous assessment of the three different approaches.

However, anharmonicity and NQEs are expected to play a greater role in larger and more flexible molecular crystals. Hence the importance of this work, which showcases the feasibility of finite temperature modelling of molecular crystals with NQEs.

While the data-efficiency of the approach has been discussed, in Fig. 2(b) now we discuss the computational cost. We report the approximate computational cost (in GPU-hours) of the calculation of the sublimation enthalpies with QHA, MD, and PIMD for a showcase system from X23: 1,4-cyclohexanedione. The reported cost does not include the cost of the fine tuning of the model nor the cost of the DMC lattice energy correction. It was recently suggested that the acceptable amount of CPU time required for a single free-energy calculation for a method to be economically feasible in screening molecular crystals structures was about 24 000 core-hours.^40^ The simulations in this work were performed on GPUs (single NVIDIA A100-SXM-80GB GPU on CSD3 (ref. 66)), therefore we evaluate the efficiency of our method in terms of the actual monetary cost and notably find that the cost of our simulations is approximately within the threshold even with the inclusion of NQEs (see ref. 67 for details of the cost evaluation).

**Fig. 3:**
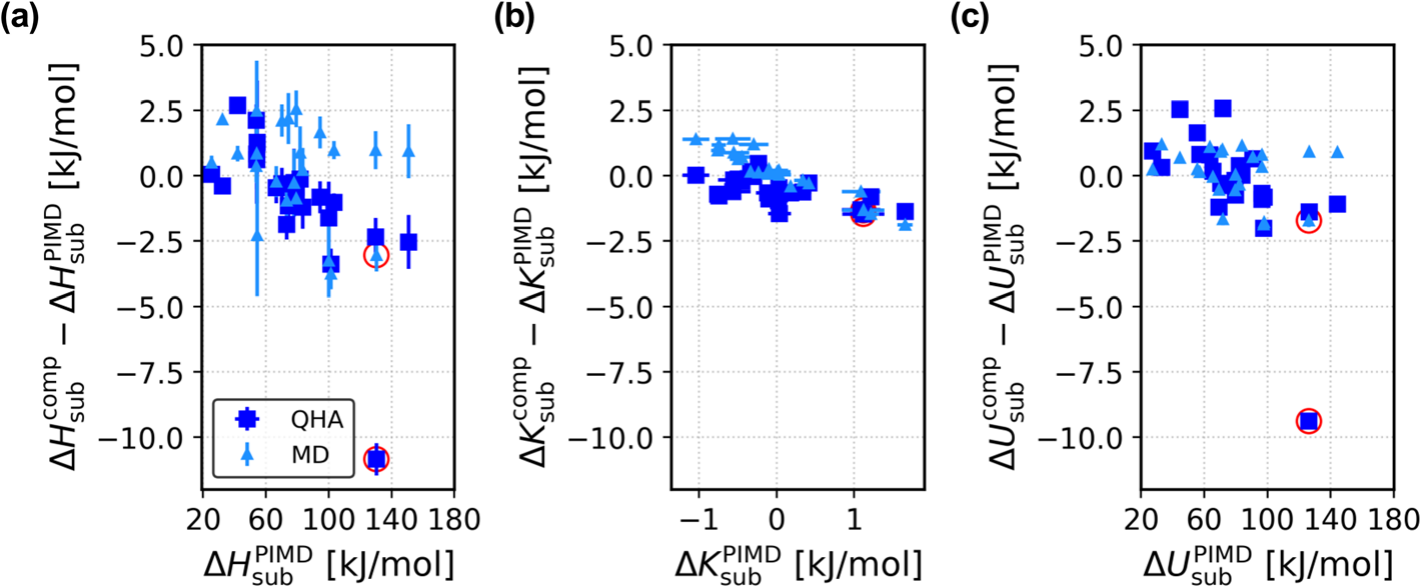
Importance of anharmonicity and NQEs for the X23 dataset. (a–c) Plot of the deviation of the sublimation enthalpies (a), the kinetic energy contribution (b), and the potential energy contribution (c) computed with QHA (blue squares) and MD (light blue triangles) against the PIMD values. The empty red circles highlight the data for succinic acid.

Now, we focus on a comparison among the sublimation enthalpies computed with the three different approaches. In Fig. 3(a) we report the scatter plot of the difference between Δ*H*^PIMD^_sub_ and the sublimation enthalpies computed with the QHA and MD approaches, against the PIMD values. Overall, we observe that the inclusion of NQEs can account for a ∼4 kJ mol^−1^ change in the sublimation enthalpy, which can be non negligible when computing energy differences with chemical accuracy. The system in X23 where anharmonicity plays a major role is succinic acid, which is highlighted with red circles. In Sec. S14 of the ESI,† we show that the torsion angle of the four carbon atoms in the gas phase oscillates between ∼75°, ∼180° and ∼290°. This anharmonic feature cannot be described with the harmonic approximation, where only small displacements of the atoms are allowed. Therefore, the contribution of anharmonicity and NQEs is larger and more significant for succinic acid, accounting for a ∼11 kJ mol^−1^ change in the sublimation enthalpy.

Similarly, in Fig. 3(b) and (c) we report the scatter plots of the kinetic energy *K* (b) and potential energy *U* (c) contributions to the sublimation enthalpy differences plotted in panel (a) (see the Methods section for a breakdown of each contribution to the sublimation enthalpy in each approximation). For the majority of the X23 systems where anharmonicity and NQEs play a minor role, we observe that a similar correction of ∼2 kJ mol^−1^ affects the kinetic and potential energy contribution to the sublimation enthalpy. For succinic acid, where anharmonicity plays a major role, the main correction is due to the potential energy contribution, with Δ*U*^QHA^_sub_ − Δ*U*^PIMD^_sub_ ∼ 10 kJ mol^−1^. This analysis suggests that the effect of anharmonicity and NQEs on the sublimation enthalpy of a highly anharmonic molecular crystal can be primarily estimated by the calculation of the potential energy contribution.

## 4. Extension of the framework to pharmaceutical crystals

The robust fine-tuning framework presented here is not limited to the X23 dataset. In fact, in this work we tested the validity of the framework for the description of systems of pharmaceutical interest, as well as a highly polymorphic and ubiquitous system like ice. The ice polymorphs application is presented in Sec. S12 of the ESI,† where we show that an MLIP fine-tuned on ∼464 structures correctly reproduces the zero temperature relative stability of 15 ice polymorphs, including two polymorphs not explicitly represented in the training set. Here in the main manuscript, we focus on the generalization of the framework's applicability to pharmaceutical systems of interest, namely paracetamol and aspirin. We also consider squaric acid, known for the highly quantum nature of its hydrogen bond.^68^ As shown in the Sec. S15,† the fine-tuned MLIPs correctly reproduce the reference DFT, with errors <0.5 kJ mol^−1^ for the lattice energy and <2 kJ mol^−1^ on the QHA vibrational energy.

**Fig. 4:**
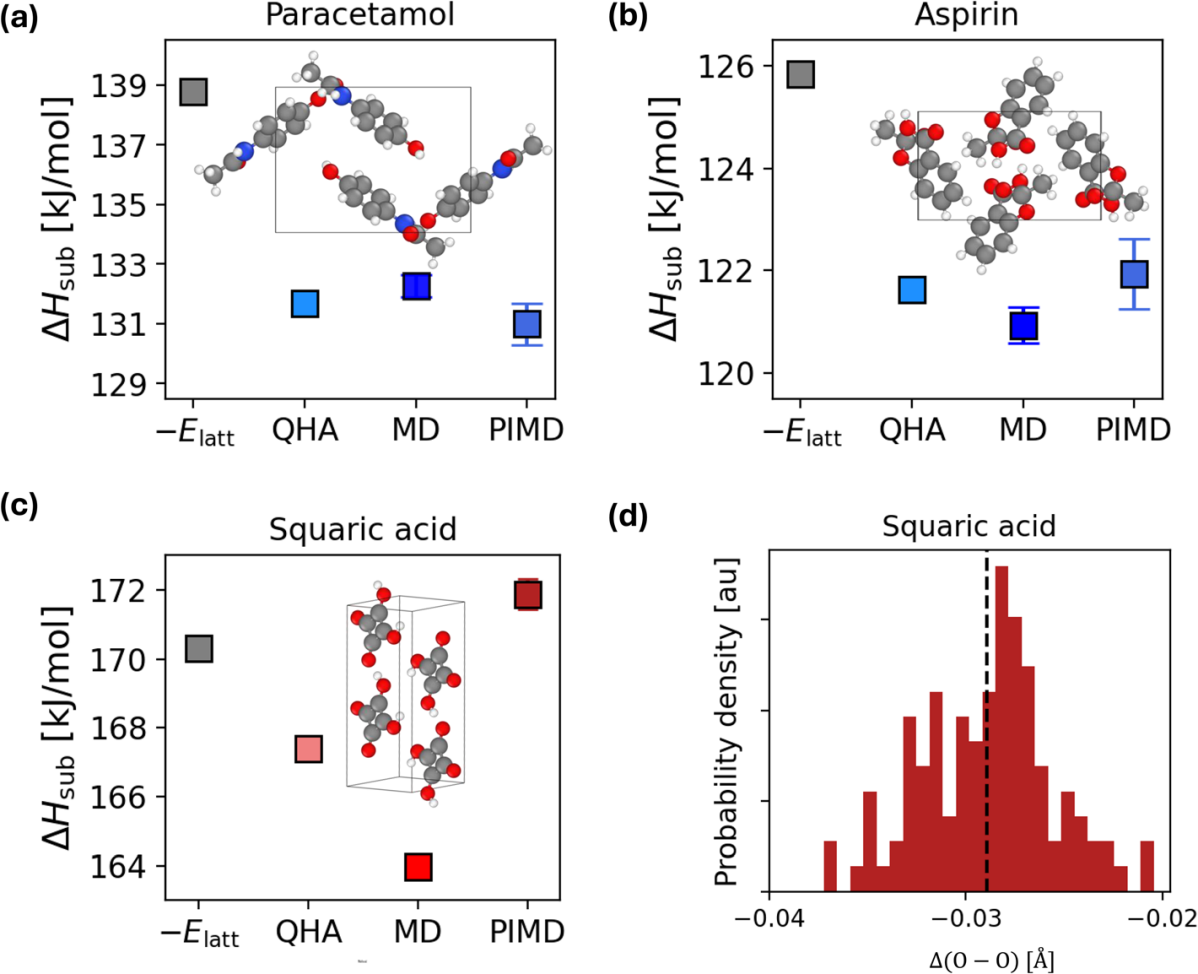
Generalization of the framework to systems of pharmaceutical interest. We report the sublimation enthalpies of (a) form I of paracetamol, (b) form I of aspirin, and (c) squaric acid. The experimental sublimation enthalpies of paracetamol and squaric acid are ∼117.9 ± 0.7 kJ mol^−1^ (ref. 61 and 69) at room temperature and ∼152 kJ mol^−1^ (ref. 61 and 70) at ∼486 K, respectively. Each plot shows the sublimation enthalpy Δ*H*_sub_ (in kJ mol^−1^) computed with the perfect lattice approximation (the negative of the lattice energy *E*_latt_), the QHA, MD and PIMD. In each panel we show the structure of the considered system, with oxygen atoms in red, hydrogen atoms in white, carbon atoms in grey and nitrogen atoms in blue. The 1 × 2 × 1 supercell is shown for squaric acid, to help visualize the in-plane hydrogen bonded molecules. (d) Conventional Ubbelodhe effect for squaric acid. The plot shows the distribution of the Δ(O–O) = (O–O)^H^ − (O–O)^D^ in room temperature PIMD simulations. The average of the distribution is reported with a black dashed line.

In Fig. 4(a–c), we report the room-temperature sublimation enthalpies of paracetamol (a), aspirin (b), and squaric acid (c) using four different approximations: the zero-temperature perfect lattice approximation (negative of the lattice energy *E*_latt_), QHA, MD, and PIMD.

We first address the importance of finite temperature contributions. These contributions have a noticeable impact on the sublimation enthalpy (variations of ∼4 kJ mol^−1^), underscoring the need to go beyond the perfect lattice approximation. Using fast and accurate MLIPs, the QHA contribution can be computed in as little as 0.2 GPU-hours.

For paracetamol and aspirin, as with most molecular crystals in the X23 dataset, anharmonicity and NQEs make minimal corrections to the QHA, with differences between Δ*H*^QHA^_sub_ and Δ*H*^MD/PIMD^_sub_ of <4 kJ mol^−1^. However, for squaric acid, the inclusion of anharmonicity and NQEs is more significant, with Δ*H*^PIMD^_sub_ − Δ*H*^QHA^_sub_ ∼ 4 kJ mol^−1^ and Δ*H*^PIMD^_sub_ − Δ*H*^MD^_sub_ ∼ 8 kJ mol^−1^.

We now comment on the accuracy of the sublimation enthalpy with respect to experiment, for which we found available estimates for paracetamol^61,69^ and squaric acid.^61,70^ As mentioned above, the fine-tuned MLIPs correctly reproduce the underlying DFT level of theory with sub-chemical accuracy errors (<2 kJ mol^−1^) on lattice energies and QHA vibrational energy. However, for these systems we find that the chosen DFT functional does not appear to perform well. Although experimental values of molecular crystals' sublimation enthalpies might have larger uncertainties than those reported in a single experiment,^30^ the sublimation enthalpies computed in this work differ by ∼15–20 kJ mol^−1^ from experiment. As described in Sec. S15.3,† the sublimation enthalpies of paracetamol, aspirin, and squaric acid do not contain the correction to the zero temperature contribution (−*E*_latt_) computed with DMC. Therefore, the larger errors between the computational and experimental sublimation enthalpies could be ascribed to the DFT functional used in our calculations (selected on a benchmark for the X23 dataset). Future work will be related to the extension of reference DMC calculations to the challenging systems described in this section.

Finally, we discuss the importance of NQEs. NQEs can influence the interaction strength and consequently the structure of H-bonded systems.^71,72^ In H-bonded crystals, this effect is known as the Ubbelohde effect, where replacing H with deuterium (D) causes a change of the O–O distance, and consequently of the ferroelectric phase-transition temperature.^73^ Squaric acid yields an elongation of its lattice constant and O–O distance upon deuteration, an effect known as conventional Ubbelohde effect (as opposed to the negative Ubbelohde effect, where O–O decreases upon deuteration).^68^ In Fig. 4(d) we show that the Ubbelodhe effect at room temperature is correctly described with our model. In fact, we report the change in the O–O distance between hydrogenated [(O–O^H^)] and deuterated [(O–O^D^)] squaric acid. In particular, we plot the distribution of the difference Δ(O–O) = (O–O)^H^ − (O–O)^D^ in the PIMD simulations. The mean elongation ∼0.03 Å correctly describes the conventional Ubbelodhe effect, and agrees with the previously reported value^68^ of ∼0.04 Å obtained with *ab initio* PIMD, but comes at a fraction of the computational cost.

## 5. Discussion and conclusion

In this work, we leverage recent developments on MLIPs and insight into molecular crystal lattice energies with DMC to study finite temperature stabilities of molecular crystals with sub-chemical accuracy by fine tuning a foundation model for chemistry and materials science. In particular, we fine-tune the MACE-MP-0b3 foundation model to obtain MLIPs that accurately reproduce lattice energies, equations of state, and quasi-harmonic thermodynamic properties of the X23 dataset. The procedure followed in this work builds on recent preliminary work, where some of us reported the data-efficient generation of an MLIP for three ice polymorphs.^56^ Importantly, in this work we consider organic molecular crystals that are not represented in the original training set of the pre-trained model.^57^ Moreover, the generation of the training set in this work was directly run with MACE-MP-0b3 rather than with AIMD, which significantly reduces the overall computational cost. The training sets contain as few as ∼200 data points and required ∼30 node-hours of DFT calculations (cost estimated on one Ice Lake node with 76 cores and 256 GB of RAM), which represents an almost order of magnitude improvement compared to the state-of-the-art. In summary, while fine tuning is known in general to be a powerful approach towards improving the accuracy of machine learning models, here we show that for molecular crystals unprecedented accuracy can be obtained with few data points.

The fine-tuned models are used to compute the vibrational contribution to the sublimation enthalpies of the X23 dataset with three different approximations: QHA, anharmonicity with a classic description of the nuclei, and anharmonicity with inclusion of NQEs. The sublimation enthalpies reported in this work agree with experiment with sub-chemical accuracy for all the considered systems, and notably come at a cost that is within the recently suggested threshold for the widespread applicability of a method to the calculation of finite temperature free energies for molecular crystals.^40^ In addition, we show that our framework can be applied to deliver MLIPs that efficiently reproduce the DFT PES for systems such as paracetamol, aspirin, and squaric acid. The results showcase that the strategy followed in this manuscript is robust, and provides a way to obtain MLIPs that achieve excellent accuracy with respect to the reference PES with low data and computational cost even for systems of pharmaceutical interest.

We now discuss the limitations of our work and potential improvements for the near future. Two main aspects merit consideration: (i) enhancing the framework from a technical and methodological standpoint; and (ii) expanding its range of applicability. The former involves two key technical aspects: the accuracy of the training data and a systematic understanding of how data requirements depend on the starting foundation model.

Regarding training data accuracy, as evidenced by the sublimation enthalpies of paracetamol and squaric acid, the choice of the DFT functional – critical for accurate lattice energy calculations – remains a significant challenge in modelling molecular crystals. Future work will focus on leveraging the low data requirements of our framework to directly learn the PESs obtained from explicitly correlated methods.^74–77^ In fact, some of us recently showcased how Random Phase Approximation accuracy could be reached for the prototypical case of hexagonal ice.^56^

With respect to the starting foundation model, foundation model development and fine-tuning strategies are rapidly evolving research areas. Interesting future directions involve: (i) testing our framework with recent MACE foundation models trained on larger datasets such as Alexandria^78^ and OMat,^79^ the pre-trained organic machine learning force field MACE-OFF23,^55^ or models with different architectures such as Orb,^80^ MatterSim,^81^ and more; and (ii) exploring new fine tuning strategies to possibly lower the data-requirement of our framework.^82,83^

We now turn to future potential applications of our framework, which include: extending our approach to larger and more complex drug-like compounds and analysing highly polymorphic organic molecular crystals. The ice polymorphs test reported in the ESI† serves as a stringent benchmark for polymorphic systems, as their energies lie within a range comparable to chemical accuracy. Interestingly, the fine-tuned model correctly predicts the energetics of polymorphs which are not included in the training set. However, the systems presented in this manuscript are still relatively limited in size, flexibility, and molecular complexity. In the future, it would be particularly interesting to test our approach on more challenging and highly polymorphic organic systems, *e.g.* compounds from the 7th blind test challenge,^16^ or to compute the phase diagram of larger pharmaceutical compounds.^40^ As mentioned above, for such systems, questions arise both regarding the accuracy of the underlying DFT PES as well as the data requirement necessary for its accurate description with MLIPs.

Finally, we discuss the relevance of our work to crystal structure prediction (CSP), an exciting field with recent developments both in the structure generation^16,84,85^ and polymorph ranking tasks.^16,42,86^ Here, we focused on developing a modular pipeline to compute the sublimation enthalpy associated with a single crystal structure. A different and exciting research direction for the application of MLIPs in the modelling of molecular crystals is the determination of the most stable polymorph among numerous candidates in CSP ranking tasks, *e.g.* the blind test challenges.^16,87–92^ CSP ranking is a multi-fidelity screening problem, typically requiring a hierarchy of models with increasing accuracy. In general, different models are used to first screen among thousands of generated structures to identify the most promising candidates, and then predict relative energies of a handful of final candidates. For the initial screening, one is often interested in shortlisting structures within a few kcal mol^−1^ of the lowest-energy structures. Here, one could desire a general and transferable model accurate for “all” molecular crystals. In Sec. S13 of the ESI† we report a comparison between the 23 different fine-tuned models and a single “global” model, trained on the 23 joined training sets, showing that comparable accuracy can be obtained with the two procedures. The global model was used to generate initial training data for paracetamol, aspirin, and squaric acid, and achieves reliable performance on the description of the vibrational properties with the QHA. These preliminary tests show that this framework could be a promising route towards developing an accurate and transferable MLIP for molecular crystals. Nonetheless, this task faces significant challenges, especially for the inclusion of long range interactions and the development of a training set for accurate modelling of complex structures such as salts and co-crystals. For the final re-ranking step, explicit DFT calculations are typically used and our approach could be applied to further accelerate this process. As mentioned above, this will require exploring strategies to extend our framework to predict full energy landscapes for multiple polymorphs with minimal data requirements.

In conclusion, this work demonstrates that employing state-of-the-art MLIPs helps to bridge the gap toward routine accurate modelling of molecular crystals under realistic thermodynamic conditions. We hope that this research will support the pursuit of first-principles accuracy in systems relevant to pharmaceutical and biological studies.

## 6. Methods

The work conducted in this manuscript is based on four fundamental steps. These steps are: (A) generating training set configurations with the MACE-MP-0b3 potential; (B) computing energy, forces, and stress for the training set configurations with DFT; (C) fine tuning the MACE-MP-0b3 potential for the training set from steps (A and B); and (D) computing the sublimation enthalpy with three different approximations: QHA, MD, and PIMD. In this section, we describe theory and computational details for each step.

### 6.1. Training set generation

To generate the training set for the MLIP, we initially computed the EOS of each system in X23 with the vdW-DF2 (ref. 93) functional. Details of the DFT calculations are reported in Sec. 6.2. For each molecular crystal in X23, the initial training set for the MLIP is generated by running short (∼5 ps) classical MD simulations with MACE-MP-0b3 in the NVT ensemble (constant number of particles, volume, and temperature) with small unit cells at each volume of the solid EOS and for the gas phase. The volumes for the EOS were obtained by optimizing with DFT the unit cell at the pressures 0, ±1, ±2, ±4 kbar. The MD simulations are run at a relatively low temperature, *T* ∼ 100 K. The initial training set is obtained by randomly selecting ∼10 structures per volume (∼7 volumes) from the NVT simulations. The training set was subsequently augmented with structures sampled at the volumes *V* where the difference on the EOS between the model and DFT was larger. Finally, the training set was augmented with ∼5 structures per volume obtained from PIMD simulations at higher temperatures *T* ∼ 300 K. Overall, the training set for each molecular crystal comprises ∼200 structures. The exact training set size for each model is reported in Sec. S4 of the ESI.† The MD simulations are performed with the i-PI^94^ code by using the atomistic simulation environment (ASE)^95^ as the force provider.

### 6.2. Density functional theory

The MLIP has been trained on DFT energies, forces, and stresses computed with the vdW-DF2 (ref. 93) functional with VASP.^96–99^ The vdW-DF2 functional was chosen based on the benchmark of the X23 lattice energies against reference DMC values,^30^ reported in Appendix A. In all the DFT calculations, the projector-augmented plane wave method (PAW) has been used with hard pseudo-potentials^100,101^ with a dense FFT grid, a PAW energy cut-off of 1000 eV, and a break condition in the self consistent loop of 10^−7^ eV. The total energy of the solid phase is computed with a dense system specific *k*-point grid that ensures a convergence of each molecular crystal lattice energy with a threshold of 1 meV. The *k*-point grids used for each molecular crystal are reported in Table S1 of the ESI.† The total energies of the gas phase are computed at the *Γ* point in cubic boxes of ∼20 Å.

### 6.3. Fine tuning of MACE-MP-0

The MLIP is obtained by fine tuning the “medium” foundation model MACE-MP-0b3 (the exact starting point is provided on GitHub). In each fine tuning iteration, we train the new model starting from the initial parameters of MACE-MP-0b3. Each optimization cycle is performed with 2000 epochs. The script used to fine-tune the initial model is provided on GitHub.

### 6.4. Sublimation enthalpy

The fine-tuned MLIPs are finally used to compute the sublimation enthalpies of the X23 dataset. The sublimation enthalpy is defined as the difference between the enthalpy of the gas phase (*H*_gas_) and the enthalpy per molecule of the solid (*H*_sol_):

1 Δ*H*_sub_ = *H*_gas_ − *H*_sol_.

The total enthalpy of both gas and solid phases is defined as the sum of the total internal energy and the pressure–volume term:

2 *H* = *E* + *pV*,

where in the following we will assume that the energy *E* and volume *V* relative to the solid phase are always divided by the number of molecules in the cell. In this work, the sublimation enthalpies are computed with three different levels of approximation: quasi-harmonic, anharmonicity with classical nuclei dynamics, and anharmonicity with quantum nuclei dynamics. The sublimation enthalpies are computed at the temperature T* for which experimental estimates of the sublimation enthalpies are available. The temperature T* is room temperature for all the molecular crystals except: acetic acid (T* = 290 K), ammonia (T* = 195 K), benzene (T* = 279 K), carbon dioxide (T* = 207 K) and formamide (T* = 276 K).

#### 6.4.1. Quasi harmonic approximation

Under the ideal gas approximation, the absolute enthalpy of the gas phase *H*_gas_ can be computed as the sum of the electronic energy (*E*^el^_gas_) and the respective terms corresponding to its translational *E*^trans^_gas_, rotational *E*^rot^_gas_, and vibrational degrees of freedom *E*^vib^_gas_, as well as a *pV* term:

3 *H*_gas_ = *E*^el^_gas_ + *E*^vib^_gas_ + *E*^trans^_gas_ + *E*^rot^_gas_ + *pV*.

In the QHA, the vibrational energy *E*^vib^_gas_ is computed from the vibrational frequencies *ω*_*i*_ as:4
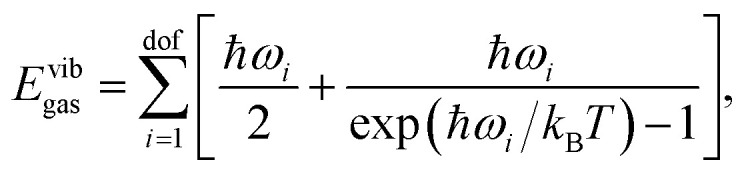
where *k*_B_ is the Boltzmann constant, *T* is the temperature, and dof is the number of degrees of freedom. We note that the zero point energy contribution 
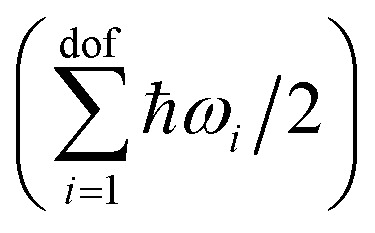
 is explicitly taken into account in the (quantum) QHA. Given the number of atoms in the molecule *N*, the number of degrees of freedom is dof = 3*N* − 6 for non-linear molecules and dof = 3*N* − 5 for linear molecules (only CO_2_ in the X23 dataset). In the ideal gas approximation, we have *E*^trans^_gas_ = (3/2)*RT*, *E*^rot^_gas_ = (3/2)*RT* for non linear molecules and *E*^rot^_gas_ = *RT* for linear molecules, and *pV* = *RT*.

The total enthalpy of the solid phase is computed as the sum of the electronic and vibrational energy and the pV term:

5 *H*_sol_ = *E*^el^_sol_ + *E*^vib^_sol_ + *pV*.

The *pV* term for the solid is usually <0.05 kJ mol^−1^ and is typically neglected. The vibrational energy of the solid in the QHA is computed as:6
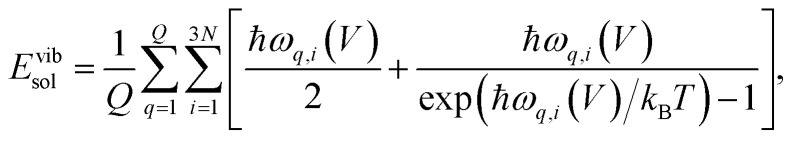
where *N* is the number of atoms in the unit cell, *V* is the volume, *ω*_*q*,*i*_(*V*) are the volume dependent phonon frequencies, and *Q* the total number of the *q*-point grid over which the sum is computed.

In this work, we use the MLIP trained at the vdW-DF2 functional to estimate the vibrational contribution to the sublimation enthalpy, while the zero temperature electronic contribution is given by the DMC reference lattice energy calculations from ref. 30. Hence, the equation used to compute the sublimation enthalpies with the QHA is:

7 Δ*H*^QHA^_sub_ = *E*^el,DMC^_gas_ − *E*^el,DMC^_sol_ + *E*^vib,MLIP^_gas_ − *E*^vib,MLIP^_sol_ + 4*RT*,

except for carbon dioxide, where the *RT* contribution is (7/2)*RT*. We notice in particular that the quantity *E*^el^_gas_ − *E*^el^_sol_ is the negative of the lattice energy *E*_latt_. The lattice energy is used as a measure of relative stabilities in the zero temperature ‘perfect lattice’ approximation and is often the focus of several computational approaches. A breakdown of each contribution to the sublimation enthalpies computed with eqn (7) is reported in Table S30 of the ESI.†

##### 6.4.1.1. Computational details

The vibrational frequencies in the QHA are obtained with the small displacement method using a displacement of ∼0.01 Å. The solid phase vibrational energies are computed with the code PHON.^102^ The reference DFT forces are computed with VASP, while the MLIP forces are obtained with ASE. The reference frequencies and vibrational energies of the gas phase are computed directly with VASP, while the MLIP frequencies and vibrational energies of the gas phase are computed with ASE. The VASP, PHON, and ASE input files used to obtain the vibrational energies are provided on GitHub.

#### 6.4.2. Anharmonicity with a classical description of the nuclei

The anharmonic estimates of the sublimation enthalpies are computed by running classical MD simulations to sample the potential energies of the solid and gas phase. In particular, we run NPT (constant number of atoms, pressure, and temperature) simulations for the solid and NVT simulations for the gas phase. The total enthalpy of the solid phase is then estimated as:

8 〈*H*〉_sol_ = 〈*K*〉^NPT^_sol_ + 〈*U*〉^NPT^_sol_ + *p*〈*V*〉^NPT^_sol_,

where *U* is the potential energy in the NPT simulation. Similarly, the total enthalpy of the gas phase is estimated as:9
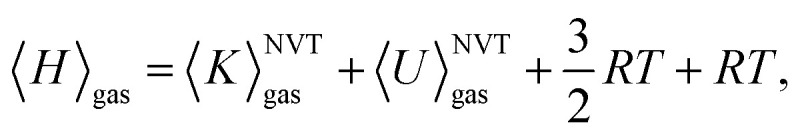
where the (3/2)*RT* is added to take into account the translational energy of the center of mass. Eqn (8) and (9) allow us to estimate the sublimation enthalpy with full anharmonicity with a classical description of the nuclei, by sampling 〈*U*〉 with classical MD simulations.

As for the QHA approximation, we use the MLIP trained at the vdW-DF2 functional to estimate the vibrational contribution to the sublimation enthalpy, while the zero temperature electronic contribution is corrected to the DMC reference lattice energy calculations from ref. 30. Hence, the equation used to compute the sublimation enthalpies with the MD approach is:10
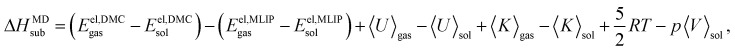
where *E* is the total electronic energy at zero temperature, *U* is the potential energy, and *K* is the classical kinetic energy. The classical estimation of the sublimation enthalpy does not include the zero point energy. A breakdown of each contribution to the sublimation enthalpies computed with eqn (10) is reported in Table S31 of the ESI.†

##### 6.4.2.1. Computational details

The MD simulations are performed with i-PI using a time step of 1 fs and the generalized Langevin equation (GLE) barostat-thermostat. In particular, we run ∼500 ps NPT simulations at *p* ∼ 1 bar and *T* = T* for the solid phase, and ∼1 ns NVT simulations at *T* = T* for the gas phase. The statistical error bar on the averaged quantity were computed with reblocking averaging. Further details on the supercells used for the MD simulations are provided in Table S28 of the ESI.† The input files used for the classical MD simulations are provided on GitHub.

#### 6.4.3. Anharmonicity with a quantum description of the nuclei

The anharmonic estimates of the sublimation enthalpies with a quantum description of the nuclei are computed by PIMD simulations to sample the total energies of the solid and gas phase. In particular, we run NPT (constant number of atoms, pressure, and temperature) simulations for the solid and NVT simulations for the gas phase. The total enthalpy of the solid phase is then estimated as:

11 〈*H*〉_sol_ = 〈*E*〉^NPT^_sol_ + *p*〈*V*〉^NPT^_sol_,

where *E* is the sum of the centroid virial estimator of *K*_cv_^103^ and potential energy *U* in the NPT simulation. Similarly, the total enthalpy of the gas phase is estimated as:

12 〈*H*〉_gas_ = 〈*E*〉^NVT^_gas_ + *RT*.

Eqn (11) and (12) allow us to estimate the sublimation enthalpy with to estimate the sublimation enthalpy with full anharmonicity with a quantum description of the nuclei, by sampling 〈*E*〉 with PIMD simulations.

As for the previous cases, we use the MLIP trained at the vdW-DF2 functional to estimate the vibrational contribution to the sublimation enthalpy, while the zero temperature electronic contribution is corrected to the DMC reference lattice energy calculations from ref. 30. Hence, the equation used to compute the sublimation enthalpies with the PIMD approach is:

13 Δ*H*^PIMD^_sub_ = (*E*^el,DMC^_gas_ − *E*^el,DMC^_sol_) − (*E*^el,MLIP^_gas_ − *E*^el,MLIP^_sol_) + 〈*K*_cv_ + *U*〉_gas_ − 〈*K*_cv_ + *U*〉_sol_ + *RT* − *p*〈*V*〉_sol_,

where *E* is the total energy at zero temperature, *K*_cv_ is the centroid virial estimator of the kinetic energy^103^ and *U* is the potential energy. The centroid virial kinetic energy explicitly takes into account the (3/2)*RT* energy of the center of mass which is therefore is not added explicitly in eqn (13). The quantum statistical estimation of the sublimation enthalpy naturally includes the zero point energy, sampled in the calculation of the potential, *U*, and kinetic energy, *K*_cv_. A breakdown of each contribution to the sublimation enthalpies computed with eqn (13) is reported in Table S32 of the ESI.†

##### 6.4.3.1. Computational details

The PIMD simulations are performed with i-PI using 32 replicas, a time step of 1 fs, the GLE barostat and the path integral Langevin equation (PILE) thermostat.^104^ In particular, we run ∼200 ps NPT simulations at *p* ∼ 1 bar and *T* = T* for the solid phase, and ∼1 ns NVT simulations at *T* = T* for the gas phase. The statistical error bar on the averaged quantity were computed with reblocking averaging. The input files for the PIMD simulations are provided on GitHub.

## Appendices

### Appendix A: Benchmark of DFT functionals against diffusion Monte Carlo

**Fig. 5:**
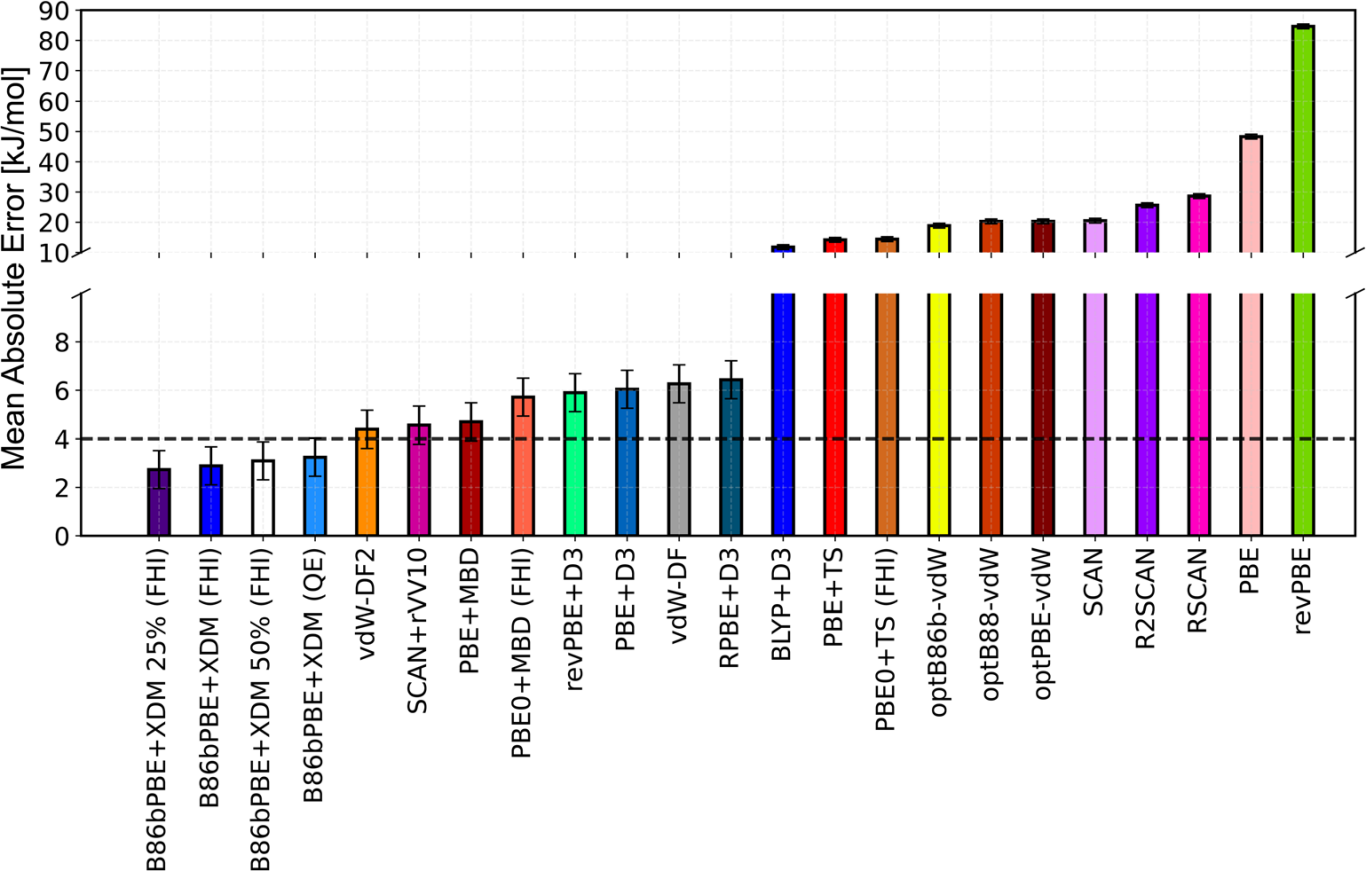
Benchmark of DFT functionals against reference DMC values^30^ on the lattice energies of the X23 dataset. The functionals are listed from left to right in order of decreasing performance (higher MAE). The reported error bar is the average statistical error bar of the DMC reference values. “QE” and “FHI” mean that the number have been respectively computed with Quantum Espresso^105^ or FHI-aims.^106^

The key initial ingredient to train an MLIP that achieves chemical accuracy compared to the experiment is to determine a DFT functional that achieves the desired accuracy. To identify reliable functionals for the description of the X23 molecular crystals, we perform a benchmark of the X23 lattice energies against the reference quantum diffusion Monte Carlo (DMC) estimates from ref. 30. The geometries used in the DFT calculations are the same as those used for the DMC calculations,^30^ which were optimised with the optB88-vdW functional. In Fig. 5, we report the performance of several DFT functionals measured as a Mean Absolute Error (MAE) against the DMC estimates of the lattice energies. The tested functionals are reported in order of decreasing performance (from left to right), *i.e.* higher MAE. The error bar on each column represents the average statistical error bar of the DMC reference values.^30^ The majority of the calculations have been performed with VASP^96–99^ using the same set-up described in the Methods section. The B86bPBE functional with the exchange-hole dipole moment (XDM)^23^ dispersion correction has been tested both with Quantum Espresso^105^ (QE in the figure) with pseudopotentials, and with FHI-aims^106^ (FHI in the figure) with the all electron calculation. The hybrids B86bPBE + XDM with 25% and 50% delocalization correction have been also tested with FHI-aims. Overall, we find that several functionals, namely PBE + MBD, SCAN + rVV10, vdW-DF2, B86bPBE + XDM(50%), B86bPBE + XDM(25%), and B86bPBE + XDM, achieve the chemical accuracy limit with a MAE ∼ 4 kJ mol^−1^. Their performance are approximately equivalent taking into account the DMC statistical error bars. The functional used in this work is vdW-DF2, which was chosen considering its reliable performance and its cost comparable to GGA calculations. We acknowledge that other functionals could have been chosen based on the reported benchmark. However, we notice that: (1) the minimal data strategy and framework proposed in the main manuscript should not be highly dependent on the selected functional (this statement is also supported by the test on the ice polymorphs reported in Sec. S12†); and (2) the choice of the functional defines major differences on the computation of the zero temperature lattice energies (which account for ∼80% of the sublimation enthalpy), and not of the vibrational contribution. This is evident from the comparison between the QHA sublimation enthalpies computed in this work and in previous work, reported in Sec. S11. Since the zero temperature contribution is corrected to the DMC values (as explained in the Methods section and in Sec. S9 of the ESI†), we expect the choice of the DFT functionals (among the reliable ones) to play a minor role in the sublimation enthalpies reported in Table S29.† Further details on the DFT benchmark – including tabulated results for each functional – are reported in Section S1 of the ESI.†

## Data availability

The data supporting the findings of this work, including the training set and the fine-tuned models, together with scripts and input and output are provided on GitHub and in the ESI.†

## Author contributions

Investigation and Data curation: F. D. P. and B. X. S.; methodology and formal analysis: all authors; conceptualization: all authors; project administration: A. M. and V. K.; writing – original draft: F. D. P; writing – review & editing: all authors; resources: A. M., V. K., A. Z., and D. A.

## Conflicts of interest

There are no conflicts to declare.

## Supplementary Material

SC-OLF-D5SC01325A-s001
