# Supplementary material for: Accurate and efficient machine learning interatomic potentials for finite temperature modelling of molecular crystals
Source: Chem Sci. 2025 May 23;16(25):11419–33. doi: 10.1039/d5sc01325a (PMC12101462; doi:10.1039/d5sc01325a)
Supplement: SC-016-D5SC01325A-s001 [file SC-016-D5SC01325A-s001.pdf]

Supplementary Information (SI) for Chemical Science.
This journal is © The Royal Society of Chemistry 2025

Supporting Information: Accurate and efficient machine learning interatomic potentials for finite temperature modelling of molecular crystals

Flaviano Della Pia,[†] Benjamin Shi,[†] Venkat Kapil,[*,†,‡,¶,§] Andrea Zen,[‖,⊥] Dario Alfè,[†,‖,⊥,¶,§] and Angelos Michaelides[*,†]

†*Yusuf Hamied Department of Chemistry, University of Cambridge, Cambridge CB2 1EW, United Kingdom*

‡*Department of Physics and Astronomy, University College London, London, United Kingdom*

¶*Thomas Young Centre, University College London, London WC1E 6BT, United Kingdom*

§*London Centre for Nanotechnology, University College London, London WC1E 6BT, United Kingdom*

‖*Dipartimento di Fisica Ettore Pancini, Università di Napoli Federico II, Monte S. Angelo, I-80126 Napoli, Italy*

⊥*Department of Earth Sciences, University College London, London WC1E 6BT, United Kingdom*

E-mail: v.kapil@ucl.ac.uk; am452@cam.ac.uk

Contents

S1 Benchmark of DFT functionals against diffusion Monte Carlo **S-4**

S2 vdW-DF2 Equations of State **S-28**

S3 Framework Computational Cost **S-31**

S4 Fine tuning errors **S-32**

S5 Computational set-up of the MD simulations **S-33**

S6 Convergence test: size of the training set **S-34**

S7 Convergence test: size of the simulation cell **S-35**

S8 Convergence test: number of beads in the PIMD simulations **S-36**

S9 Sublimation enthalpies **S-37**

S10 Benchmark of the fine tuned models **S-43**

S10.1 Lattice energy S-43

S10.2 Equation of State S-43

S10.3 Quasi-Harmonic vibrations of the solid S-47

S10.4 QHA vibrational contribution to the sublimation enthalpy S-59

S11 QHA sublimation enthalpies: comparison with previous work **S-61**

S12 Ice polymorphs **S-63**

S13 General Model vs System Specific Model **S-67**

S14 Anharmonicity in succinic acid **S-71**

S15 Paracetamol, Aspirin and Squaric acid **S-72**

S15.1 Equation of State S-72

S15.2 QHA vibrational properties S-73

S15.3 Computational details S-77

S15.3.1 Fine tuned models S-77

S15.3.2 Density Functional Theory and QHA S-77

S15.3.3 MD and PIMD sublimation enthalpies S-78

References **S-79**

S1 Benchmark of DFT functionals against diffusion Monte Carlo

In this section we report additional information on the DFT benchmark against DMC lattice energies reported in Appendix A of the main manuscript.

The k-point grids used for the DFT calculations for each molecular crystal are reported in Table S1. To facilitate the reproducibility of our calculations as well as a system specific

Table S1: K-point grid for each molecular crystal in X23 used in the DFT calculations.

System	k-point grid
1,4-cyclohexanedione	$4 \times 4 \times 4$
acetic acid	$2 \times 7 \times 5$
adamantane	$4 \times 4 \times 3$
ammonia	$5 \times 5 \times 5$
anthracene	$4 \times 4 \times 3$
benzene	$4 \times 3 \times 4$
carbon dioxide	$5 \times 5 \times 5$
cyanamide	$4 \times 4 \times 3$
cytosine	$2 \times 3 \times 7$
ethyl carbamate	$6 \times 4 \times 4$
formamide	$7 \times 3 \times 4$
imidazole	$4 \times 5 \times 3$
naphthalene	$4 \times 5 \times 4$
oxalic acid alpha	$4 \times 3 \times 4$
oxalic acid beta	$6 \times 4 \times 5$
pyrazine	$3 \times 5 \times 7$
pyrazole	$3 \times 2 \times 4$
triazine	$3 \times 3 \times 4$
trioxane	$3 \times 3 \times 3$
uracil	$3 \times 2 \times 9$
urea	$5 \times 5 \times 6$
hexamine	$5 \times 5 \times 5$
succinic acid	$3 \times 3 \times 3$

analysis of the tested functionals, in the following we report for each molecular crystal a table with the performance of each functional on the lattice energy. In particular, each table reports the total energies of the solid and the gas (in eV), the lattice energy (in eV and in kJ/mol), and the difference between the prediction of the DFT functional and the DMC

reference[S1] (in kJ/mol), namely the "DFT error".

Table S2: DFT benchmark for 1,4-cyclohexanedione. The table reports the total energy of the solid (eV), gas (eV), the lattice energy (eV and kJ/mol), and the difference between the DFT and the reference DMC lattice energy[S1] (kJ/mol).

DFT functional	Solid [eV]	Gas [eV]	Lattice [eV]	Lattice [kJ/mol]	DFT error [kJ/mol]
B86bPBE+XDM 25% (FHI)	-20913.0471	-10455.6059	-0.9176	-88.53	-0.20
B86bPBE+XDM (FHI)	-20917.5179	-10457.8422	-0.9168	-88.45	-0.12
B86bPBE+XDM 50% (FHI)	-20909.0263	-10453.5831	-0.9300	-89.73	-1.40
B86bPBE+XDM (QE)	-5557.2635	-2777.7212	-0.9106	-87.85	0.48
vdW-DF2	-173.1095	-85.5233	-1.0315	-99.52	-11.18
SCAN+rVV10	-210.2692	-104.1525	-0.9821	-94.76	-6.42
PBE+MBD	-196.7143	-97.4122	-0.9450	-91.17	-2.84
PBE0+MBD (FHI)	-20898.2416	-10448.1445	-0.9764	-94.20	-5.87
revPBE+D3	-193.7562	-95.9394	-0.9387	-90.57	-2.24
PBE+D3	-196.4945	-97.2660	-0.9813	-94.67	-6.34
vdW-DF	-169.1273	-83.5489	-1.0147	-97.90	-9.57
RPBE+D3	-193.2807	-95.7485	-0.8918	-86.04	2.29
BLYP+D3	-189.5952	-93.6829	-1.1147	-107.54	-19.21
PBE+TS	-196.6381	-97.2275	-1.0916	-105.31	-16.98
PBE0+TS (FHI)	-20898.1708	-10447.9772	-1.1083	-106.93	-18.59
optB86b-vdW	-173.4611	-85.5377	-1.1928	-115.08	-26.75
optB88-vdW	-173.8090	-85.6863	-1.2182	-117.54	-29.20
optPBE-vdW	-172.0420	-84.7866	-1.2344	-119.09	-30.76
SCAN	-212.0844	-105.3808	-0.6614	-63.81	24.52
R2SCAN	-208.8836	-103.8462	-0.5956	-57.46	30.87
RSCAN	-216.1216	-107.5025	-0.5582	-53.86	34.47
PBE	-194.7122	-97.0687	-0.2874	-27.73	60.60
revPBE	-190.4507	-95.3882	0.1629	15.71	104.05

Table S3: DFT benchmark for Acetic Acid. The table reports the total energy of the solid (eV), gas (eV), the lattice energy (eV and kJ/mol), and the difference between the DFT and reference DMC lattice energy[S1] (kJ/mol).

DFT functional	Solid [eV]	Gas [eV]	Lattice [eV]	Lattice [kJ/mol]	DFT error [kJ/mol]
B86bPBE+XDM 25% (FHI)	-24963.5610	-6240.1450	-0.7452	-71.90	-0.19
B86bPBE+XDM (FHI)	-24969.6895	-6241.6607	-0.7617	-73.49	-1.78
B86bPBE+XDM 50% (FHI)	-24957.9634	-6238.7513	-0.7395	-71.35	0.36
B86bPBE+XDM (QE)	-6826.9977	-1705.9886	-0.7608	-73.40	-1.69
vdW-DF2	-166.5376	-40.8739	-0.7605	-73.37	-1.66
SCAN+rVV10	-206.5486	-50.8271	-0.8100	-78.15	-6.44
PBE+MBD	-191.1989	-47.0126	-0.7871	-75.94	-4.23
PBE0+MBD (FHI)	-24947.0134	-6235.9643	-0.7890	-76.13	-4.42
revPBE+D3	-188.2673	-46.3660	-0.7009	-67.62	4.09
PBE+D3	-191.1218	-46.9740	-0.8065	-77.81	-6.10
vdW-DF	-162.2580	-39.8204	-0.7441	-71.79	-0.08
RPBE+D3	-187.8890	-46.2837	-0.6885	-66.43	5.28
BLYP+D3	-185.1222	-45.4586	-0.8220	-79.31	-7.60
PBE+TS	-191.1865	-46.9507	-0.8459	-81.62	-9.91
PBE0+TS (FHI)	-24947.0125	-6235.9102	-0.8429	-81.32	-9.61
optB86b-vdW	-166.0289	-40.6026	-0.9046	-87.28	-15.57
optB88-vdW	-166.5337	-40.7228	-0.9106	-87.85	-16.14
optPBE-vdW	-164.9070	-40.3135	-0.9132	-88.11	-16.40
SCAN	-208.6621	-51.5195	-0.6460	-62.33	9.38
R2SCAN	-204.9348	-50.6376	-0.5962	-57.52	14.19
RSCAN	-213.3901	-52.7714	-0.5761	-55.58	16.13
PBE	-189.4543	-46.9287	-0.4349	-41.96	29.75
revPBE	-185.3969	-46.2280	-0.1212	-11.70	60.01

Table S4: DFT benchmark for Adamantane. The table reports the total energy of the solid (eV), gas (eV), the lattice energy (eV and kJ/mol), and the difference between the DFT and reference DMC lattice energy[S1] (kJ/mol).

DFT functional	Solid [eV]	Gas [eV]	Lattice [eV]	Lattice [kJ/mol]	DFT error [kJ/mol]
B86bPBE+XDM 25% (FHI)	-21280.6842	-10639.6346	-0.7075	-68.26	-7.25
B86bPBE+XDM (FHI)	-21283.2749	-10640.9060	-0.7314	-70.56	-9.55
B86bPBE+XDM 50% (FHI)	-21278.4962	-10638.5765	-0.6716	-64.80	-3.78
B86bPBE+XDM (QE)	-5560.9770	-2779.7453	-0.7432	-71.71	-10.69
vdW-DF2	-277.4236	-137.8615	-0.8503	-82.04	-21.02
SCAN+rVV10	-319.0624	-158.8630	-0.6682	-64.47	-3.45
PBE+MBD	-303.8291	-151.1204	-0.7942	-76.62	-15.60
PBE0+MBD (FHI)	-21263.8983	-10631.1479	-0.8013	-77.31	-16.29
revPBE+D3	-300.2396	-149.3533	-0.7665	-73.95	-12.94
PBE+D3	-303.0930	-150.7381	-0.8084	-77.99	-16.98
vdW-DF	-272.2020	-135.2236	-0.8774	-84.65	-23.64
RPBE+D3	-299.7638	-149.1441	-0.7378	-71.18	-10.16
BLYP+D3	-291.9019	-145.0530	-0.8979	-86.63	-25.61
PBE+TS	-303.5942	-150.7118	-1.0853	-104.71	-43.70
PBE0+TS (FHI)	-21263.7089	-10630.7757	-1.0788	-104.08	-43.06
optB86b-vdW	-276.5607	-137.2549	-1.0254	-98.94	-37.92
optB88-vdW	-277.8008	-137.8495	-1.0509	-101.39	-40.38
optPBE-vdW	-275.3058	-136.5664	-1.0865	-104.82	-43.81
SCAN	-321.2009	-160.3480	-0.2524	-24.35	36.66
R2SCAN	-317.6574	-158.6193	-0.2094	-20.21	40.81
RSCAN	-325.5663	-162.6026	-0.1805	-17.42	43.60
PBE	-300.5251	-150.3147	0.0521	5.03	66.05
revPBE	-295.2913	-148.1452	0.4995	48.20	109.21

Table S5: DFT benchmark for Ammonia. The table reports the total energy of the solid (eV), gas (eV), the lattice energy (eV and kJ/mol), and the difference between the DFT and reference DMC lattice energy[S1] (kJ/mol).

DFT functional	Solid [eV]	Gas [eV]	Lattice [eV]	Lattice [kJ/mol]	DFT error [kJ/mol]
B86bPBE+XDM 25% (FHI)	-6163.4505	-1540.4826	-0.3800	-36.67	1.53
B86bPBE+XDM (FHI)	-6164.7457	-1540.7668	-0.4196	-40.48	-2.28
B86bPBE+XDM 50% (FHI)	-6162.2663	-1540.2153	-0.3513	-33.89	4.31
B86bPBE+XDM (QE)	-1738.5123	-434.2031	-0.4249	-41.00	-2.80
vdW-DF2	-76.0250	-18.5947	-0.4115	-39.70	-1.51
SCAN+rVV10	-83.7485	-20.5050	-0.4321	-41.69	-3.49
PBE+MBD	-80.0628	-19.5794	-0.4363	-42.10	-3.90
PBE0+MBD (FHI)	-6158.9611	-1539.3362	-0.4041	-38.99	-0.79
revPBE+D3	-79.9998	-19.6027	-0.3972	-38.32	-0.13
PBE+D3	-80.0794	-19.5663	-0.4535	-43.76	-5.56
vdW-DF	-74.2554	-18.1905	-0.3734	-36.02	2.17
RPBE+D3	-80.0509	-19.6026	-0.4101	-39.57	-1.37
BLYP+D3	-78.3596	-19.1597	-0.4302	-41.51	-3.31
PBE+TS	-80.0697	-19.5660	-0.4514	-43.56	-5.36
PBE0+TS (FHI)	-6158.9724	-1539.3245	-0.4186	-40.38	-2.19
optB86b-vdW	-73.3191	-17.8727	-0.4571	-44.10	-5.90
optB88-vdW	-74.3045	-18.1150	-0.4612	-44.49	-6.29
optPBE-vdW	-73.9872	-18.0325	-0.4643	-44.79	-6.59
SCAN	-84.4599	-20.7428	-0.3721	-35.90	2.29
R2SCAN	-83.6331	-20.5588	-0.3495	-33.72	4.48
RSCAN	-85.9151	-21.1425	-0.3363	-32.44	5.76
PBE	-79.4342	-19.5658	-0.2927	-28.24	9.96
revPBE	-78.8683	-19.5995	-0.1176	-11.35	26.85

Table S6: DFT benchmark for Anthracene. The table reports the total energy of the solid (eV), gas (eV), the lattice energy (eV and kJ/mol), and the difference between the DFT and reference DMC lattice energy[S1] (kJ/mol).

DFT functional	Solid [eV]	Gas [eV]	Lattice [eV]	Lattice [kJ/mol]	DFT error [kJ/mol]
B86bPBE+XDM 25% (FHI)	-29384.0521	-14690.9219	-1.1041	-106.53	-6.31
B86bPBE+XDM (FHI)	-29390.0416	-14693.9568	-1.0640	-102.66	-2.44
B86bPBE+XDM 50% (FHI)	-29378.6070	-14688.1844	-1.1191	-107.98	-7.76
B86bPBE+XDM (QE)	-7378.9875	-3688.4258	-1.0679	-103.03	-2.82
vdW-DF2	-288.4756	-143.1623	-1.0755	-103.77	-3.55
SCAN+rVV10	-349.8081	-173.8730	-1.0311	-99.48	0.74
PBE+MBD	-330.0039	-163.9036	-1.0984	-105.97	-5.75
PBE0+MBD (FHI)	-29361.4880	-14679.5501	-1.1939	-115.18	-14.97
revPBE+D3	-323.2967	-160.4547	-1.1936	-115.16	-14.95
PBE+D3	-329.3060	-163.5345	-1.1185	-107.91	-7.70
vdW-DF	-282.1108	-139.9469	-1.1085	-106.95	-6.73
RPBE+D3	-322.1284	-159.9985	-1.0657	-102.82	-2.60
BLYP+D3	-316.0566	-156.6845	-1.3438	-129.65	-29.44
PBE+TS	-329.8369	-163.5220	-1.3965	-134.73	-34.51
PBE0+TS (FHI)	-29361.3633	-14679.2193	-1.4623	-141.08	-40.87
optB86b-vdW	-292.4835	-144.8390	-1.4028	-135.34	-35.12
optB88-vdW	-292.1458	-144.6508	-1.4221	-137.21	-36.99
optPBE-vdW	-288.6005	-142.8830	-1.4173	-136.74	-36.52
SCAN	-352.3768	-175.6910	-0.4974	-47.99	52.23
R2SCAN	-348.2715	-173.7075	-0.4283	-41.32	58.89
RSCAN	-358.7682	-179.0375	-0.3466	-33.44	66.78
PBE	-326.3776	-163.2455	0.0566	5.47	105.68
revPBE	-318.0740	-159.6846	0.6476	62.48	162.70

Table S7: DFT benchmark for Benzene. The table reports the total energy of the solid (eV), gas (eV), the lattice energy (eV and kJ/mol), and the difference between the DFT and reference DMC lattice energy[S1] (kJ/mol).

DFT functional	Solid [eV]	Gas [eV]	Lattice [eV]	Lattice [kJ/mol]	DFT error [kJ/mol]
B86bPBE+XDM 25% (FHI)	-25297.5758	-6323.8539	-0.5400	-52.10	-2.31
B86bPBE+XDM (FHI)	-25302.4184	-6325.0691	-0.5355	-51.66	-1.88
B86bPBE+XDM 50% (FHI)	-25293.1788	-6322.7596	-0.5351	-51.62	-1.83
B86bPBE+XDM (QE)	-6435.7597	-1608.3990	-0.5409	-52.19	-2.40
vdW-DF2	-274.9384	-68.1705	-0.5641	-54.42	-4.63
SCAN+rVV10	-324.7358	-80.6716	-0.5123	-49.43	0.36
PBE+MBD	-307.5583	-76.3340	-0.5555	-53.60	-3.81
PBE0+MBD (FHI)	-25277.7800	-6318.8608	-0.5842	-56.36	-6.57
revPBE+D3	-302.7566	-75.0834	-0.6057	-58.44	-8.65
PBE+D3	-307.1580	-76.2050	-0.5845	-56.39	-6.61
vdW-DF	-268.6879	-66.5883	-0.5837	-56.31	-6.52
RPBE+D3	-301.9572	-74.9258	-0.5635	-54.37	-4.58
BLYP+D3	-296.1841	-73.3656	-0.6804	-65.64	-15.86
PBE+TS	-307.4993	-76.1896	-0.6852	-66.11	-16.32
PBE0+TS (FHI)	-25277.7407	-6318.7360	-0.6992	-67.46	-17.67
optB86b-vdW	-275.1111	-68.0801	-0.6976	-67.31	-17.52
optB88-vdW	-275.7619	-68.2326	-0.7079	-68.30	-18.51
optPBE-vdW	-273.0064	-67.5167	-0.7349	-70.90	-21.11
SCAN	-327.2222	-81.5296	-0.2760	-26.62	23.16
R2SCAN	-323.7374	-80.6918	-0.2425	-23.40	26.39
RSCAN	-332.8552	-83.0144	-0.1994	-19.24	30.55
PBE	-304.5998	-76.1221	-0.0278	-2.69	47.10
revPBE	-298.2364	-74.8428	0.2837	27.37	77.16

Table S8: DFT benchmark for CO_2. The table reports the total energy of the solid (eV), gas (eV), the lattice energy (eV and kJ/mol), and the difference between the DFT and reference DMC lattice energy[S1] (kJ/mol).

DFT functional	Solid [eV]	Gas [eV]	Lattice [eV]	Lattice [kJ/mol]	DFT error [kJ/mol]
B86bPBE+XDM 25% (FHI)	-20549.9199	-5137.2299	-0.2501	-24.13	5.24
B86bPBE+XDM (FHI)	-20556.1128	-5138.7733	-0.2549	-24.59	4.78
B86bPBE+XDM 50% (FHI)	-20544.1570	-5135.7910	-0.2483	-23.95	5.41
B86bPBE+XDM (QE)	-5557.8356	-1389.2112	-0.2478	-23.90	5.46
vdW-DF2	-72.7245	-17.8416	-0.3395	-32.75	-3.39
SCAN+rVV10	-105.6876	-26.0958	-0.3261	-31.47	-2.10
PBE+MBD	-93.9166	-23.2434	-0.2357	-22.74	6.62
PBE0+MBD (FHI)	-20537.0588	-5134.0186	-0.2461	-23.74	5.62
revPBE+D3	-91.5610	-22.6560	-0.2343	-22.60	6.77
PBE+D3	-93.9683	-23.2322	-0.2598	-25.07	4.30
vdW-DF	-70.3993	-17.2524	-0.3474	-33.51	-4.15
RPBE+D3	-91.2194	-22.5574	-0.2475	-23.88	5.49
BLYP+D3	-90.7293	-22.3721	-0.3102	-29.93	-0.56
PBE+TS	-93.8445	-23.2303	-0.2308	-22.27	7.10
PBE0+TS (FHI)	-20537.0543	-5134.0071	-0.2565	-24.74	4.62
optB86b-vdW	-74.6762	-18.3118	-0.3572	-34.47	-5.10
optB88-vdW	-73.9700	-18.1263	-0.3662	-35.33	-5.97
optPBE-vdW	-73.0666	-17.8495	-0.4171	-40.24	-10.88
SCAN	-107.2057	-26.5702	-0.2312	-22.31	7.06
R2SCAN	-104.5780	-25.9404	-0.2041	-19.69	9.68
RSCAN	-111.1053	-27.6059	-0.1704	-16.44	12.92
PBE	-93.1892	-23.2299	-0.0674	-6.51	22.86
revPBE	-90.1469	-22.6444	0.1077	10.39	39.76

Table S9: DFT benchmark for Cyanamide. The table reports the total energy of the solid (eV), gas (eV), the lattice energy (eV and kJ/mol), and the difference between the DFT and reference DMC lattice energy[S1] (kJ/mol).

DFT functional	Solid [eV]	Gas [eV]	Lattice [eV]	Lattice [kJ/mol]	DFT error [kJ/mol]
B86bPBE+XDM 25% (FHI)	-32423.1879	-4052.0149	-0.8836	-85.25	-1.65
B86bPBE+XDM (FHI)	-32432.3505	-4053.1197	-0.9241	-89.16	-5.56
B86bPBE+XDM 50% (FHI)	-32414.7378	-4050.9900	-0.8523	-82.23	1.37
B86bPBE+XDM (QE)	-8438.6847	-1053.8985	-0.9371	-90.41	-6.81
vdW-DF2	-227.1277	-27.4927	-0.8983	-86.67	-3.07
SCAN+rVV10	-283.3192	-34.4313	-0.9836	-94.90	-11.30
PBE+MBD	-264.2860	-32.0900	-0.9457	-91.25	-7.65
PBE0+MBD (FHI)	-32400.5356	-4049.1514	-0.9155	-88.33	-4.73
revPBE+D3	-259.4566	-31.5582	-0.8738	-84.31	-0.71
PBE+D3	-264.1766	-32.0670	-0.9550	-92.14	-8.54
vdW-DF	-221.0455	-26.7866	-0.8441	-81.44	2.16
RPBE+D3	-258.5357	-31.4679	-0.8491	-81.92	1.68
BLYP+D3	-255.5170	-30.9718	-0.9678	-93.38	-9.78
PBE+TS	-264.2485	-32.0639	-0.9672	-93.32	-9.72
PBE0+TS (FHI)	-32400.4850	-4049.1285	-0.9321	-89.93	-6.33
optB86b-vdW	-228.5772	-27.5456	-1.0266	-99.04	-15.44
optB88-vdW	-228.5794	-27.5421	-1.0303	-99.41	-15.81
optPBE-vdW	-225.9661	-27.2337	-1.0120	-97.64	-14.04
SCAN	-286.0360	-34.9044	-0.8501	-82.02	1.58
R2SCAN	-281.8515	-34.4252	-0.8062	-77.78	5.82
RSCAN	-292.9640	-35.8315	-0.7890	-76.12	7.47
PBE	-261.6495	-32.0559	-0.6503	-62.74	20.86
revPBE	-254.9998	-31.5212	-0.3538	-34.13	49.47

Table S10: DFT benchmark for Cytosine. The table reports the total energy of the solid (eV), gas (eV), the lattice energy (eV and kJ/mol), and the difference between the DFT and reference DMC lattice energy[S1] (kJ/mol).

DFT functional	Solid [eV]	Gas [eV]	Lattice [eV]	Lattice [kJ/mol]	DFT error [kJ/mol]
B86bPBE+XDM 25% (FHI)	-43031.2764	-10756.2026	-1.6165	-155.96	0.26
B86bPBE+XDM (FHI)	-43042.2933	-10758.9484	-1.6250	-156.78	-0.56
B86bPBE+XDM 50% (FHI)	-43021.2651	-10753.7041	-1.6122	-155.54	0.68
B86bPBE+XDM (QE)	-11259.1262	-2813.1676	-1.6139	-155.71	0.51
vdW-DF2	-300.7664	-73.6137	-1.5779	-152.24	3.98
SCAN+rVV10	-380.5278	-93.4502	-1.6817	-162.25	-6.03
PBE+MBD	-353.9635	-86.8369	-1.6540	-159.57	-3.35
PBE0+MBD (FHI)	-43001.6226	-10748.7181	-1.6875	-162.81	-6.59
revPBE+D3	-346.2952	-84.9843	-1.5895	-153.36	2.86
PBE+D3	-353.5698	-86.7127	-1.6797	-162.06	-5.84
vdW-DF	-293.9739	-71.9694	-1.5241	-147.04	9.18
RPBE+D3	-345.0321	-84.7368	-1.5212	-146.77	9.45
BLYP+D3	-339.5615	-83.1186	-1.7718	-170.94	-14.72
PBE+TS	-353.8352	-86.6835	-1.7753	-171.29	-15.07
PBE0+TS (FHI)	-43001.5365	-10748.5852	-1.7989	-173.56	-17.34
optB86b-vdW	-306.9746	-74.8897	-1.8540	-178.87	-22.65
optB88-vdW	-306.0882	-74.6554	-1.8667	-180.10	-23.88
optPBE-vdW	-302.0129	-73.6867	-1.8165	-175.25	-19.03
SCAN	-383.9065	-94.6273	-1.3493	-130.18	26.04
R2SCAN	-377.6332	-93.1336	-1.2747	-122.98	33.24
RSCAN	-392.4995	-96.8834	-1.2415	-119.78	36.44
PBE	-350.2138	-86.5978	-0.9556	-92.20	64.02
revPBE	-340.3748	-84.6486	-0.4451	-42.94	113.28

Table S11: DFT benchmark for Ethyl carbamate. The table reports the total energy of the solid (eV), gas (eV), the lattice energy (eV and kJ/mol), and the difference between the DFT and reference DMC lattice energy[S1] (kJ/mol).

DFT functional	Solid [eV]	Gas [eV]	Lattice [eV]	Lattice [kJ/mol]	DFT error [kJ/mol]
B86bPBE+XDM 25% (FHI)	-17638.6573	-8818.4613	-0.8674	-83.69	0.55
B86bPBE+XDM (FHI)	-17642.7014	-8820.4564	-0.8943	-86.28	-2.04
B86bPBE+XDM 50% (FHI)	-17634.9943	-8816.6489	-0.8482	-81.84	2.40
B86bPBE+XDM (QE)	-4786.0332	-2392.1276	-0.8890	-85.78	-1.54
vdW-DF2	-135.4827	-66.7982	-0.9431	-90.99	-6.75
SCAN+rVV10	-164.5212	-81.3478	-0.9128	-88.07	-3.83
PBE+MBD	-153.3817	-75.7576	-0.9332	-90.04	-5.80
PBE0+MBD (FHI)	-17626.6214	-8812.3792	-0.9316	-89.88	-5.64
revPBE+D3	-151.2329	-74.7608	-0.8556	-82.55	1.69
PBE+D3	-153.2746	-75.6806	-0.9567	-92.31	-8.07
vdW-DF	-132.2699	-65.1901	-0.9448	-91.16	-6.92
RPBE+D3	-150.9834	-74.6399	-0.8518	-82.18	2.06
BLYP+D3	-148.3349	-73.1776	-0.9898	-95.50	-11.26
PBE+TS	-153.2970	-75.6348	-1.0137	-97.80	-13.57
PBE0+TS (FHI)	-17626.5531	-8812.2711	-1.0054	-97.01	-12.77
optB86b-vdW	-134.9192	-66.3760	-1.0836	-104.55	-20.31
optB88-vdW	-135.4243	-66.6172	-1.0949	-105.64	-21.40
optPBE-vdW	-134.1532	-65.9541	-1.1225	-108.30	-24.06
SCAN	-166.1233	-82.3669	-0.6948	-67.03	17.20
R2SCAN	-163.4140	-81.0598	-0.6472	-62.44	21.79
RSCAN	-169.4910	-84.1253	-0.6202	-59.83	24.40
PBE	-152.0750	-75.5727	-0.4648	-44.84	39.39
revPBE	-149.0892	-74.4341	-0.1105	-10.66	73.58

Table S12: DFT benchmark for Formamide. The table reports the total energy of the solid (eV), gas (eV), the lattice energy (eV and kJ/mol), and the difference between the DFT and reference DMC lattice energy[S1] (kJ/mol).

DFT functional	Solid [eV]	Gas [eV]	Lattice [eV]	Lattice [kJ/mol]	DFT error [kJ/mol]
B86bPBE+XDM 25% (FHI)	-18513.8290	-4627.6464	-0.8108	-78.23	2.72
B86bPBE+XDM (FHI)	-18518.7032	-4628.8417	-0.8341	-80.48	0.48
B86bPBE+XDM 50% (FHI)	-18509.3799	-4626.5495	-0.7955	-76.75	4.20
B86bPBE+XDM (QE)	-5021.1843	-1254.4646	-0.8315	-80.22	0.73
vdW-DF2	-126.7502	-30.8642	-0.8233	-79.43	1.52
SCAN+rVV10	-156.2986	-38.1990	-0.8757	-84.48	-3.53
PBE+MBD	-145.2515	-35.4648	-0.8480	-81.82	-0.87
PBE0+MBD (FHI)	-18501.4575	-4624.5209	-0.8435	-81.38	-0.42
revPBE+D3	-143.1169	-34.9952	-0.7841	-75.65	5.31
PBE+D3	-145.2607	-35.4389	-0.8763	-84.55	-3.59
vdW-DF	-123.4541	-30.0854	-0.7781	-75.08	5.88
RPBE+D3	-142.8163	-34.9338	-0.7703	-74.31	6.64
BLYP+D3	-140.8384	-34.3175	-0.8921	-86.07	-5.12
PBE+TS	-145.2501	-35.4268	-0.8858	-85.46	-4.51
PBE0+TS (FHI)	-18501.4631	-4624.4879	-0.8779	-84.70	-3.75
optB86b-vdW	-126.3585	-30.6587	-0.9309	-89.81	-8.86
optB88-vdW	-126.7504	-30.7464	-0.9412	-90.80	-9.85
optPBE-vdW	-125.4914	-30.4423	-0.9306	-89.78	-8.83
SCAN	-157.8752	-38.7241	-0.7447	-71.85	9.10
R2SCAN	-155.1603	-38.0868	-0.7033	-67.85	13.10
RSCAN	-161.4815	-39.6873	-0.6831	-65.91	15.05
PBE	-143.9283	-35.4188	-0.5633	-54.34	26.61
revPBE	-140.8192	-34.9234	-0.2814	-27.15	53.80

Table S13: DFT benchmark for Imidazole. The table reports the total energy of the solid (eV), gas (eV), the lattice energy (eV and kJ/mol), and the difference between the DFT and reference DMC lattice energy[S1] (kJ/mol).

DFT functional	Solid [eV]	Gas [eV]	Lattice [eV]	Lattice [kJ/mol]	DFT error [kJ/mol]
B86bPBE+XDM 25% (FHI)	-24645.7674	-6160.5225	-0.9194	-88.70	-0.50
B86bPBE+XDM (FHI)	-24651.7794	-6162.0023	-0.9425	-90.93	-2.73
B86bPBE+XDM 50% (FHI)	-24640.2420	-6159.1659	-0.8946	-86.31	1.89
B86bPBE+XDM (QE)	-6366.0678	-1590.5768	-0.9401	-90.70	-2.50
vdW-DF2	-204.7387	-50.2828	-0.9019	-87.01	1.19
SCAN+rVV10	-251.4459	-61.9395	-0.9220	-88.96	-0.75
PBE+MBD	-236.3012	-58.1196	-0.9557	-92.21	-4.01
PBE0+MBD (FHI)	-24627.9845	-6156.0367	-0.9594	-92.56	-4.36
revPBE+D3	-231.6814	-57.0161	-0.9043	-87.24	0.96
PBE+D3	-236.0247	-58.0408	-0.9653	-93.13	-4.93
vdW-DF	-200.5533	-49.2464	-0.8919	-86.05	2.15
RPBE+D3	-230.9080	-56.8541	-0.8729	-84.21	3.99
BLYP+D3	-226.5930	-55.6581	-0.9902	-95.53	-7.33
PBE+TS	-236.3184	-58.0358	-1.0438	-100.70	-12.50
PBE0+TS (FHI)	-24628.0067	-6155.9641	-1.0376	-100.11	-11.91
optB86b-vdW	-207.7940	-50.8826	-1.0659	-102.84	-14.64
optB88-vdW	-207.5176	-50.8092	-1.0702	-103.26	-15.05
optPBE-vdW	-205.0996	-50.2085	-1.0664	-102.89	-14.68
SCAN	-253.6027	-62.6828	-0.7179	-69.26	18.94
R2SCAN	-250.1759	-61.8631	-0.6809	-65.69	22.51
RSCAN	-258.8452	-64.0564	-0.6549	-63.19	25.02
PBE	-234.0474	-57.9966	-0.5152	-49.71	38.50
revPBE	-228.2998	-56.8774	-0.1975	-19.06	69.14

Table S14: DFT benchmark for Naphthalene. The table reports the total energy of the solid (eV), gas (eV), the lattice energy (eV and kJ/mol), and the difference between the DFT and reference DMC lattice energy[S1] (kJ/mol).

DFT functional	Solid [eV]	Gas [eV]	Lattice [eV]	Lattice [kJ/mol]	DFT error [kJ/mol]
B86bPBE+XDM 25% (FHI)	-21016.5875	-10507.4730	-0.8207	-79.19	-3.69
B86bPBE+XDM (FHI)	-21020.7599	-10509.5860	-0.7940	-76.61	-1.11
B86bPBE+XDM 50% (FHI)	-21012.7952	-10505.5684	-0.8292	-80.00	-4.51
B86bPBE+XDM (QE)	-5298.5720	-2648.4853	-0.8007	-77.26	-1.76
vdW-DF2	-213.0928	-105.7385	-0.8079	-77.95	-2.45
SCAN+rVV10	-256.2534	-127.3497	-0.7770	-74.97	0.53
PBE+MBD	-242.0399	-120.1942	-0.8257	-79.67	-4.17
PBE0+MBD (FHI)	-21000.3657	-10499.2935	-0.8893	-85.80	-10.31
revPBE+D3	-237.4827	-117.8416	-0.8997	-86.81	-11.31
PBE+D3	-241.5635	-119.9430	-0.8387	-80.92	-5.43
vdW-DF	-208.3294	-103.3399	-0.8248	-79.57	-4.08
RPBE+D3	-236.6813	-117.5344	-0.8063	-77.79	-2.29
BLYP+D3	-232.2180	-115.0970	-1.0121	-97.64	-22.15
PBE+TS	-241.9366	-119.9283	-1.0400	-100.34	-24.84
PBE0+TS (FHI)	-21000.2904	-10499.0622	-1.0831	-104.49	-29.00
optB86b-vdW	-215.1665	-106.5329	-1.0503	-101.33	-25.84
optB88-vdW	-215.1615	-106.5147	-1.0660	-102.85	-27.35
optPBE-vdW	-212.6808	-105.2727	-1.0677	-103.01	-27.52
SCAN	-258.1410	-128.6891	-0.3814	-36.80	38.70
R2SCAN	-255.2104	-127.2800	-0.3253	-31.38	44.11
RSCAN	-262.7383	-131.1065	-0.2626	-25.34	50.16
PBE	-239.4202	-119.7591	0.0490	4.73	80.22
revPBE	-233.6372	-117.3388	0.5202	50.18	125.68

Table S15: DFT benchmark for Oxalic Acid α. The table reports the total energy of the solid (eV), gas (eV), the lattice energy (eV and kJ/mol), and the difference between the DFT and reference DMC lattice energy[S1] (kJ/mol).

DFT functional	Solid [eV]	Gas [eV]	Lattice [eV]	Lattice [kJ/mol]	DFT error [kJ/mol]
B86bPBE+XDM 25% (FHI)	-41229.0016	-10306.2692	-0.9812	-94.67	7.96
B86bPBE+XDM (FHI)	-41240.5804	-10309.1754	-0.9697	-93.56	9.07
B86bPBE+XDM 50% (FHI)	-41218.3325	-10303.5737	-1.0094	-97.39	5.24
B86bPBE+XDM (QE)	-11244.1336	-2810.0761	-0.9573	-92.36	10.27
vdW-DF2	-173.1374	-42.2054	-1.0790	-104.10	-1.47
SCAN+rVV10	-240.5857	-58.9903	-1.1562	-111.55	-8.92
PBE+MBD	-216.0667	-53.0508	-0.9659	-93.19	9.44
PBE0+MBD (FHI)	-41203.2309	-10299.7933	-1.0145	-97.88	4.75
revPBE+D3	-210.4551	-51.7516	-0.8621	-83.18	19.45
PBE+D3	-215.9410	-52.9989	-0.9864	-95.17	7.46
vdW-DF	-167.9368	-40.9840	-1.0002	-96.50	6.13
RPBE+D3	-209.6552	-51.5948	-0.8190	-79.02	23.61
BLYP+D3	-207.9722	-50.8972	-1.0959	-105.73	-3.10
PBE+TS	-215.9398	-52.9720	-1.0130	-97.73	4.90
PBE0+TS (FHI)	-41203.1207	-10299.7251	-1.0551	-101.80	0.83
optB86b-vdW	-177.5045	-43.1662	-1.2100	-116.74	-14.11
optB88-vdW	-176.5346	-42.8934	-1.2403	-119.66	-17.03
optPBE-vdW	-173.9019	-42.2548	-1.2207	-117.77	-15.14
SCAN	-243.4916	-59.9584	-0.9145	-88.23	14.40
R2SCAN	-237.4550	-58.5317	-0.8320	-80.27	22.36
RSCAN	-250.9568	-61.9358	-0.8034	-77.51	25.12
PBE	-213.8385	-52.9474	-0.5122	-49.42	53.21
revPBE	-206.6470	-51.5765	-0.0852	-8.22	94.41

Table S16: DFT benchmark for Oxalic Acid β. The table reports the total energy of the solid (eV), gas (eV), the lattice energy (eV and kJ/mol), and the difference between the DFT and reference DMC lattice energy[S1] (kJ/mol).

DFT functional	Solid [eV]	Gas [eV]	Lattice [eV]	Lattice [kJ/mol]	DFT error [kJ/mol]
B86bPBE+XDM 25% (FHI)	-20614.5052	-10306.2692	-0.9835	-94.88	7.42
B86bPBE+XDM (FHI)	-20620.3550	-10309.1754	-1.0021	-96.68	5.62
B86bPBE+XDM 50% (FHI)	-20609.1163	-10303.5737	-0.9844	-94.98	7.32
B86bPBE+XDM (QE)	-5622.1300	-2810.0761	-0.9889	-95.41	6.89
vdW-DF2	-86.5370	-42.2054	-1.0631	-102.57	-0.27
SCAN+rVV10	-120.3167	-58.9903	-1.1681	-112.70	-10.40
PBE+MBD	-108.1053	-53.0508	-1.0019	-96.66	5.64
PBE0+MBD (FHI)	-20601.6230	-10299.7933	-1.0182	-98.24	4.06
revPBE+D3	-105.3144	-51.7516	-0.9055	-87.37	14.93
PBE+D3	-108.0419	-52.9989	-1.0221	-98.61	3.69
vdW-DF	-83.9896	-40.9840	-1.0108	-97.52	4.78
RPBE+D3	-104.9091	-51.5948	-0.8598	-82.95	19.35
BLYP+D3	-104.0342	-50.8972	-1.1199	-108.05	-5.75
PBE+TS	-108.0495	-52.9720	-1.0528	-101.57	0.73
PBE0+TS (FHI)	-20601.5693	-10299.7251	-1.0596	-102.23	0.07
optB86b-vdW	-88.8054	-43.1662	-1.2366	-119.30	-17.00
optB88-vdW	-88.3010	-42.8934	-1.2571	-121.29	-18.99
optPBE-vdW	-86.9833	-42.2548	-1.2369	-119.33	-17.03
SCAN	-121.7750	-59.9584	-0.9291	-89.64	12.66
R2SCAN	-118.7583	-58.5317	-0.8474	-81.76	20.54
RSCAN	-125.5119	-61.9358	-0.8201	-79.13	23.17
PBE	-107.0006	-52.9474	-0.5529	-53.34	48.96
revPBE	-103.4192	-51.5765	-0.1331	-12.84	89.46

Table S17: DFT benchmark for Pyrazine. The table reports the total energy of the solid (eV), gas (eV), the lattice energy (eV and kJ/mol), and the difference between the DFT and reference DMC lattice energy[S1] (kJ/mol).

DFT functional	Solid [eV]	Gas [eV]	Lattice [eV]	Lattice [kJ/mol]	DFT error [kJ/mol]
B86bPBE+XDM 25% (FHI)	-14396.6848	-7197.7068	-0.6356	-61.32	-0.25
B86bPBE+XDM (FHI)	-14400.2641	-7199.4815	-0.6506	-62.77	-1.69
B86bPBE+XDM 50% (FHI)	-14393.3795	-7196.0715	-0.6182	-59.64	1.43
B86bPBE+XDM (QE)	-3685.1640	-1841.9337	-0.6483	-62.55	-1.47
vdW-DF2	-116.3075	-57.4697	-0.6840	-65.99	-4.92
SCAN+rVV10	-143.6370	-71.1993	-0.6192	-59.74	1.34
PBE+MBD	-134.6062	-66.6643	-0.6387	-61.63	-0.55
PBE0+MBD (FHI)	-14386.1372	-7192.4169	-0.6517	-62.88	-1.81
revPBE+D3	-131.9567	-65.3242	-0.6542	-63.12	-2.04
PBE+D3	-134.5040	-66.5652	-0.6868	-66.26	-5.19
vdW-DF	-113.6929	-56.1669	-0.6795	-65.56	-4.49
RPBE+D3	-131.5465	-65.1470	-0.6263	-60.42	0.65
BLYP+D3	-129.3182	-63.8964	-0.7627	-73.59	-12.51
PBE+TS	-134.6357	-66.5491	-0.7687	-74.17	-13.09
PBE0+TS (FHI)	-14386.1800	-7192.3174	-0.7726	-74.54	-13.46
optB86b-vdW	-117.8706	-58.1253	-0.8100	-78.15	-17.07
optB88-vdW	-117.7097	-58.0300	-0.8248	-79.58	-18.50
optPBE-vdW	-116.3321	-57.3263	-0.8398	-81.02	-19.95
SCAN	-144.8806	-72.0545	-0.3858	-37.22	23.85
R2SCAN	-142.8863	-71.0963	-0.3469	-33.47	27.61
RSCAN	-147.8777	-73.6245	-0.3144	-30.33	30.74
PBE	-133.3291	-66.5047	-0.1598	-15.42	45.65
revPBE	-129.9504	-65.1382	0.1630	15.73	76.80

Table S18: DFT benchmark for Pyrazole. The table reports the total energy of the solid (eV), gas (eV), the lattice energy (eV and kJ/mol), and the difference between the DFT and reference DMC lattice energy[S1] (kJ/mol).

DFT functional	Solid [eV]	Gas [eV]	Lattice [eV]	Lattice [kJ/mol]	DFT error [kJ/mol]
B86bPBE+XDM 25% (FHI)	-49287.0087	-6160.0727	-0.8033	-77.51	-0.19
B86bPBE+XDM (FHI)	-49299.3433	-6161.5984	-0.8195	-79.07	-1.75
B86bPBE+XDM 50% (FHI)	-49275.6292	-6158.6657	-0.7880	-76.02	1.30
B86bPBE+XDM (QE)	-12727.9014	-1590.1689	-0.8187	-78.99	-1.67
vdW-DF2	-405.1723	-49.8596	-0.7869	-75.92	1.40
SCAN+rVV10	-498.3193	-61.4767	-0.8132	-78.46	-1.14
PBE+MBD	-468.4155	-57.7124	-0.8396	-81.00	-3.68
PBE0+MBD (FHI)	-49251.4965	-6155.5873	-0.8498	-81.99	-4.67
revPBE+D3	-459.4279	-56.6136	-0.8149	-78.62	-1.30
PBE+D3	-467.8670	-57.6350	-0.8484	-81.85	-4.53
vdW-DF	-396.8138	-48.8319	-0.7698	-74.27	3.05
RPBE+D3	-457.8577	-56.4536	-0.7786	-75.12	2.20
BLYP+D3	-449.0102	-55.2356	-0.8907	-85.94	-8.62
PBE+TS	-468.3095	-57.6292	-0.9095	-87.75	-10.43
PBE0+TS (FHI)	-49251.3948	-6155.5152	-0.9091	-87.71	-10.39
optB86b-vdW	-411.3516	-50.4696	-0.9493	-91.59	-14.27
optB88-vdW	-410.7346	-50.3878	-0.9540	-92.04	-14.72
optPBE-vdW	-405.9274	-49.7934	-0.9475	-91.42	-14.10
SCAN	-502.6238	-62.2199	-0.6080	-58.66	18.66
R2SCAN	-496.0114	-61.4311	-0.5703	-55.02	22.30
RSCAN	-513.3175	-63.6207	-0.5440	-52.48	24.84
PBE	-463.8669	-57.5906	-0.3927	-37.89	39.43
revPBE	-452.3599	-56.4764	-0.0686	-6.62	70.70

Table S19: DFT benchmark for Triazine. The table reports the total energy of the solid (eV), gas (eV), the lattice energy (eV and kJ/mol), and the difference between the DFT and reference DMC lattice energy[S1] (kJ/mol).

DFT functional	Solid [eV]	Gas [eV]	Lattice [eV]	Lattice [kJ/mol]	DFT error [kJ/mol]
B86bPBE+XDM 25% (FHI)	-45813.8482	-7635.0479	-0.5934	-57.25	3.23
B86bPBE+XDM (FHI)	-45825.8292	-7637.0387	-0.5995	-57.84	2.64
B86bPBE+XDM 50% (FHI)	-45802.8373	-7633.2164	-0.5898	-56.90	3.58
B86bPBE+XDM (QE)	-11757.8640	-1959.0492	-0.5948	-57.38	3.10
vdW-DF2	-318.6437	-52.4376	-0.6697	-64.61	-4.13
SCAN+rVV10	-404.7425	-66.8708	-0.5862	-56.56	3.92
PBE+MBD	-376.6175	-62.1852	-0.5843	-56.38	4.10
PBE0+MBD (FHI)	-45781.2778	-7629.6112	-0.6017	-58.06	2.42
revPBE+D3	-368.4383	-60.8007	-0.6057	-58.44	2.04
PBE+D3	-376.4182	-62.0965	-0.6398	-61.73	-1.25
vdW-DF	-311.8195	-51.2885	-0.6814	-65.74	-5.27
RPBE+D3	-367.2254	-60.6150	-0.5892	-56.84	3.63
BLYP+D3	-361.1781	-59.4879	-0.7085	-68.36	-7.88
PBE+TS	-376.6410	-62.0822	-0.6913	-66.70	-6.22
PBE0+TS (FHI)	-45781.3463	-7629.5224	-0.7020	-67.73	-7.25
optB86b-vdW	-325.5228	-53.4900	-0.7638	-73.70	-13.22
optB88-vdW	-324.2662	-53.2645	-0.7799	-75.24	-14.76
optPBE-vdW	-320.2859	-52.5675	-0.8135	-78.48	-18.01
SCAN	-408.5585	-67.7245	-0.3686	-35.56	24.91
R2SCAN	-402.1704	-66.6980	-0.3304	-31.88	28.60
RSCAN	-417.7638	-69.3285	-0.2988	-28.83	31.65
PBE	-373.2659	-62.0485	-0.1625	-15.68	44.80
revPBE	-363.0986	-60.6395	0.1231	11.88	72.36

Table S20: DFT benchmark for Trioxane. The table reports the total energy of the solid (eV), gas (eV), the lattice energy (eV and kJ/mol), and the difference between the DFT and reference DMC lattice energy[S1] (kJ/mol).

DFT functional	Solid [eV]	Gas [eV]	Lattice [eV]	Lattice [kJ/mol]	DFT error [kJ/mol]
B86bPBE+XDM 25% (FHI)	-56154.4772	-9358.4532	-0.6263	-60.43	1.71
B86bPBE+XDM (FHI)	-56167.5645	-9360.6174	-0.6434	-62.07	0.06
B86bPBE+XDM 50% (FHI)	-56142.5698	-9356.4788	-0.6162	-59.45	2.69
B86bPBE+XDM (QE)	-15346.5669	-2557.1386	-0.6225	-60.06	2.07
vdW-DF2	-359.7955	-59.2110	-0.7549	-72.84	-10.70
SCAN+rVV10	-451.4567	-74.6066	-0.6362	-61.38	0.75
PBE+MBD	-415.9912	-68.6841	-0.6478	-62.50	-0.36
PBE0+MBD (FHI)	-56117.2775	-9352.2108	-0.6688	-64.52	-2.39
revPBE+D3	-409.1778	-67.5848	-0.6115	-58.99	3.14
PBE+D3	-415.5390	-68.5906	-0.6659	-64.25	-2.11
vdW-DF	-350.9160	-57.7418	-0.7442	-71.80	-9.66
RPBE+D3	-408.4946	-67.4882	-0.5942	-57.33	4.81
BLYP+D3	-401.4427	-66.1426	-0.7645	-73.76	-11.62
PBE+TS	-415.8677	-68.5415	-0.7697	-74.26	-12.13
PBE0+TS (FHI)	-56117.1854	-9352.0865	-0.7777	-75.03	-12.90
optB86b-vdW	-360.5614	-59.2631	-0.8305	-80.12	-17.99
optB88-vdW	-361.4181	-59.3773	-0.8590	-82.88	-20.74
optPBE-vdW	-357.4491	-58.6848	-0.8901	-85.88	-23.74
SCAN	-456.0540	-75.6104	-0.3986	-38.46	23.68
R2SCAN	-446.8965	-74.1254	-0.3573	-34.48	27.66
RSCAN	-465.7127	-77.2889	-0.3298	-31.82	30.31
PBE	-411.8929	-68.4831	-0.1657	-15.99	46.15
revPBE	-402.2910	-67.2112	0.1627	15.69	77.83

Table S21: DFT benchmark for Uracil. The table reports the total energy of the solid (eV), gas (eV), the lattice energy (eV and kJ/mol), and the difference between the DFT and reference DMC lattice energy[S1] (kJ/mol).

DFT functional	Solid [eV]	Gas [eV]	Lattice [eV]	Lattice [kJ/mol]	DFT error [kJ/mol]
B86bPBE+XDM 25% (FHI)	-45199.0011	-11298.3611	-1.3892	-134.03	0.24
B86bPBE+XDM (FHI)	-45210.8695	-11301.3233	-1.3941	-134.50	-0.23
B86bPBE+XDM 50% (FHI)	-45188.1829	-11295.6578	-1.3879	-133.91	0.36
B86bPBE+XDM (QE)	-11927.0006	-2980.3718	-1.3784	-132.98	1.28
vdW-DF2	-279.1562	-68.4043	-1.3847	-133.60	0.67
SCAN+rVV10	-361.0898	-88.8246	-1.4479	-139.69	-5.42
PBE+MBD	-333.4707	-81.9523	-1.4153	-136.55	-2.28
PBE0+MBD (FHI)	-45168.5867	-11290.6993	-1.4473	-139.64	-5.37
revPBE+D3	-325.7189	-80.0981	-1.3316	-128.47	5.79
PBE+D3	-333.1778	-81.8444	-1.4500	-139.90	-5.63
vdW-DF	-272.5461	-66.7916	-1.3449	-129.75	4.51
RPBE+D3	-324.5113	-79.8449	-1.2829	-123.78	10.49
BLYP+D3	-319.8705	-78.4387	-1.5289	-147.51	-13.24
PBE+TS	-333.3320	-81.8068	-1.5262	-147.25	-12.98
PBE0+TS (FHI)	-45168.5009	-11290.5742	-1.5510	-149.64	-15.38
optB86b-vdW	-285.7444	-69.8163	-1.6198	-156.28	-22.01
optB88-vdW	-284.5760	-69.5106	-1.6334	-157.59	-23.33
optPBE-vdW	-280.6692	-68.5608	-1.6065	-155.00	-20.73
SCAN	-364.5844	-90.0050	-1.1411	-110.09	24.17
R2SCAN	-357.9578	-88.4191	-1.0704	-103.27	31.00
RSCAN	-373.3249	-92.2956	-1.0356	-99.92	34.35
PBE	-330.1016	-81.7298	-0.7956	-76.76	57.51
revPBE	-320.3765	-79.7675	-0.3266	-31.51	102.75

Table S22: DFT benchmark for Urea. The table reports the total energy of the solid (eV), gas (eV), the lattice energy (eV and kJ/mol), and the difference between the DFT and reference DMC lattice energy[S1] (kJ/mol).

DFT functional	Solid [eV]	Gas [eV]	Lattice [eV]	Lattice [kJ/mol]	DFT error [kJ/mol]
B86bPBE+XDM 25% (FHI)	-12274.1814	-6136.0181	-1.0726	-103.48	5.05
B86bPBE+XDM (FHI)	-12277.2302	-6137.5254	-1.0897	-105.14	3.40
B86bPBE+XDM 50% (FHI)	-12271.4105	-6134.6362	-1.0691	-103.14	5.39
B86bPBE+XDM (QE)	-3315.3909	-1656.6028	-1.0927	-105.42	3.11
vdW-DF2	-85.5647	-41.7060	-1.0764	-103.85	4.68
SCAN+rVV10	-105.9400	-51.7931	-1.1769	-113.55	-5.01
PBE+MBD	-98.4862	-48.1122	-1.1309	-109.11	-0.58
PBE0+MBD (FHI)	-12266.0204	-6131.8763	-1.1339	-109.40	-0.86
revPBE+D3	-96.8876	-47.4085	-1.0352	-99.88	8.65
PBE+D3	-98.4248	-48.0733	-1.1391	-109.90	-1.36
vdW-DF	-83.4658	-40.7406	-0.9923	-95.74	12.80
RPBE+D3	-96.6732	-47.3123	-1.0243	-98.82	9.71
BLYP+D3	-95.1870	-46.4260	-1.1675	-112.64	-4.11
PBE+TS	-98.4099	-48.0509	-1.1540	-111.34	-2.81
PBE0+TS (FHI)	-12265.9490	-6131.8230	-1.1515	-111.09	-2.56
optB86b-vdW	-85.8477	-41.7055	-1.2183	-117.54	-9.01
optB88-vdW	-86.0025	-41.7697	-1.2315	-118.82	-10.28
optPBE-vdW	-85.0542	-41.3295	-1.1976	-115.55	-7.01
SCAN	-106.9786	-52.4814	-1.0079	-97.24	11.30
R2SCAN	-105.1641	-51.6359	-0.9461	-91.28	17.25
RSCAN	-109.4064	-53.7733	-0.9299	-89.72	18.82
PBE	-97.5773	-48.0295	-0.7591	-73.24	35.29
revPBE	-95.3225	-47.2661	-0.3952	-38.13	70.41

Table S23: DFT benchmark for Hexamine. The table reports the total energy of the solid (eV), gas (eV), the lattice energy (eV and kJ/mol), and the difference between the DFT and reference DMC lattice energy[S1] (kJ/mol).

DFT functional	Solid [eV]	Gas [eV]	Lattice [eV]	Lattice [kJ/mol]	DFT error [kJ/mol]
B86bPBE+XDM 25% (FHI)	-12387.2632	-12386.3512	-0.9119	-87.98	-1.80
B86bPBE+XDM (FHI)	-12389.6271	-12388.7202	-0.9069	-87.50	-1.31
B86bPBE+XDM 50% (FHI)	-12385.1778	-12384.2630	-0.9148	-88.26	-2.07
B86bPBE+XDM (QE)	-3246.7591	-3245.8630	-0.8960	-86.45	-0.26
vdW-DF2	-116.4531	-115.4924	-0.9606	-92.68	-6.50
SCAN+rVV10	-139.9217	-139.0197	-0.9020	-87.03	-0.84
PBE+MBD	-131.7175	-130.7926	-0.9249	-89.23	-3.05
PBE0+MBD (FHI)	-12378.2349	-12377.2613	-0.9736	-93.93	-7.75
revPBE+D3	-129.6420	-128.7384	-0.9036	-87.18	-0.99
PBE+D3	-131.4349	-130.4835	-0.9514	-91.79	-5.61
vdW-DF	-114.3097	-113.3458	-0.9639	-93.00	-6.81
RPBE+D3	-129.3507	-128.5056	-0.8451	-81.53	4.65
BLYP+D3	-126.1934	-125.1477	-1.0458	-100.89	-14.71
PBE+TS	-131.6033	-130.4174	-1.1859	-114.42	-28.23
PBE0+TS (FHI)	-12378.1352	-12376.9281	-1.2071	-116.46	-30.28
optB86b-vdW	-117.6188	-116.4523	-1.1665	-112.54	-26.36
optB88-vdW	-117.7512	-116.5558	-1.1954	-115.33	-29.14
optPBE-vdW	-116.4285	-115.2353	-1.1932	-115.12	-28.93
SCAN	-140.9896	-140.4984	-0.4912	-47.39	38.80
R2SCAN	-138.9658	-138.5318	-0.4340	-41.87	44.31
RSCAN	-143.3719	-142.9638	-0.4081	-39.37	46.81
PBE	-130.2695	-130.1425	-0.1269	-12.24	73.94
revPBE	-127.3608	-127.6933	0.3325	32.08	118.26

Table S24: DFT benchmark for Succinic Acid. The table reports the total energy of the solid (eV), gas (eV), the lattice energy (eV and kJ/mol), and the difference between the DFT and reference DMC lattice energy[S1] (kJ/mol).

DFT functional	Solid [eV]	Gas [eV]	Lattice [eV]	Lattice [kJ/mol]	DFT error [kJ/mol]
B86bPBE+XDM 25% (FHI)	-99592.1500	-12447.6937	-1.3250	-127.84	-2.68
B86bPBE+XDM (FHI)	-99616.9649	-12450.7672	-1.3534	-130.58	-5.42
B86bPBE+XDM 50% (FHI)	-99569.5155	-12444.8718	-1.3177	-127.13	-1.97
B86bPBE+XDM (QE)	-27046.2497	-3379.4390	-1.3422	-129.49	-4.33
vdW-DF2	-598.7653	-73.5525	-1.2932	-124.76	0.40
SCAN+rVV10	-765.5622	-94.2066	-1.4887	-143.63	-18.47
PBE+MBD	-704.2155	-86.6282	-1.3987	-134.95	-9.79
PBE0+MBD (FHI)	-99527.0244	-12439.4689	-1.4092	-135.96	-10.80
revPBE+D3	-690.9196	-85.1172	-1.2477	-120.38	4.78
PBE+D3	-703.7190	-86.5421	-1.4227	-137.27	-12.11
vdW-DF	-583.5080	-71.6768	-1.2617	-121.73	3.43
RPBE+D3	-688.8032	-84.9166	-1.1838	-114.21	10.95
BLYP+D3	-679.2443	-83.4258	-1.4797	-142.76	-17.60
PBE+TS	-703.9973	-86.4857	-1.5140	-146.07	-20.91
PBE0+TS (FHI)	-99526.8235	-12439.3414	-1.5115	-145.83	-20.67
optB86b-vdW	-604.5143	-73.9395	-1.6247	-156.76	-31.60
optB88-vdW	-604.3520	-73.9107	-1.6333	-157.58	-32.42
optPBE-vdW	-597.1258	-73.0540	-1.5867	-153.09	-27.93
SCAN	-773.2792	-95.4984	-1.1615	-112.06	13.10
R2SCAN	-758.2517	-93.7145	-1.0670	-102.94	22.22
RSCAN	-791.7860	-97.9312	-1.0420	-100.54	24.62
PBE	-696.8932	-86.3821	-0.7295	-70.38	54.78
revPBE	-678.6959	-84.6688	-0.1682	-16.23	108.93

S2 vdW-DF2 Equations of State

One of the key properties used to train the MLIPs in the main manuscript is the equation of state (EOS) of each molecular crystal in X23. In Fig. S1 we show the EOS for each molecular crystal computed with the vdW-DF2 functional.

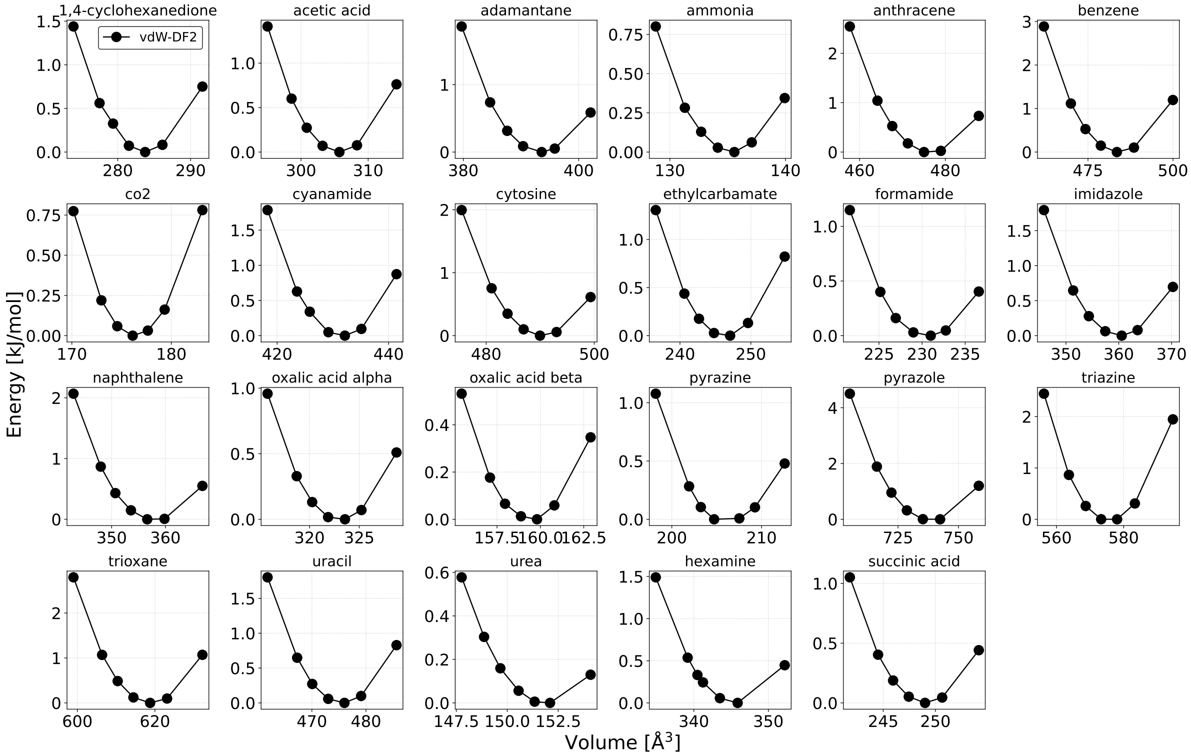

Figure S1: Equations of state of each molecular crystal in the X23 dataset computed with the vdW-DF2 functional.

The EOS are analyzed by fitting the Birch-Murnaghan EOS (see Eq. 1) and obtaining the equilibrium volume V_0, the bulk modulus B_0 and the bulk modulus first derivative with respect to the volume B_0':

$$E(V) = E_0 + \frac{9V_0B_0}{16}\left\{\left[\left(\frac{V_0}{V}\right)^{2/3} - 1\right]^3 B_0' + \left[\left(\frac{V_0}{V}\right)^{2/3} - 1\right]^2 \left[6 - 4\left(\frac{V_0}{V}\right)^{2/3}\right]\right\}, \quad (1)$$

where E is the total energy and V is the volume. In table S25, we report the equilibrium volumes obtained with our fit compared to the experimental values reported in Ref. S2.

These are both experimental volumes measured at finite temperatures (reported as T^* in the table), as well as the zero temperature volume estimated with a thermal expansion correction.[S2] Overall, we find that the vdW-DF2 EOS are reliable for this dataset, with relative errors $< 5-10\%$ on the equilibrium volume compared to experiments.

Table S25: Equilibrium volumes of X23. The table reports the equilibrium volumes according to the Birch-Murnaghan fit (in Å^3) with vdW-DF2, the experimental values at T^*[S2] and the relative error to the finite temperature experimental volumes, the electronic experimental volumes corrected for thermal expansion[S2] (with the error in parentheses) and the correspondent relative errors.

System	vdW-DF2 [Å^3]	T^* [K]	Experiments[S2] at T^* [Å^3]	Percentage Error	Experiments with DFT correction[S2] [Å^3]	Percentage Error
1,4-cyclohexanedione	283.902	133	279.6	1.5	262.5(4.2)	8.2
acetic acid	305.692	40	297.3	2.8	288.8(2.7)	5.8
adamantane	393.488	188	393.1	0.1	357.6(10.6)	10.0
ammonia	135.265	2	128.6	5.2	121.5(1.7)	11.3
anthracene	476.544	16	455.2	4.7	441.2(4.0)	8.0
benzene	483.999	4	461.8	4.8	444.3(7.1)	8.9
carbon dioxide	176.314	6	171.3	2.9	164.8(2.1)	7.0
cyanamide	431.706	108	415.7	3.9	407.9(1.4)	5.8
cytosine	490.352	295	472.4	3.8	440.3(14.3)	11.4
ethylcarbamate	246.369	168	248.8	-1.0	231.2(4.9)	6.6
formamide	230.590	90	224.1	2.9	211.9(4.7)	8.8
imidazole	360.245	123	348.8	3.3	336.4(2.7)	7.1
naphthalene	358.042	10	340.8	5.1	329.7(2.6)	8.6
oxalic acid alpha	323.066	295	312.6	3.3	293.2(6.1)	10.2
oxalic acid beta	159.417	295	156.9	1.6	150.5(1.9)	5.9
pyrazine	206.212	184	203.6	1.3	189.6(4.8)	8.8
pyrazole	738.692	108	698.3	5.8	662.5(11.3)	11.5
triazine	575.588	295	586.8	-1.9	528.0(12.8)	9.0
trioxane	618.961	103	616.5	0.4	580.7(9.6)	6.6
uracil	475.666	295	463.4	2.6	442.0(8.9)	7.6
urea	151.924	12	145.1	4.7	140.8(0.9)	7.9
hexamine	345.790	15	332.4	4.0	321.6(1.6)	7.5
succinic acid	249.092	77	239.3	4.1	233.3(1.5)	6.8

Finally, in Fig. S2 we report the bulk moduli B_0 obtained in our fit. The error bars estimated by changing the number of data points used for the fit by excluding the smallest or largest volume reported in Fig. S1.

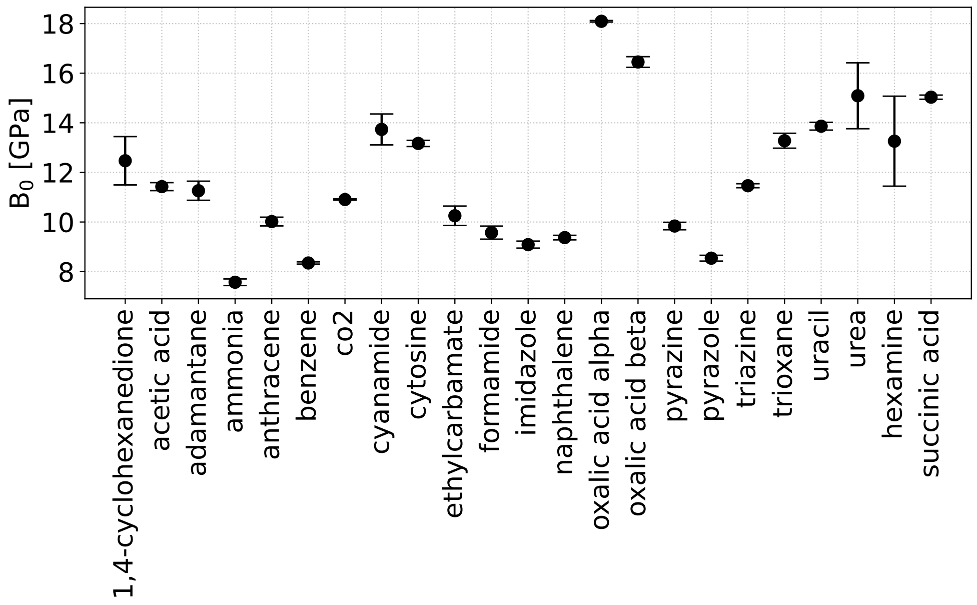

Figure S2: Bulk moduli B_0 of the X23 dataset with the vdW-DF2 functional. The error bars are estimated by changing the number of data points included in the Birch-Murnaghan fit, respectively excluding either the smallest or largest volume reported in Fig. S1

S3 Framework Computational Cost

In this section we report the total cost of the fine tuning of MACE-MP-0b3 for each system in X23. The total cost includes three tasks: (i) the calculations of the reference DFT EOS and vibrational properties (used to test the models); (ii) the calculations of the DFT energy, forces, and stress for each structure in the training set; and (iii) the cost of the fine tuning of MACE-MP-0b3. The cost of each task is reported in Table S26, together with the number of atoms contained in the cell used for the DFT calculations and the number of structures used for fine tuning. Details of the CPU/GPU nodes used for the calculations are given in the caption of Table S26.

Table S26: Analysis of the cost of each step of the framework described in the main manuscript for each system in X23. The table reports the cost of the DFT EOS reference calculations, the DFT calculations of the solid and gas vibrational frequencies, the DFT calculations of energy, forces, and stress for the training set for the solid and gas phase, and the cost of the fine tuning of MACE-MP-0b3. The columns reporting the cost of DFT calculations also contain the number of atoms N_{atoms} in the simulated cell. The columns reporting the training set and fine tuning cost also report the number of structures in the training set.
[a] Cost in CPU node-hours on ARCHER2 (1 CPU node with 128 cores).
[b] Cost in CPU node-hours on CSD3 (1 Ice Lake CPU node with 76 cores).
[c] Cost in GPU-hours on CSD3 (1 NVIDIA A100-SXM-80GB).

System	DFT EOS		DFT Vib Solid		DFT Vib Gas		DFT Training Set Solid			DFT Training Set Gas			MP-0 Fine Tuning	
	N_{atoms}	Cost[a]	N_{atoms}	Cost	N_{atoms}	Cost[a]	N_{atoms}	Structures	Cost[b]	N_{atoms}	Structures	Cost[b]	Structures	Cost[c]
1,4-cyclohexanedione	32	2.2	864	198.1	16	36.5	32	122	2.1	16	64	29.2	186	3.1
acetic acid	32	4.3	768	85.9	8	29.1	32	128	2.5	8	32	13.6	160	2.8
adamantane	52	12.1	208	8.2	26	107.5	52	122	2.9	26	48	21.5	170	4.3
ammonia	16	14.8	1024	56.0	4	9.0	16	120	0.8	4	32	12.7	152	2.2
anthracene	48	46.5	384	38.9	24	55.2	48	122	4.7	24	64	29.1	186	3.7
benzene	48	32.1	384	15.5	12	22.3	48	122	4.0	12	64	26.1	186	3.6
carbon dioxide	12	2.8	324	2.8	3	10.2	12	122	1.4	3	64	25.7	186	2.4
cyanamide	40	86.4	320	11.4	5	19.3	40	164	4.6	5	32	12.1	196	3.5
cytosine	52	48.6	416	43.0	13	67.5	52	122	4.7	13	48	24.6	170	3.6
ethyl carbamate	26	40.0	312	29.7	13	50.3	26	122	2.2	13	64	29.2	186	2.9
formamide	24	36.5	432	20.6	6	15.3	24	117	1.8	6	32	13.5	149	2.3
imidazole	36	47.8	288	18.9	9	33.7	36	128	3.1	9	32	13.0	160	2.9
naphthalene	36	49.5	288	19.3	18	42.3	36	122	3.3	18	32	14.1	154	2.8
oxalic acid alpha	32	35.4	256	8.6	8	11.9	32	164	3.2	8	32	17.0	196	3.2
oxalic acid beta	16	63.3	288	10.6	8	11.7	16	164	2.0	8	32	16.0	196	2.8
pyrazine	20	16.4	240	15.4	10	20.5	20	122	1.4	10	64	27.7	186	2.7
pyrazole	72	79.5	432	78.7	9	33.1	72	119	7.5	9	32	13.9	151	4.4
triazine	54	25.1	324	14.4	9	35.7	54	134	7.3	9	32	15.0	166	3.6
trioxane	72	28.2	576	43.4	12	46.8	72	122	7.6	12	32	14.4	154	4.7
uracil	48	73.2	384	92.8	12	47.5	48	122	6.6	12	32	15.8	154	3.2
urea	16	6.9	256	6.1	8	29.2	16	122	1.3	8	48	21.3	170	2.3
hexamine	22	4.3	176	1.1	22	144.5	22	170	2.0	22	32	15.1	202	3.2
succinic acid	112	283.0	224	11.0	14	80.4	112	122	18.1	14	64	30.6	186	6.8

S4 Fine tuning errors

In this section, we report the training errors of the 23 different fine tuned models for each system in X23. The script used to fine tune each model is provided on GitHub, together with the initial foundation model. The training errors are reported in Table S27. For each system, we show the root mean square error (RMSE) on the energy, forces, and stresses on both the training and validation set. Overall, we achieve low training errors with energies RMSEs that are < 0.6 meV/atom and forces RMSEs that are < 30 meV/Å, with the exception of acetic acid (~ 60 meV/Å) and urea (~ 45 meV/Å).

Table S27: Training and Validation Errors for Energy (meV/atom and kJ/mol), Forces (meV/Å), and Stress (meV/ÅÅ^3) of the 23 fine-tuned models. Energy RMSEs smaller than 10^{-2} meV/atom are reported as 0.00 in the default training script output.

System	Training Set Size	RMSE Energy [meV/atom] (Train – Valid)	RMSE Energy [kJ/mol] (Train – Valid)	RMSE Forces [meV/Å] (Train – Valid)	RMSE Stress [meV/Å Å^3] (Train – Valid)
1,4-cyclohexanedione	186	0.10 – 0.40	0.15 – 0.62	2.20 – 19.90	0.70 – 0.70
acetic acid	160	0.10 – 0.50	0.08 – 0.39	2.10 – 60.30	0.40 – 0.50
adamantane	170	0.10 – 0.10	0.25 – 0.25	2.40 – 6.20	0.50 – 0.50
ammonia	152	0.10 – 0.20	0.04 – 0.08	1.90 – 21.10	0.70 – 0.60
anthracene	186	0.10 – 0.10	0.23 – 0.23	3.00 – 9.70	0.50 – 0.50
benzene	186	0.00 – 0.10	0.00 – 0.12	2.20 – 5.50	0.30 – 0.30
co2	186	0.00 – 0.00	0.00 – 0.00	1.20 – 2.80	0.20 – 0.20
cyanamide	196	0.10 – 0.20	0.05 – 0.10	3.30 – 13.80	0.80 – 0.80
cytosine	170	0.10 – 0.50	0.13 – 0.63	3.10 – 21.20	0.50 – 0.40
ethylcarbamate	186	0.10 – 0.60	0.13 – 0.75	2.40 – 25.30	1.20 – 1.10
formamide	149	0.10 – 0.30	0.06 – 0.17	2.40 – 19.60	0.90 – 0.80
imidazole	160	0.10 – 0.20	0.09 – 0.17	2.70 – 15.50	0.70 – 0.70
naphthalene	154	0.10 – 0.10	0.17 – 0.17	2.60 – 11.20	0.50 – 0.40
oxalic acid alpha	196	0.10 – 0.10	0.08 – 0.08	2.90 – 12.30	1.00 – 1.00
oxalic acid beta	196	0.10 – 0.20	0.08 – 0.15	2.50 – 11.50	1.80 – 1.20
pyrazine	186	0.10 – 0.10	0.10 – 0.10	2.10 – 14.40	0.60 – 0.60
pyrazole	151	0.10 – 0.20	0.09 – 0.17	3.50 – 14.40	0.40 – 0.50
triazine	166	0.10 – 0.40	0.09 – 0.35	3.10 – 16.20	0.60 – 0.40
trioxane	154	0.00 – 0.20	0.00 – 0.23	2.70 – 11.20	0.50 – 0.40
uracil	154	0.10 – 0.60	0.12 – 0.69	2.50 – 29.10	1.00 – 0.90
urea	170	0.10 – 0.50	0.08 – 0.39	1.70 – 44.60	1.30 – 1.20
hexamine	202	0.00 – 0.00	0.00 – 0.00	1.70 – 9.10	0.70 – 0.70
succinic acid	186	0.10 – 0.20	0.14 – 0.27	4.30 – 12.10	0.50 – 0.50

S5 Computational set-up of the MD simulations

In this section, we provide further details on the computational set-up of the classical and path integral MD simulations used to compute the sublimation enthalpies in the main manuscript. In particular, in Table S28, we report for each system the number of molecules in the simulated supercell N_{mol}, the number of atoms in the simulated supercell N_{atoms}, the temperature of the NPT (solid phase) and NVT (gas phase) simulations (in K), and the number of beads N_{beads} of the PIMD simulations.

Table S28: Computational set-up of the MD and PIMD simulations for the calculation of the sublimation enthalpies. The table reports the number of atoms in the solid supercells, the number of molecules in the solid supercells, the temperature of the NPT and NVT simulations, and the number of beads in the PIMD simulations.

System	N_{mol}	N_{atoms}	Temperature [K]	N_{beads}
1,4-cyclohexanedione	16	256	298	32
acetic acid	32	256	290	32
adamantane	16	416	298	32
ammonia	32	128	195	32
anthracene	16	384	298	32
benzene	32	384	279	32
carbon dioxide	32	96	207	32
cyanamide	64	320	298	32
cytosine	32	416	298	32
ethyl carbamate	16	208	298	32
formamide	32	192	276	32
imidazole	32	288	298	32
naphthalene	16	288	298	32
oxalic acid alpha	32	256	298	32
oxalic acid beta	16	128	298	32
pyrazine	16	160	298	32
pyrazole	64	576	298	32
triazine	48	432	298	32
trioxane	48	576	298	32
uracil	32	384	298	32
urea	16	128	298	32
hexamine	8	176	298	32
succinic acid	32	448	298	32

S6 Convergence test: size of the training set

In this section, we report a test on the convergence of the training set data for 1,4-cyclohexanedione. In Fig. S3, we show the convergence of the potential energy contribution to the sublimation enthalpy in the MD approach. As shown in the main manuscript, this is the main contribution to the sublimation enthalpy. In particular, we plot the variation of the potential energy contribution, namely $(U_{\text{gas}} - U_{\text{sol}})$ as a function of the training set size. The error bars are due to the statistical sampling in the MD simulations and are estimated with reblocking. The test shows that the MD estimate of the sublimation enthalpy are well converged with respect to the training set size within ~ 0.2 kJ/mol.

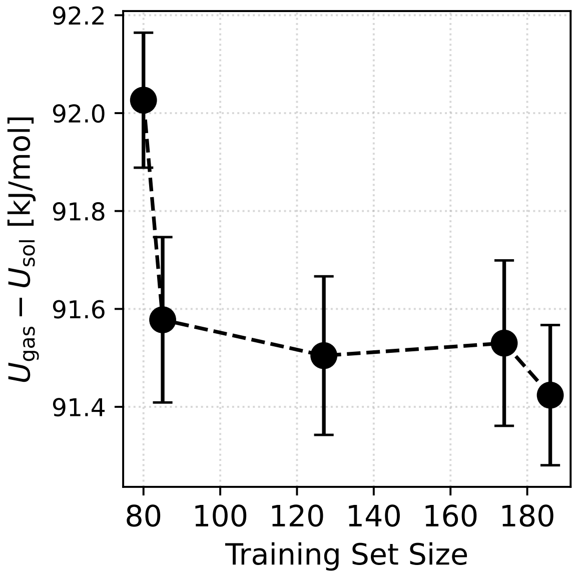

Figure S3: Convergence of the MD sublimation enthalpy with respect to the training set size. The plot shows the potential energy contribution (in kJ/mol) to the MD sublimation enthalpy, i.e. the difference between the potential energy per molecule of the gas and the solid, as a function of the number of structures in the training set.

S7 Convergence test: size of the simulation cell

In this section, we report a test on the convergence of the simulation cell size for 1,4-cyclohexanedione. In Fig. S4, we show the convergence of the potential energy and the volume sampled in the NPT simulations of the solid. In particular, in the left panel we report the variation of the potential energy U per molecule as a function of the number of molecules, measured with respect to the largest tested size (16 molecules, 256 atoms). In the right panel, we report the percentage variation of the volume V per molecule measured with respect to the largest system size.

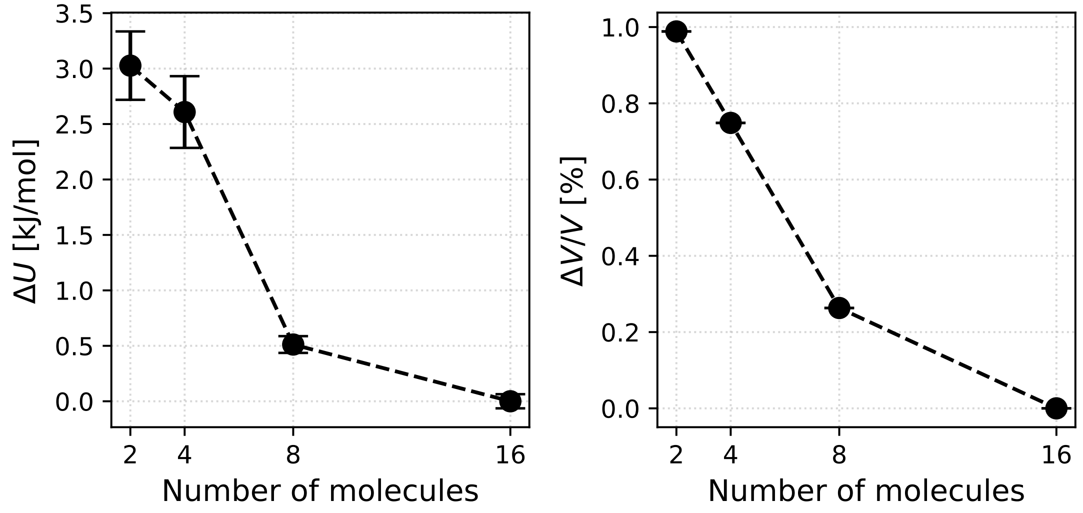

Figure S4: Convergence of the MD simulation set-up with respect to the simulation cell size. (left panel) The plot shows the convergence of the potential energy sampled in the solid NPT simulation, i.e. the potential energy per molecule as a function of the number of molecules in the super cell, measured with respect to the largest tested size ($N_{\mathrm{mol}} = 16$, $N_{\mathrm{atoms}} = 256$). (right panel) The plot shows the convergence of the volume in the solid NPT simulation, i.e. the percentage variation of the volume as a function of the number of molecules, measured with respect to the largest tested size.

S8 Convergence test: number of beads in the PIMD simulations

In this section, we report a test on the convergence of the energy sampled in the PIMD simulations for 1,4-cyclohexanedione with respect to the number of beads. In Fig. S5, we show the convergence of the potential and the (centroid virial) kinetic energy[S3] sampled in the NPT simulations of the solid as a function of the number of beads in the PIMD simulation. The set-up used in the main paper (32 beads) provides converged energies in the PIMD simulations.

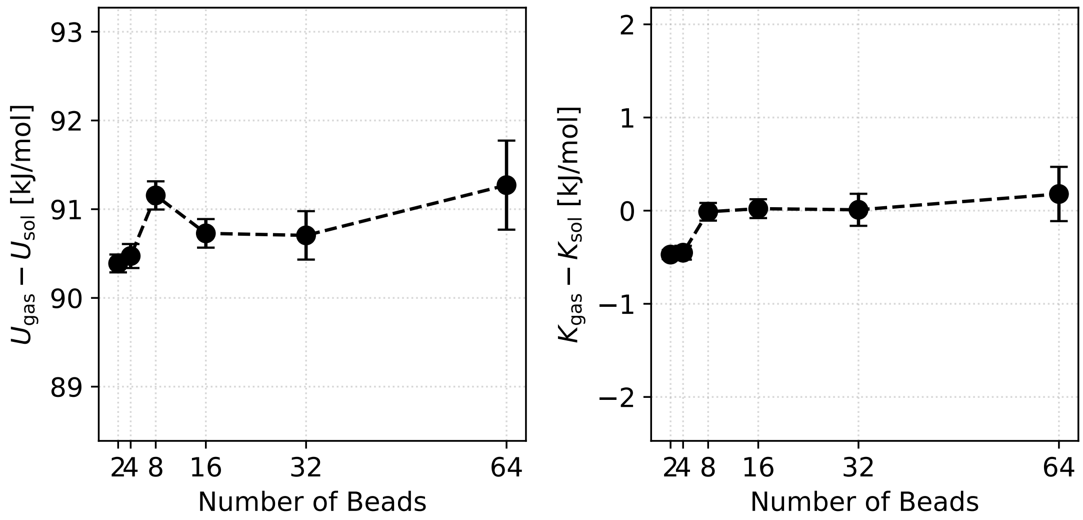

Figure S5: Convergence of the PIMD simulation set-up with respect to the number of beads. (left panel) The plot shows the convergence of the potential energy per molecule (left) and centroid virial kinetic energy per molecule (right) sampled in the solid NPT simulation as a function of the number of beads in the PIMD simulations.

S9 Sublimation enthalpies

In this section, we provide numerical details on the sublimation enthalpies computed with the three different approximations in the main manuscript, i.e. the QHA, the inclusion of the anharmonicity with a classical description of the nuclei (MD), and the inclusion of anharmonicity with a quantum description of the nuclei (PIMD).

First, in Table S29 we report the values of the sublimation enthalpies computed with the three approximations for each system in X23 and shown in the main manuscript. The sublimation enthalpies are computed at the temperature T^* for which experimental estimates of the sublimation enthalpies are available. The temperature T^* is room temperature for all the molecular crystals except: acetic acid ($T^* = 290$ K), ammonia ($T^* = 195$ K), benzene ($T^* = 279$ K), carbon dioxide ($T^* = 207$ K) and formamide ($T^* = 276$ K).

Table S29: Sublimation enthalpies computed with the fine tuned MLIPs, respectively with the QHA, the MD, and the PIMD approach. The sublimation enthalpies are given in kJ/mol.

System	$\Delta H_{\mathrm{sub}}^{\mathrm{QHA}}$	$\Delta H_{\mathrm{sub}}^{\mathrm{MD}}$	$\Delta H_{\mathrm{sub}}^{\mathrm{PIMD}}$
1,4-cyclohexanedione	81.794	82.808	81.932
acetic acid	66.293	66.512	66.747
adamantane	55.627	52.065	54.345
ammonia	32.018	34.572	32.412
anthracene	93.820	96.310	94.649
benzene	44.777	42.930	42.086
carbon dioxide	25.646	26.088	25.589
cyanamide	78.927	78.506	79.375
cytosine	148.415	151.895	150.956
ethyl carbamate	77.749	77.724	78.005
formamide	73.319	76.657	74.475
imidazole	82.381	83.809	83.583
naphthalene	69.890	72.317	70.206
oxalic acid alpha	98.500	96.854	100.115
oxalic acid beta	97.912	97.542	101.299
pyrazine	54.792	55.031	54.182
pyrazole	71.395	72.302	73.268
triazine	56.001	54.244	53.882
trioxane	55.153	56.810	54.320
uracil	127.431	130.764	129.780
urea	102.346	104.346	103.369
hexamine	78.878	82.019	79.466
succinic acid	119.510	127.306	130.356

Now, we provide a breakdown of each contribution to the sublimation enthalpies in the three considered approximations. We start with the sublimation enthalpy computed with the QHA. As described in the **Methods** section of the main manuscript, this is computed as:

$$\Delta H_{\mathrm{sub}}^{\mathrm{QHA}} = E_{\mathrm{gas}}^{\mathrm{el,DMC}} - E_{\mathrm{sol}}^{\mathrm{el,DMC}} + E_{\mathrm{gas}}^{\mathrm{vib,MLIP}} - E_{\mathrm{sol}}^{\mathrm{vib,MLIP}} + 4RT, \tag{2}$$

except for carbon dioxide, where the RT contribution is $(7/2)RT$. The kinetic energy contribution to the QHA sublimation enthalpy is:

$$\Delta K_{\mathrm{sub}}^{\mathrm{QHA}} = \frac{1}{2}\left(E_{\mathrm{gas}}^{\mathrm{vib,MLIP}} - E_{\mathrm{sol}}^{\mathrm{vib,MLIP}}\right) + 3RT, \tag{3}$$

except for carbon dioxide, where the RT contribution is $(5/2)RT$. The contribution of the potential energy is:

$$\Delta U_{\text{sub}}^{\text{QHA}} = \left(E_{\text{gas}}^{\text{el,DMC}} - E_{\text{sol}}^{\text{el,DMC}}\right) + \frac{1}{2}\left(E_{\text{gas}}^{\text{vib,MLIP}} - E_{\text{sol}}^{\text{vib,MLIP}}\right). \quad (4)$$

In Table S30, we report each contribution to the sublimation enthalpy computed with the QHA.

Table S30: Contributions to the QHA sublimation enthalpies. For each system, we report the lattice energy computed with DMC in Ref. S4, the vibrational energies of the solid and the gas, and the RT contribution of the gas (which is equal to 4RT for all systems except carbon dioxide, for which it is $7/2RT$), and the sublimation enthalpy. Each contribution is in kJ/mol.
[a] For carbon dioxide the RT contribution is $(7/2)RT$.

System	$E_{\text{sol}}^{\text{el,DMC}} - E_{\text{gas}}^{\text{el,DMC}}$	$E_{\text{sol}}^{\text{vib,MLIP}}$	$E_{\text{gas}}^{\text{vib,MLIP}}$	4RT	$\Delta H_{\text{sub}}^{\text{QHA}}$
1,4-cyclohexanedione	-88.333	362.263	345.813	9.911	81.794
acetic acid	-71.709	176.179	161.118	9.645	66.293
adamantane	-61.016	648.659	633.359	9.911	55.627
ammonia	-38.198	100.326	87.660	6.485	32.018
anthracene	-100.216	529.956	513.649	9.911	93.820
benzene	-49.789	275.437	261.145	9.279	44.777
carbon dioxide[a]	-29.368	38.154	29.269	5.163	25.646
cyanamide	-83.599	103.257	88.675	9.911	78.927
cytosine	-156.220	277.660	259.944	9.911	148.415
ethyl carbamate	-84.237	301.631	285.231	9.911	77.749
formamide	-80.952	133.776	116.963	9.179	73.319
imidazole	-88.203	199.020	183.287	9.911	82.381
naphthalene	-75.496	403.556	388.039	9.911	69.890
oxalic acid alpha	-102.630	144.133	130.092	9.911	98.500
oxalic acid beta	-102.299	144.398	130.101	9.911	97.912
pyrazine	-61.074	215.316	199.123	9.911	54.792
pyrazole	-77.320	199.233	183.397	9.911	71.395
triazine	-60.479	183.514	169.125	9.911	56.001
trioxane	-62.136	273.156	256.262	9.911	55.153
uracil	-134.267	245.959	229.212	9.911	127.431
urea	-108.534	181.961	165.862	9.911	102.346
hexamine	-86.186	530.118	512.898	9.911	78.878
succinic acid	-125.160	298.269	282.708	9.911	119.510

Now we consider the sublimation enthalpies computed with the MD approach. As de-

scribed in the **Methods** section of the main manuscript, this is computed as:

$$\Delta H_{\rm sub}^{\rm MD} = \left(E_{\rm gas}^{\rm el,DMC} - E_{\rm sol}^{\rm el,DMC}\right) - \left(E_{\rm gas}^{\rm el,MLIP} - E_{\rm sol}^{\rm el,MLIP}\right) + \langle K+U\rangle_{\rm gas} - \langle K+U\rangle_{\rm sol} + \frac{3}{2}RT + RT - p\langle V\rangle_{\rm sol}, \tag{5}$$

where E is the total energy at zero temperature, K is the kinetic energy and U is the potential energy. The kinetic energy contribution to the MD sublimation enthalpy is:

$$\Delta K_{\rm sub}^{\rm MD} = \langle K\rangle_{\rm gas} - \langle K\rangle_{\rm sol} + \frac{3}{2}RT. \tag{6}$$

The contribution of the potential energy is:

$$\Delta U_{\rm sub}^{\rm MD} = \left(E_{\rm gas}^{\rm el,DMC} - E_{\rm sol}^{\rm el,DMC}\right) - \left(E_{\rm gas}^{\rm el,MLIP} - E_{\rm sol}^{\rm el,MLIP}\right) + \langle U\rangle_{\rm gas} - \langle U\rangle_{\rm sol}. \tag{7}$$

In Table S31, we report each contribution to the sublimation enthalpy computed with the MD approach.

Finally, we consider the sublimation enthalpies computed with the PIMD approach. As described in the **Methods** section of the main manuscript, this is computed as:

$$\Delta H_{\rm sub}^{\rm PIMD} = \left(E_{\rm gas}^{\rm el,DMC} - E_{\rm sol}^{\rm el,DMC}\right) - \left(E_{\rm gas}^{\rm el,MLIP} - E_{\rm sol}^{\rm el,MLIP}\right) + \langle K+U\rangle_{\rm gas} - \langle K+U\rangle_{\rm sol} + RT - p\langle V\rangle_{\rm sol}, \tag{8}$$

where E is the total energy at zero temperature, K is the centroid virial estimator of the kinetic energy, and U is the potential energy. The kinetic energy contribution to the PIMD sublimation enthalpy is:

$$\Delta K_{\rm sub}^{\rm PIMD} = \langle K\rangle_{\rm gas} - \langle K\rangle_{\rm sol}, \tag{9}$$

while the contribution of the potential energy is:

$$\Delta U_{\rm sub}^{\rm PIMD} = \left(E_{\rm gas}^{\rm el,DMC} - E_{\rm sol}^{\rm el,DMC}\right) - \left(E_{\rm gas}^{\rm el,MLIP} - E_{\rm sol}^{\rm el,MLIP}\right) + \langle U\rangle_{\rm gas} - \langle U\rangle_{\rm sol}. \tag{10}$$

Table S31: Contributions to the MD sublimation enthalpies. For each system, we report the lattice energy computed with the MLIP and DMC,[S4] the sampled kinetic and potential energies of the solid and the gas, the pressure-volume term for the solid, the RT contribution of the gas (which is equal to (5/2)RT for all systems), and the sublimation enthalpy. Each term is in kJ/mol.

System	$E_{\text{sol}}^{\text{el,MLIP}} - E_{\text{gas}}^{\text{el,MLIP}}$	$E_{\text{sol}}^{\text{el,DMC}} - E_{\text{gas}}^{\text{el,DMC}}$	$\langle K \rangle_{\text{sol}}$	$\langle U \rangle_{\text{sol}}$	$\langle K \rangle_{\text{gas}}$	$\langle U \rangle_{\text{gas}}$	$p\langle V \rangle_{\text{sol}}$	(5/2)RT	$\Delta H_{\text{sub}}^{\text{MD}}$
1,4-cyclohexanedione	-99.589	-88.333	59.252	-8290.877	55.701	-8199.448	0.009	6.194	82.808
acetic acid	-73.333	-71.709	28.860	-3988.106	25.393	-3922.527	0.005	6.028	66.512
adamantane	-82.043	-61.016	96.487	-13282.292	93.246	-13212.141	0.013	6.194	52.065
ammonia	-39.707	-38.198	9.644	-1823.125	7.418	-1788.869	0.002	4.053	34.572
anthracene	-103.896	-100.216	88.856	-13827.214	85.456	-13730.004	0.015	6.194	96.310
benzene	-54.407	-49.789	41.685	-6586.938	38.027	-6541.524	0.008	5.799	42.930
carbon dioxide	-32.770	-29.368	7.689	-1745.498	5.264	-1717.882	0.003	4.303	26.088
cyanamide	-86.601	-83.599	18.514	-2720.246	14.908	-2641.323	0.003	6.194	78.506
cytosine	-152.116	-156.220	48.218	-7207.186	44.577	-7061.941	0.008	6.194	151.895
ethyl carbamate	-90.793	-84.237	48.132	-6487.021	44.366	-6405.162	0.008	6.194	77.724
formamide	-79.535	-80.952	20.547	-3036.538	17.537	-2964.022	0.004	5.737	76.657
imidazole	-86.916	-88.203	33.338	-4904.979	29.846	-4825.153	0.006	6.194	83.809
naphthalene	-77.876	-75.496	66.482	-10212.934	63.672	-10141.609	0.012	6.194	72.317
oxalic acid alpha	-104.012	-102.630	29.701	-4146.662	25.725	-4050.639	0.005	6.194	96.854
oxalic acid beta	-102.624	-102.299	29.530	-4146.215	25.589	-4050.597	0.005	6.194	97.542
pyrazine	-66.056	-61.074	36.998	-5572.241	33.558	-5514.976	0.006	6.194	55.031
pyrazole	-76.032	-77.320	33.376	-4853.317	29.421	-4784.536	0.006	6.194	72.302
triazine	-64.503	-60.479	33.393	-5089.249	29.552	-5033.328	0.006	6.194	54.244
trioxane	-72.801	-62.136	44.471	-5742.047	41.109	-5677.398	0.007	6.194	56.810
uracil	-133.490	-134.267	44.529	-6689.718	40.904	-6562.294	0.007	6.194	130.764
urea	-103.635	-108.534	29.460	-4097.952	25.886	-4001.122	0.005	6.194	104.346
hexamine	-92.584	-86.186	81.431	-11157.215	78.564	-11072.115	0.011	6.194	82.019
succinic acid	-124.624	-125.160	51.925	-7174.160	47.999	-7049.650	0.008	6.194	127.306

In Table S32, we report each contribution to the sublimation enthalpy computed with the PIMD approach.

Table S32: Contributions to the PIMD sublimation enthalpies. For each system, we report the lattice energy computed with the MLIP and DMC,[S4] the sampled (centroid virial) kinetic and potential energies of the solid and the gas the pressure-volume term for the solid, the RT contribution of the gas (which is equal to RT for all systems), and the sublimation enthalpy. Each contribution is in kJ/mol.

System	$E_{\rm sol}^{\rm el,MLIP} - E_{\rm gas}^{\rm el,MLIP}$	$E_{\rm sol}^{\rm el,DMC} - E_{\rm gas}^{\rm el,DMC}$	$\langle K \rangle_{\rm sol}$	$\langle U \rangle_{\rm sol}$	$\langle K \rangle_{\rm gas}$	$\langle U \rangle_{\rm gas}$	$p\langle V \rangle_{\rm sol}$	RT	$\Delta H_{\rm sub}^{\rm PIMD}$
1,4-cyclohexanedione	-99.589	-88.333	181.840	-8161.495	181.849	-8070.785	0.009	2.478	81.932
acetic acid	-73.333	-71.709	88.417	-3925.137	88.753	-3859.508	0.005	2.411	66.747
adamantane	-82.043	-61.016	326.351	-13042.121	327.441	-12970.304	0.013	2.478	54.345
ammonia	-39.707	-38.198	49.769	-1780.438	49.009	-1747.378	0.002	1.621	32.412
anthracene	-103.896	-100.216	267.412	-13640.736	266.857	-13544.315	0.015	2.478	94.649
benzene	-54.407	-49.789	139.066	-6485.041	138.729	-6440.312	0.008	2.320	42.086
carbon dioxide	-32.770	-29.368	19.071	-1734.058	18.969	-1706.682	0.003	1.721	25.589
cyanamide	-86.601	-83.599	52.492	-2684.266	52.906	-2604.777	0.003	2.478	79.375
cytosine	-152.116	-156.220	139.532	-7110.046	139.568	-6965.701	0.008	2.478	150.956
ethyl carbamate	-90.793	-84.237	152.253	-6376.636	152.139	-6294.431	0.008	2.478	78.005
formamide	-79.535	-80.952	66.922	-2987.056	66.177	-2915.544	0.004	2.295	74.475
imidazole	-86.916	-88.203	100.299	-4834.379	100.329	-4754.585	0.006	2.478	83.583
naphthalene	-77.876	-75.496	203.858	-10069.481	203.561	-9999.064	0.012	2.478	70.206
oxalic acid alpha	-104.012	-102.630	71.895	-4101.876	73.109	-4004.065	0.005	2.478	100.115
oxalic acid beta	-102.624	-102.299	71.103	-4101.448	72.759	-4003.953	0.005	2.478	101.299
pyrazine	-66.056	-61.074	108.344	-5497.679	107.899	-5440.541	0.007	2.478	54.182
pyrazole	-76.032	-77.320	100.255	-4782.646	100.436	-4713.319	0.006	2.478	73.268
triazine	-64.503	-60.479	92.645	-5026.913	92.410	-4971.245	0.006	2.478	53.882
trioxane	-72.801	-62.136	136.327	-5644.531	135.288	-5580.978	0.007	2.478	54.320
uracil	-133.490	-134.267	123.404	-6606.446	123.429	-6479.937	0.007	2.478	129.780
urea	-103.635	-108.534	92.533	-4030.705	92.039	-3934.214	0.005	2.478	103.369
hexamine	-92.584	-86.186	265.749	-10963.078	265.185	-10879.118	0.011	2.478	79.466
succinic acid	-124.624	-125.160	149.559	-7071.214	150.678	-6944.982	0.008	2.478	130.356

S10 Benchmark of the fine tuned models

The MLIPs trained for each molecular crystal in the X23 dataset are finally benchmarked against three properties computed with the reference vdW-DF2 functional. The three benchmark properties are: (i) the lattice energy; (ii) the EOS; and (iii) the vibrations in the QHA.

S10.1 Lattice energy

One of the key property in the analysis of the relative stability of molecular crystals is the lattice energy. This is defined as the difference between the total electronic energy of the solid E_{sol} per molecule and the total electronic energy of the gas E_{gas}:

$$E_{\mathrm{latt}} = \frac{E_{\mathrm{sol}}}{N_{\mathrm{mol}}} - E_{\mathrm{gas}}, \tag{11}$$

where N_{mol} is the number of molecules in the solid simulated cell.

In Fig. S6 we report the benchmark of the fine tuned MLIPs on the lattice energies of X23. In particular, the figure shows the difference between the lattice energy computed with the reference method, i.e. the vdW-DF2 functional, and the fine tuned MLIPs. The reference values are well reproduced by the MLIPs, with a root mean square error (RMSE) of ~ 0.1 kJ/mol.

S10.2 Equation of State

The EOS is the key property used to augment each MLIP training set in the framework followed in the main manuscript. The EOS computed in this work with the reference vdW-DF2 functional and with the fine tuned MLIPs are reported in Fig. S7.

Here, we measure the performance of the final models against the reference EOS computed with vdW-DF2 and reported in Sec. S2. As mentioned in Sec. S2, the Birch-Murnaghan fit of the EOS can be sensitive to the number of data points, especially for determining the bulk modulus B_0 and its derivative with respect to the pressure. For this reason, it usually

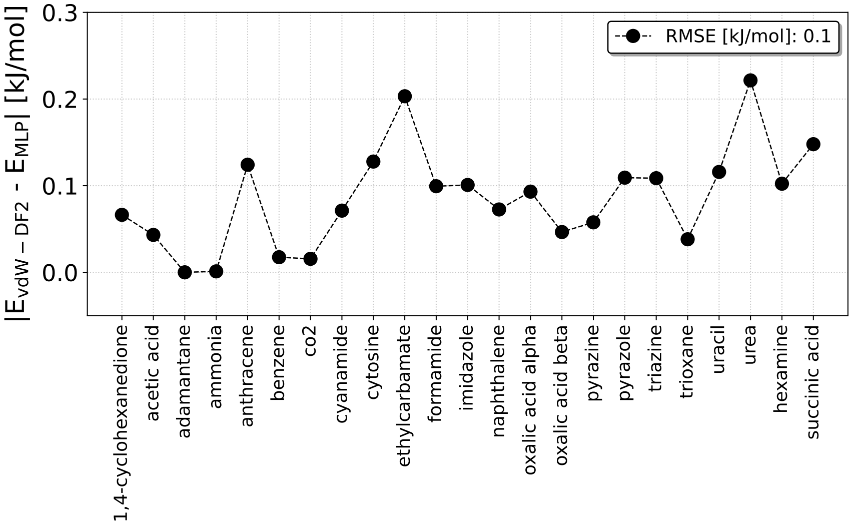

Figure S6: Benchmark of the fine tuned MLIPs on the lattice energy of the X23 dataset. The figure shows the absolute value of the difference between the lattice energy computed with vdW-DF2 and the fine tuned MLIP.

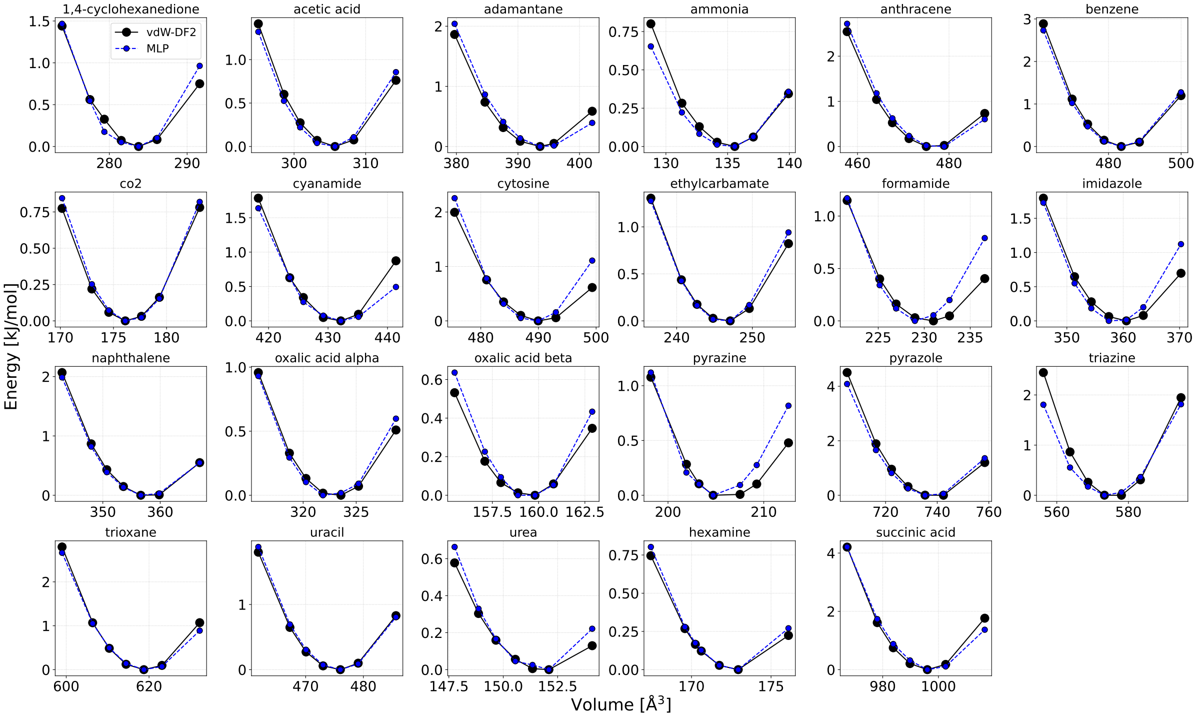

Figure S7: Equations of State of the X23 dataset with the vdW-DF2 functional (black) and the fine tuned MLIPs (blue).

useful to introduce a metric to compare EOS computed with two different computational approaches. One of the metrics typically used is the Δ-metric, introduced by Lejaeghere *et*

al. in Ref. S5. The Δ-metric is defined as:

$$\Delta(a,b) = \sqrt{\frac{1}{V_M - V_m}\int_{V_m}^{V_M} \left[E_a(V) - E_b(V)\right]^2 dV}, \tag{12}$$

where V_M and V_m are the maximum and minimum volume where the EOS is computed, and $E_a(V)$ and $E_b(V)$ are the Birch-Murnaghan fits of the computed datapoints. However, the value of $\Delta(a,b)$, with units of energy, has the shortcoming of being too sensitive to the value of the bulk modulus of the material.[S6]

Two additional metrics were proposed in Ref. S6. Here, we consider the ϵ metric, which is defined as:

$$\epsilon(a,b) = \sqrt{\frac{\langle [E_a(V) - E_b(V)]^2 \rangle}{\sqrt{\langle [E_a(V) - \langle E_a \rangle]^2 \rangle \langle [E_b(V) - \langle E_b \rangle]^2 \rangle}}}, \tag{13}$$

where $\langle E \rangle$ is a shortcut for the integral of the fitted EOS over the volume:

$$\langle E \rangle = \frac{1}{V_M - V_m}\int_{V_m}^{V_M} E(V) dV. \tag{14}$$

The ϵ-metric has been shown to be insensitive to the magnitude of the bulk modulus[S6] and independent of the use of a 'per formula unit' or 'per atom' definition of the EOS.[S6] In addition, it provides a uniform metric across the variety of structural and chemical environments under investigation. The definition of the metric ϵ is grounded in the definition of the coefficient of determination(or R^2) in statistics as a fraction of variance unexplained. We can interpret the value of $1-\epsilon^2$ as the coefficient of determination R^2 in a situation when one EOS $E_a(V)$ (in our case the MLIP EOS) is treated as a fit for the other EOS $E_b(V)$ (in our case the DFT reference EOS). An excellent agreement is defined for $1-\epsilon^2 \sim 0.99$, and a good agreement for $\epsilon \sim 0.95$. In Fig. S8 we report the performance of the fine tuned MLIPs on both the Δ- (top panel) and ϵ-metric (bottom panel). Overall, the fine tuned models achieve a reliable performance, with a RMSE on the Δ metric of ~ 0.1 kJ/mol, comparable with the lattice energy errors, and an average $1-\epsilon^2$ of ~ 0.94.

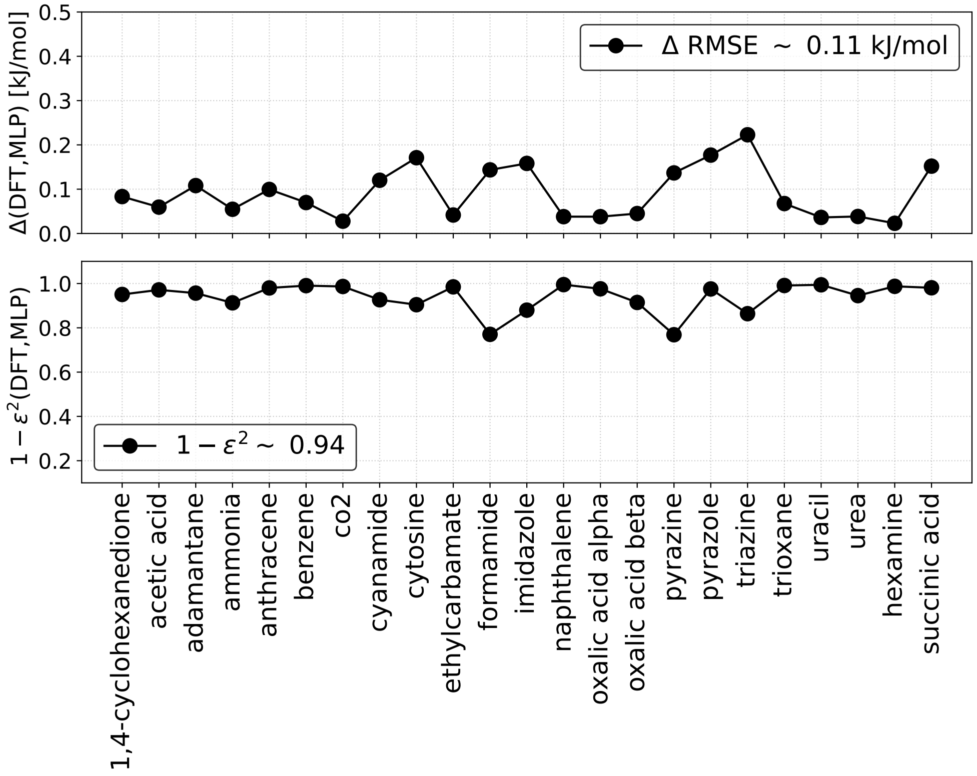

Figure S8: Performance of the fine tuned MLIPs on the EOS. The plot shows the (top panel) Δ- and (bottom panel) ϵ- metric for each system in X23.

S10.3 Quasi-Harmonic vibrations of the solid

The fine tuned models are finally tested on the quasi-harmonic vibrational frequencies of the solid phases. Reproducing correctly the phonon modes is a hard challenge for MLIPs and is an important requirement for the application proposed in the main manuscript, i.e. the analysis of finite temperature stabilities of molecular crystals.

In this section, we report a comparison between the solid quasi-harmonic vibrational properties of the 23 molecular crystals in X23 computed with the reference method (vdW-DF2) and with the fine tuned MLIPs. For each molecular crystal, we report the vibrational density of states in the frequency range $\sim [0, 1000]$ cm^{-1}, the vibrational energy computed with the QHA, and the quasi-harmonic estimation of the constant volume heat capacity C_V as a function of the temperature. For each model, we report two curves for the MLIPs. The blue curves are computed using the same geometry used in the reference DFT calculation ("MLIP" in the legend). The orange curves are computed on the geometries relaxed with the MLIPs ("MLIP(V_{opt})" in the legend). Overall, we find that the fine tuned models reproduce the quasi-harmonic vibrational properties of the solid with < 1 kJ/mol errors compared to the reference DFT functional.

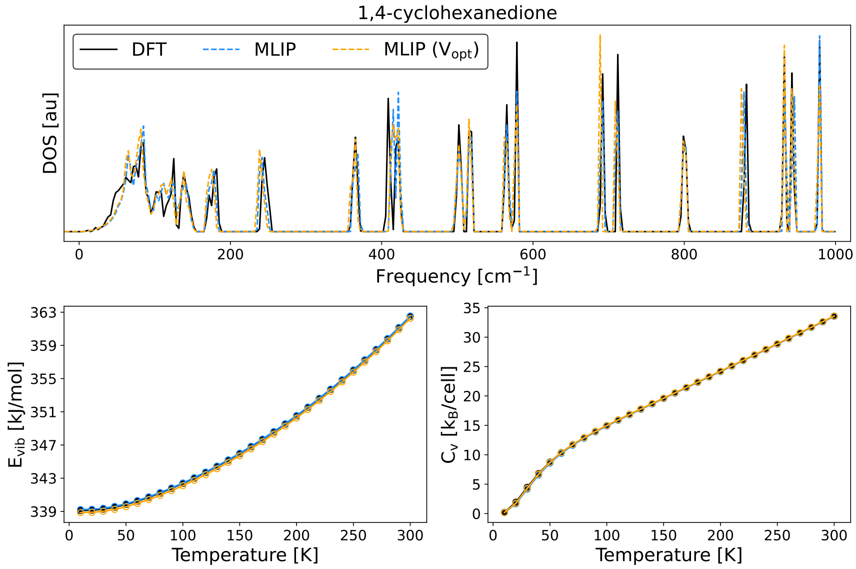

Figure S9: Benchmark of the fine tuned MLIPs on the quasi-harmonic vibrational properties of the solid: 1,4-cyclohexanedione. The plot reports the vibrational density of states (top panel), the vibrational energy (bottom left panel) and the constant volume heat capacity C_V (bottom right panel) computed with vdW-DF2 (black), the fine tuned MLIP on the same geometry as the vdW-DF2 calculation (blue), and the fine tuned MLIP on the relaxed geometry (orange).

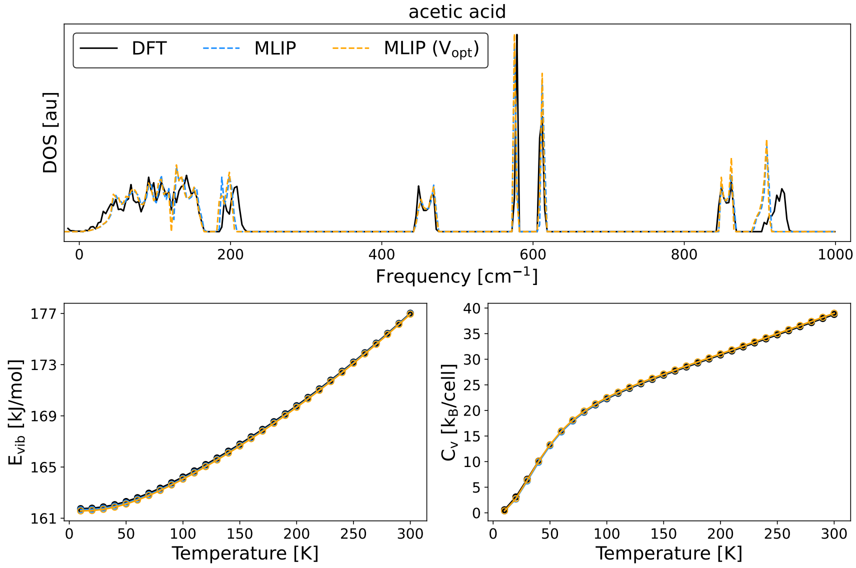

Figure S10: Benchmark of the fine tuned MLIPs on the quasi-harmonic vibrational properties of the solid: acetic acid. The plot reports the vibrational density of states (top panel), the vibrational energy (bottom left panel) and the constant volume heat capacity C_V (bottom right panel) computed with vdW-DF2 (black), the fine tuned MLIP on the same geometry as the vdW-DF2 calculation (blue), and the fine tuned MLIP on the relaxed geometry (orange).

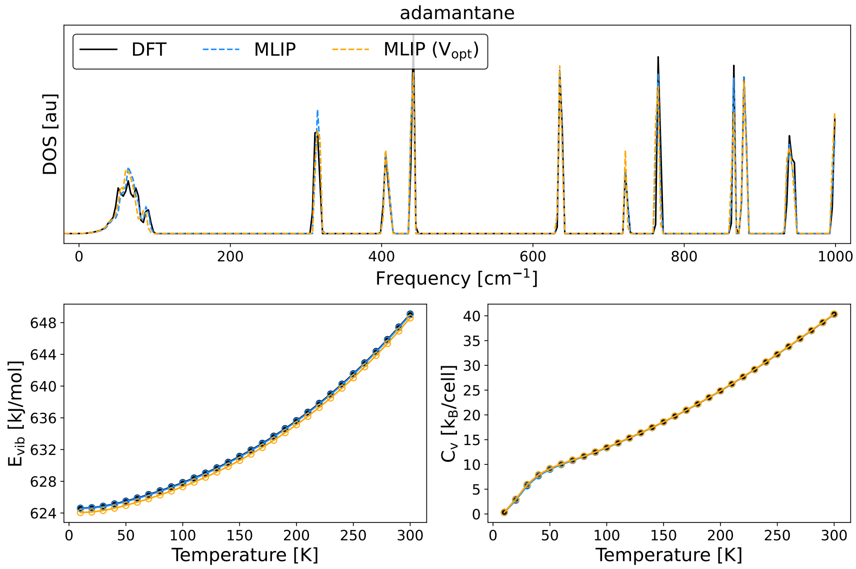

Figure S11: Benchmark of the fine tuned MLIPs on the quasi-harmonic vibrational properties of the solid: adamantane. The plot reports the vibrational density of states (top panel), the vibrational energy (bottom left panel) and the constant volume heat capacity C_V (bottom right panel) computed with vdW-DF2 (black), the fine tuned MLIP on the same geometry as the vdW-DF2 calculation (blue), and the fine tuned MLIP on the relaxed geometry (orange).

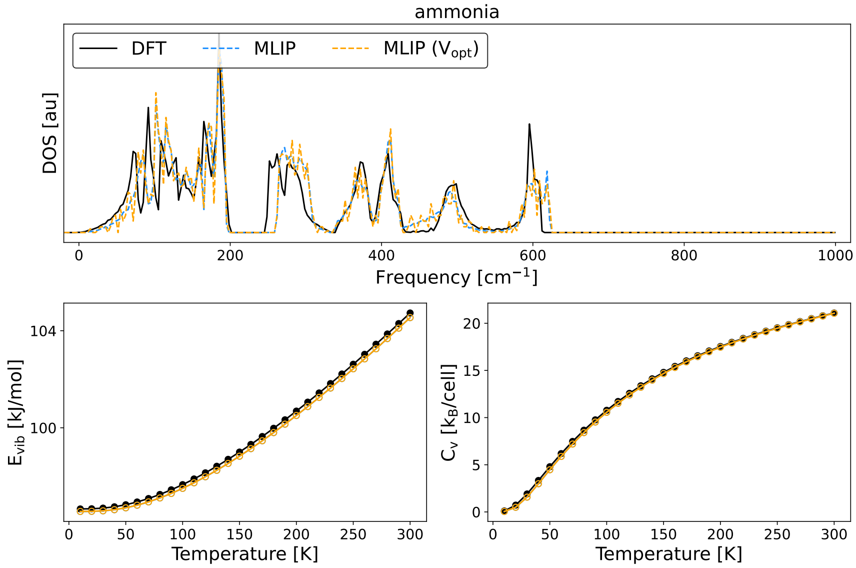

Figure S12: Benchmark of the fine tuned MLIPs on the quasi-harmonic vibrational properties of the solid: ammonia. The plot reports the vibrational density of states (top panel), the vibrational energy (bottom left panel) and the constant volume heat capacity C_V (bottom right panel) computed with vdW-DF2 (black), the fine tuned MLIP on the same geometry as the vdW-DF2 calculation (blue), and the fine tuned MLIP on the relaxed geometry (orange).

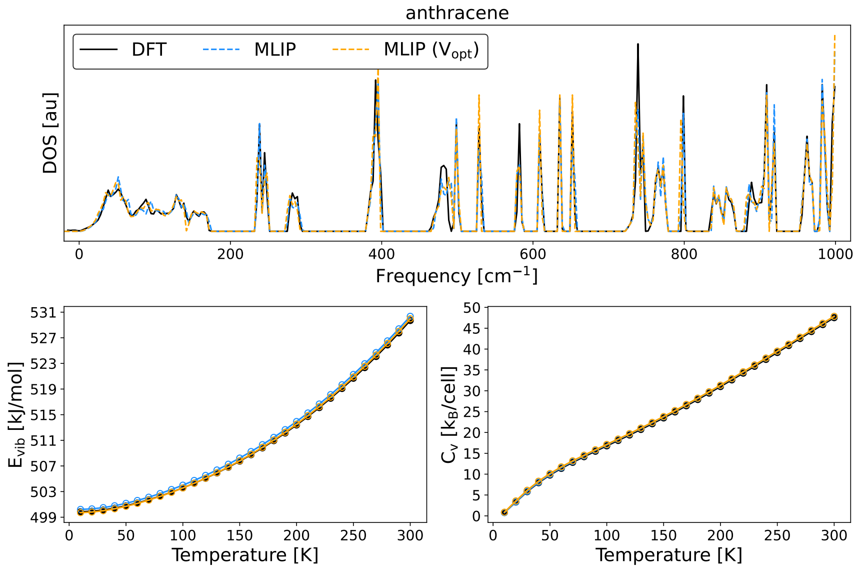

Figure S13: Benchmark of the fine tuned MLIPs on the quasi-harmonic vibrational properties of the solid: anthracene. The plot reports the vibrational density of states (top panel), the vibrational energy (bottom left panel) and the constant volume heat capacity C_V (bottom right panel) computed with vdW-DF2 (black), the fine tuned MLIP on the same geometry as the vdW-DF2 calculation (blue), and the fine tuned MLIP on the relaxed geometry (orange).

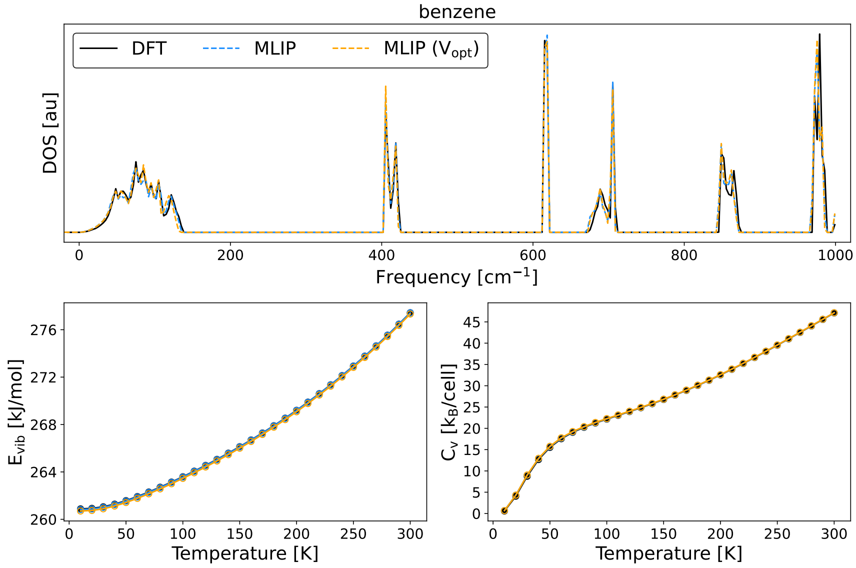

Figure S14: Benchmark of the fine tuned MLIPs on the quasi-harmonic vibrational properties of the solid: benzene. The plot reports the vibrational density of states (top panel), the vibrational energy (bottom left panel) and the constant volume heat capacity C_V (bottom right panel) computed with vdW-DF2 (black), the fine tuned MLIP on the same geometry as the vdW-DF2 calculation (blue), and the fine tuned MLIP on the relaxed geometry (orange).

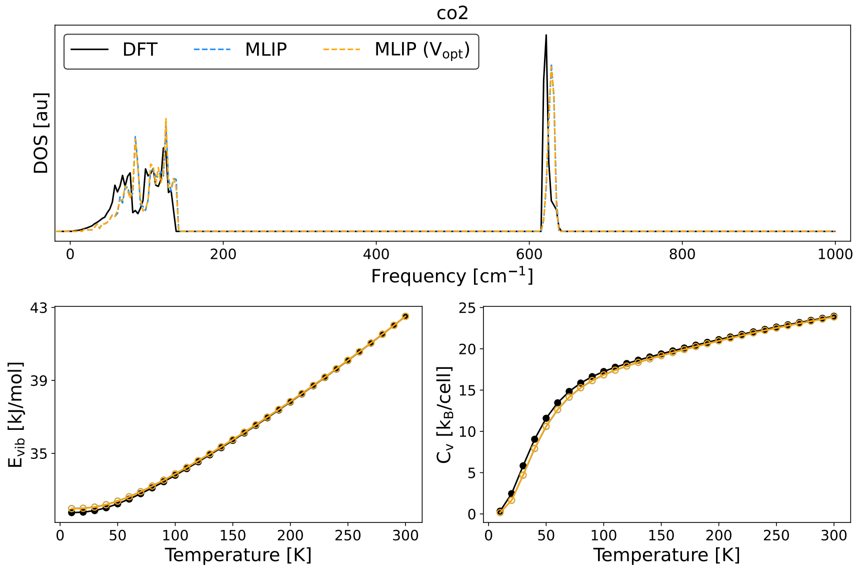

Figure S15: Benchmark of the fine tuned MLIPs on the quasi-harmonic vibrational properties of the solid: carbon dioxide. The plot reports the vibrational density of states (top panel), the vibrational energy (bottom left panel) and the constant volume heat capacity C_V (bottom right panel) computed with vdW-DF2 (black), the fine tuned MLIP on the same geometry as the vdW-DF2 calculation (blue), and the fine tuned MLIP on the relaxed geometry (orange).

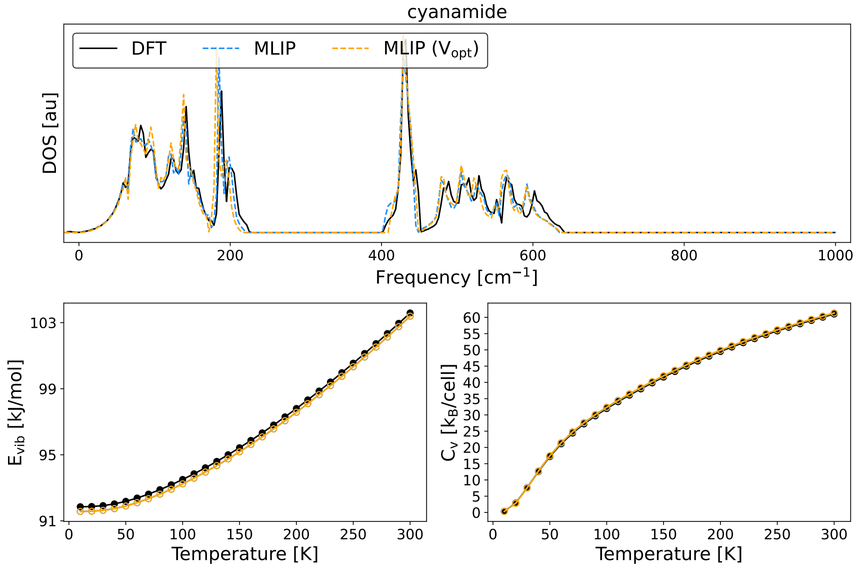

Figure S16: Benchmark of the fine tuned MLIPs on the quasi-harmonic vibrational properties of the solid: cyanamide. The plot reports the vibrational density of states (top panel), the vibrational energy (bottom left panel) and the constant volume heat capacity C_V (bottom right panel) computed with vdW-DF2 (black), the fine tuned MLIP on the same geometry as the vdW-DF2 calculation (blue), and the fine tuned MLIP on the relaxed geometry (orange).

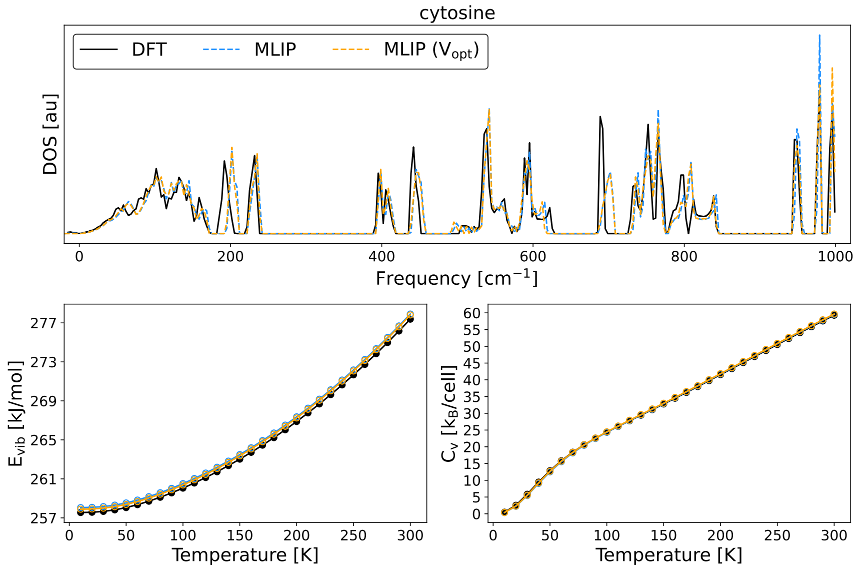

Figure S17: Benchmark of the fine tuned MLIPs on the quasi-harmonic vibrational properties of the solid: cytosine. The plot reports the vibrational density of states (top panel), the vibrational energy (bottom left panel) and the constant volume heat capacity C_V (bottom right panel) computed with vdW-DF2 (black), the fine tuned MLIP on the same geometry as the vdW-DF2 calculation (blue), and the fine tuned MLIP on the relaxed geometry (orange).

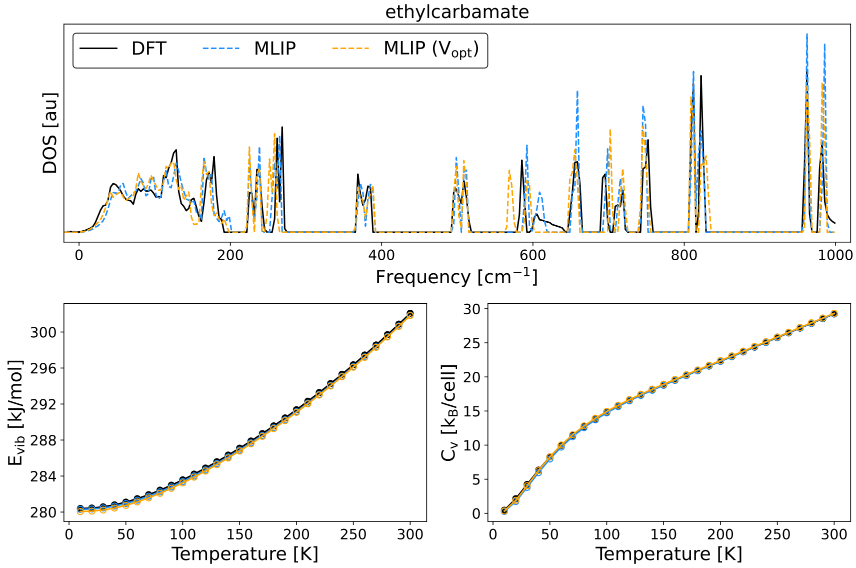

Figure S18: Benchmark of the fine tuned MLIPs on the quasi-harmonic vibrational properties of the solid: ethylcarbamate. The plot reports the vibrational density of states (top panel), the vibrational energy (bottom left panel) and the constant volume heat capacity C_V (bottom right panel) computed with vdW-DF2 (black), the fine tuned MLIP on the same geometry as the vdW-DF2 calculation (blue), and the fine tuned MLIP on the relaxed geometry (orange).

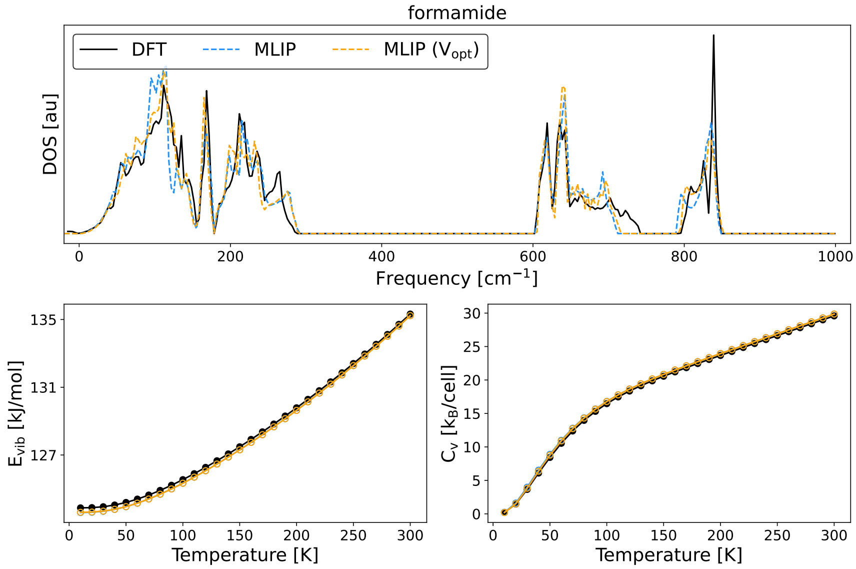

Figure S19: Benchmark of the fine tuned MLIPs on the quasi-harmonic vibrational properties of the solid: formamide. The plot reports the vibrational density of states (top panel), the vibrational energy (bottom left panel) and the constant volume heat capacity C_V (bottom right panel) computed with vdW-DF2 (black), the fine tuned MLIP on the same geometry as the vdW-DF2 calculation (blue), and the fine tuned MLIP on the relaxed geometry (orange).

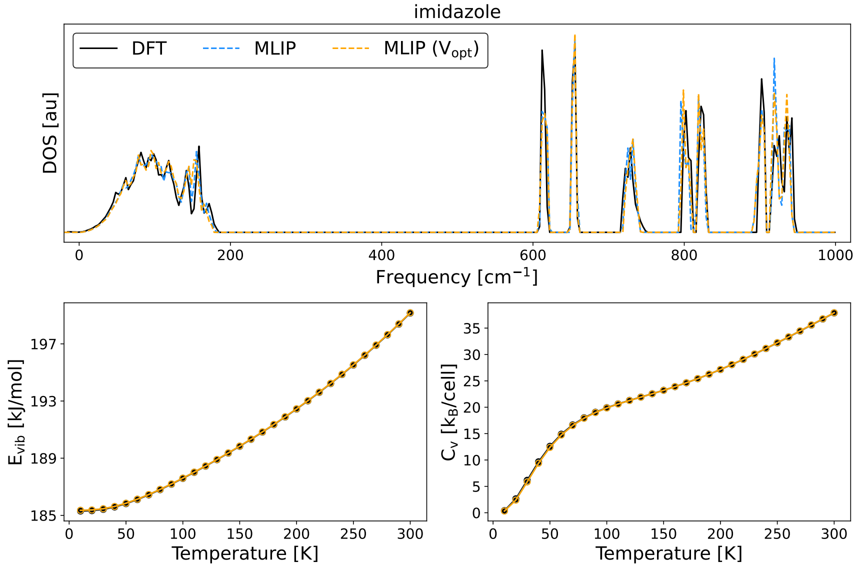

Figure S20: Benchmark of the fine tuned MLIPs on the quasi-harmonic vibrational properties of the solid: imidazole. The plot reports the vibrational density of states (top panel), the vibrational energy (bottom left panel) and the constant volume heat capacity C_V (bottom right panel) computed with vdW-DF2 (black), the fine tuned MLIP on the same geometry as the vdW-DF2 calculation (blue), and the fine tuned MLIP on the relaxed geometry (orange).

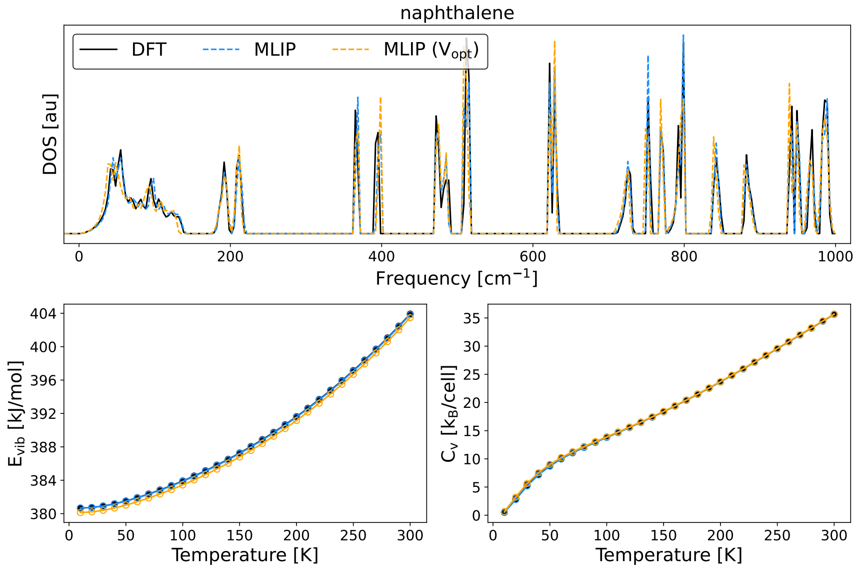

Figure S21: Benchmark of the fine tuned MLIPs on the quasi-harmonic vibrational properties of the solid: naphthalene. The plot reports the vibrational density of states (top panel), the vibrational energy (bottom left panel) and the constant volume heat capacity C_V (bottom right panel) computed with vdW-DF2 (black), the fine tuned MLIP on the same geometry as the vdW-DF2 calculation (blue), and the fine tuned MLIP on the relaxed geometry (orange).

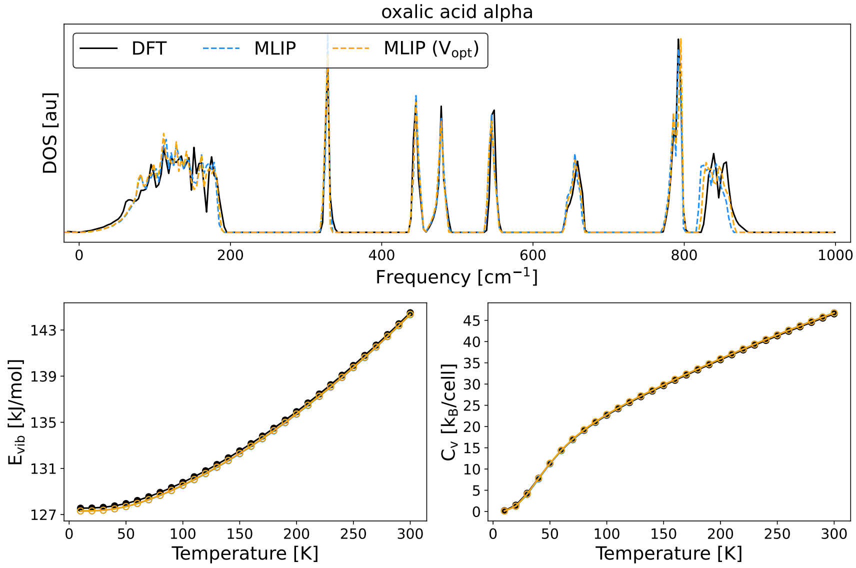

Figure S22: Benchmark of the fine tuned MLIPs on the quasi-harmonic vibrational properties of the solid: oxalic acid α. The plot reports the vibrational density of states (top panel), the vibrational energy (bottom left panel) and the constant volume heat capacity C_V (bottom right panel) computed with vdW-DF2 (black), the fine tuned MLIP on the same geometry as the vdW-DF2 calculation (blue), and the fine tuned MLIP on the relaxed geometry (orange).

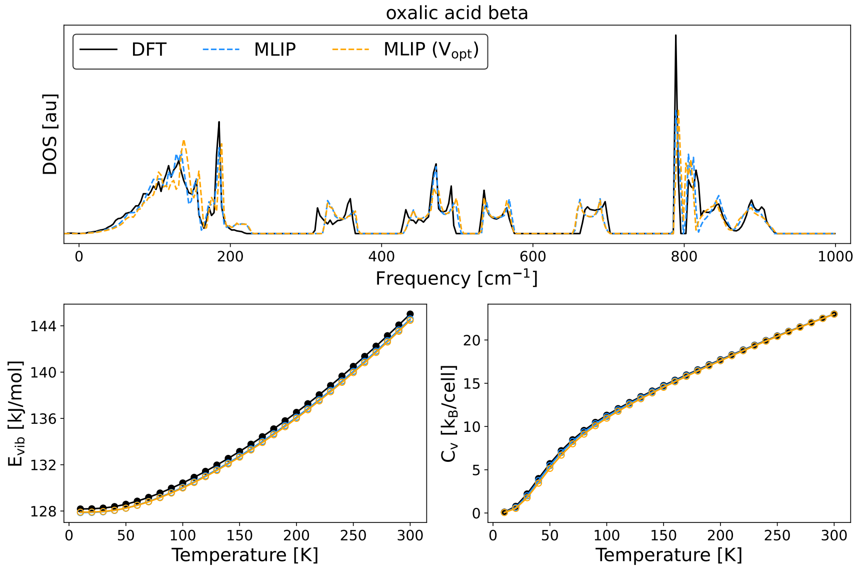

Figure S23: Benchmark of the fine tuned MLIPs on the quasi-harmonic vibrational properties of the solid: oxali acid β. The plot reports the vibrational density of states (top panel), the vibrational energy (bottom left panel) and the constant volume heat capacity C_V (bottom right panel) computed with vdW-DF2 (black), the fine tuned MLIP on the same geometry as the vdW-DF2 calculation (blue), and the fine tuned MLIP on the relaxed geometry (orange).

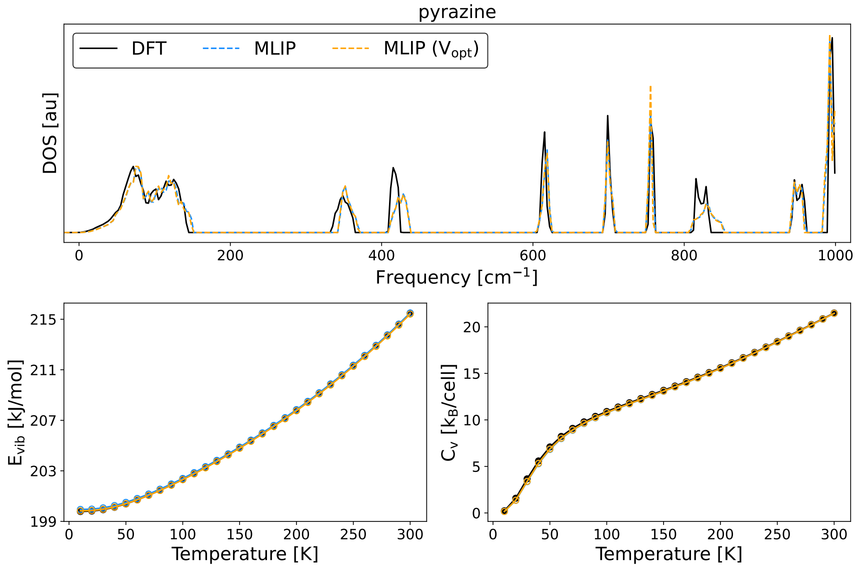

Figure S24: Benchmark of the fine tuned MLIPs on the quasi-harmonic vibrational properties of the solid: pyrazine. The plot reports the vibrational density of states (top panel), the vibrational energy (bottom left panel) and the constant volume heat capacity C_V (bottom right panel) computed with vdW-DF2 (black), the fine tuned MLIP on the same geometry as the vdW-DF2 calculation (blue), and the fine tuned MLIP on the relaxed geometry (orange).

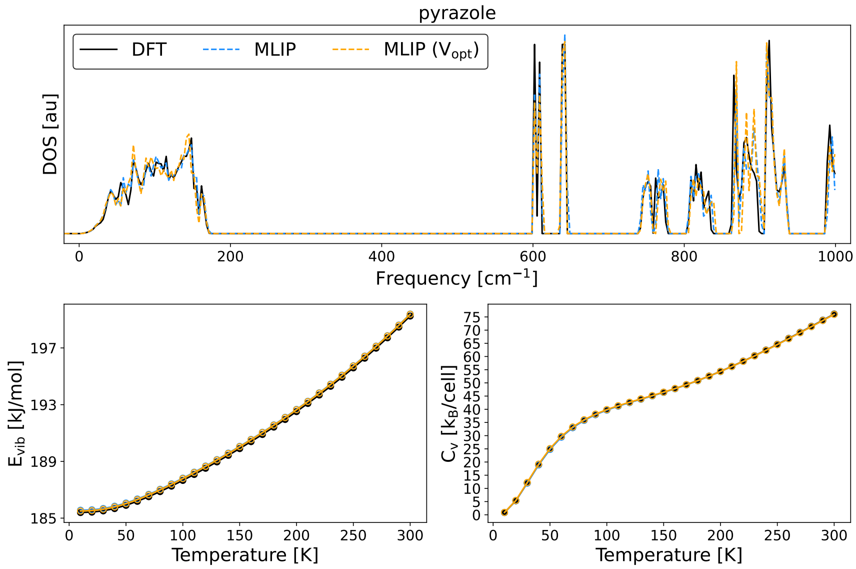

Figure S25: Benchmark of the fine tuned MLIPs on the quasi-harmonic vibrational properties of the solid: pyrazole. The plot reports the vibrational density of states (top panel), the vibrational energy (bottom left panel) and the constant volume heat capacity C_V (bottom right panel) computed with vdW-DF2 (black), the fine tuned MLIP on the same geometry as the vdW-DF2 calculation (blue), and the fine tuned MLIP on the relaxed geometry (orange).

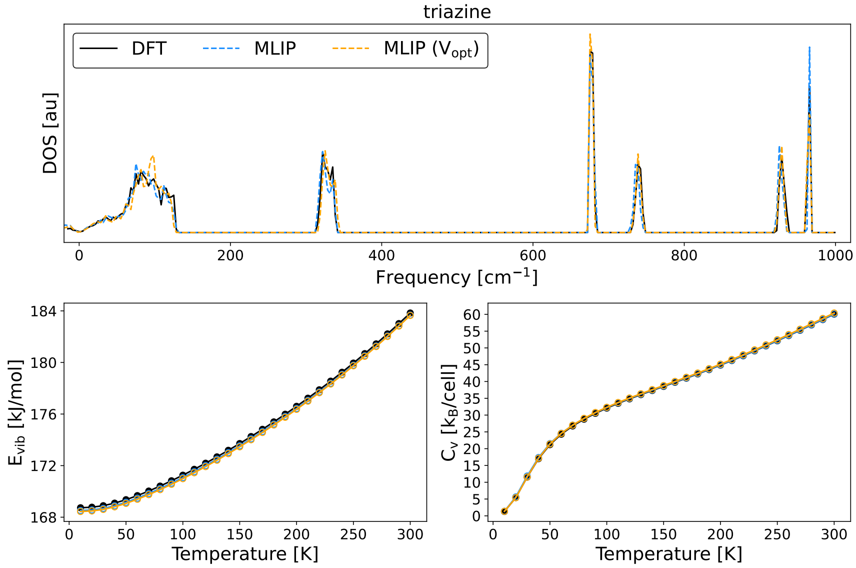

Figure S26: Benchmark of the fine tuned MLIPs on the quasi-harmonic vibrational properties of the solid: triazine. The plot reports the vibrational density of states (top panel), the vibrational energy (bottom left panel) and the constant volume heat capacity C_V (bottom right panel) computed with vdW-DF2 (black), the fine tuned MLIP on the same geometry as the vdW-DF2 calculation (blue), and the fine tuned MLIP on the relaxed geometry (orange).

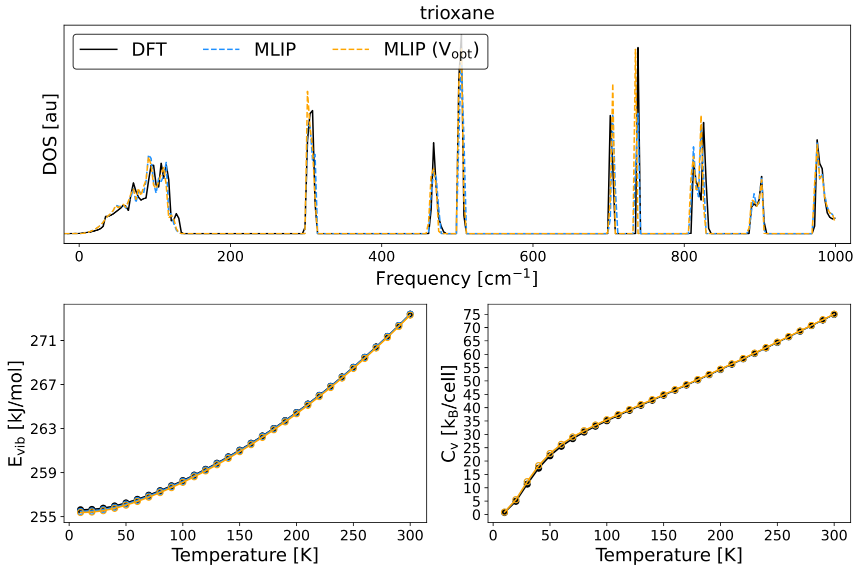

Figure S27: Benchmark of the fine tuned MLIPs on the quasi-harmonic vibrational properties of the solid: trioxane. The plot reports the vibrational density of states (top panel), the vibrational energy (bottom left panel) and the constant volume heat capacity C_V (bottom right panel) computed with vdW-DF2 (black), the fine tuned MLIP on the same geometry as the vdW-DF2 calculation (blue), and the fine tuned MLIP on the relaxed geometry (orange).

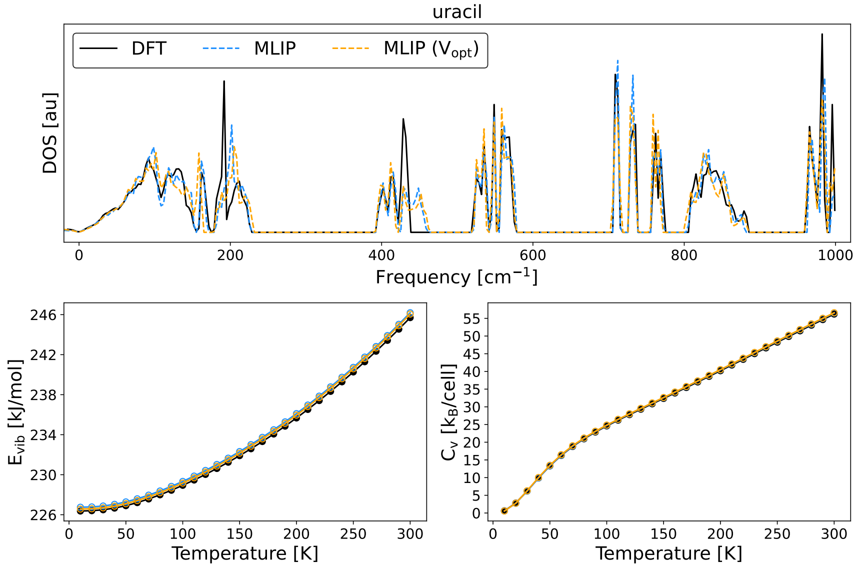

Figure S28: Benchmark of the fine tuned MLIPs on the quasi-harmonic vibrational properties of the solid: uracil. The plot reports the vibrational density of states (top panel), the vibrational energy (bottom left panel) and the constant volume heat capacity C_V (bottom right panel) computed with vdW-DF2 (black), the fine tuned MLIP on the same geometry as the vdW-DF2 calculation (blue), and the fine tuned MLIP on the relaxed geometry (orange).

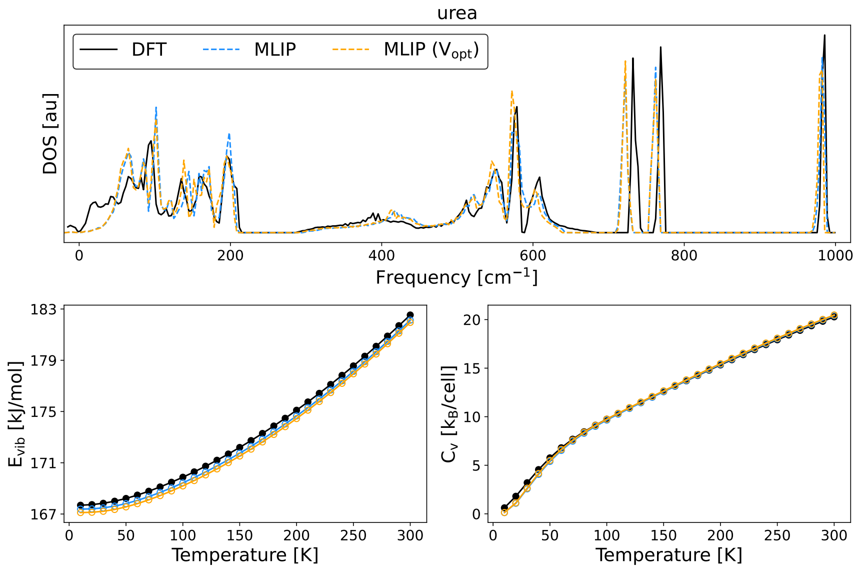

Figure S29: Benchmark of the fine tuned MLIPs on the quasi-harmonic vibrational properties of the solid: urea. The plot reports the vibrational density of states (top panel), the vibrational energy (bottom left panel) and the constant volume heat capacity C_V (bottom right panel) computed with vdW-DF2 (black), the fine tuned MLIP on the same geometry as the vdW-DF2 calculation (blue), and the fine tuned MLIP on the relaxed geometry (orange).

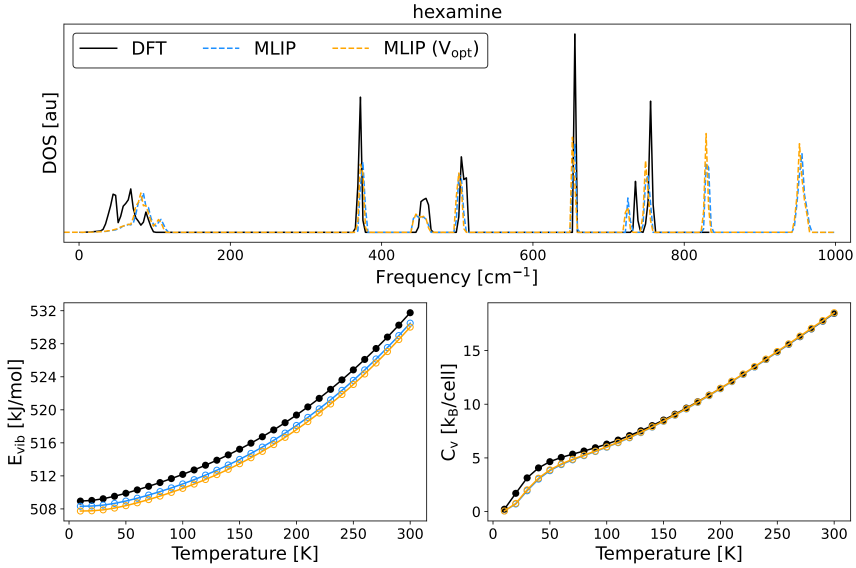

Figure S30: Benchmark of the fine tuned MLIPs on the quasi-harmonic vibrational properties of the solid: hexamine. The plot reports the vibrational density of states (top panel), the vibrational energy (bottom left panel) and the constant volume heat capacity C_V (bottom right panel) computed with vdW-DF2 (black), the fine tuned MLIP on the same geometry as the vdW-DF2 calculation (blue), and the fine tuned MLIP on the relaxed geometry (orange).

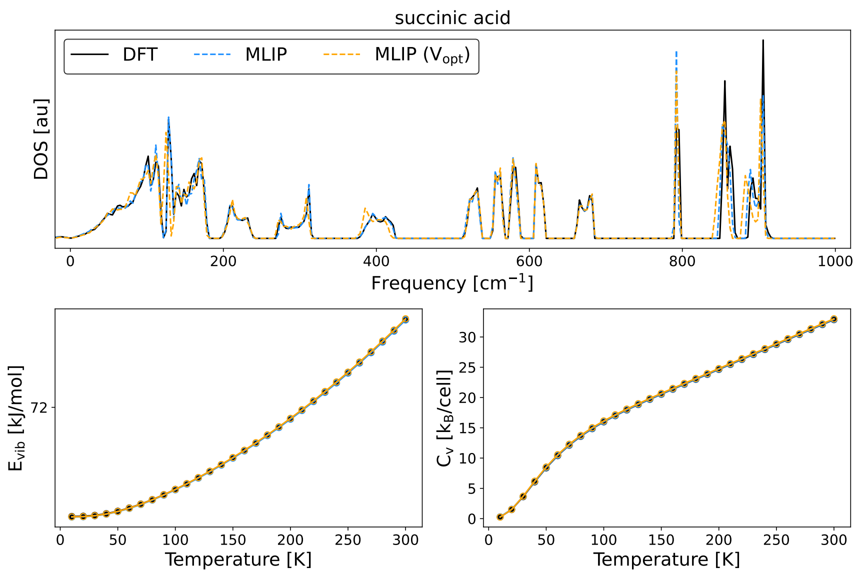

Figure S31: Benchmark of the fine tuned MLIPs on the quasi-harmonic vibrational properties of the solid: succinic acid. The plot reports the vibrational density of states (top panel), the vibrational energy (bottom left panel) and the constant volume heat capacity C_V (bottom right panel) computed with vdW-DF2 (black), the fine tuned MLIP on the same geometry as the vdW-DF2 calculation (blue), and the fine tuned MLIP on the relaxed geometry (orange).

S10.4 QHA vibrational contribution to the sublimation enthalpy

The vibrational contribution to the sublimation enthalpy in the QHA is given by:

$$\Delta E_{\text{vib}}^{\text{QHA}} = E_{\text{gas}}^{\text{vib,QHA}} - E_{\text{sol}}^{\text{vib,QHA}}, \tag{15}$$

where the vibrational energy of the solid and the gas are defined in Eqs. **??** and **??** of the main manuscript. In this section, we report the error on the QHA vibrational contribution to the sublimation enthalpy between vdW-DF2 and the fine tuned MLIP for each molecular crystal in X23. In particular, in Fig. we report the absolute error (in kJ/mol) in the top panel and the percentage error in the bottom panel. Overall, we achieve sub-chemical accuracy (< 4 kJ/mol) on the vibrational contribution, with a RMSE of ~ 0.8 kJ/mol. The errors on $\Delta_{\text{vib}}^{\text{QHA}}$ are comparable with the uncertainty reported in Ref. S2, and as shown in Sec. S11.

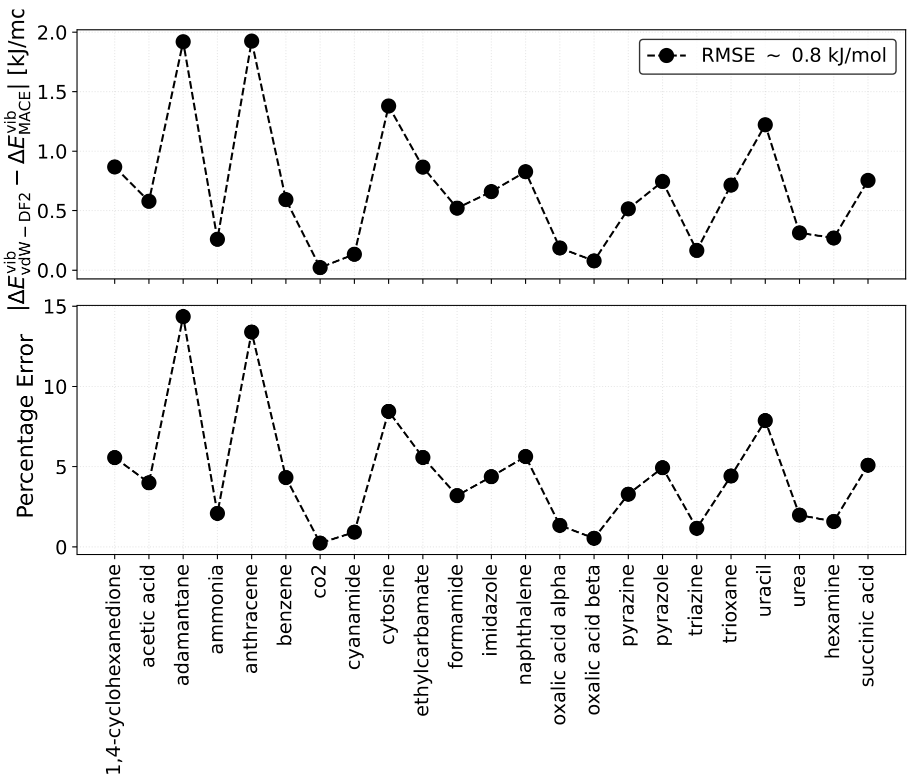

Figure S32: Benchmark of the fine tuned MLIPs on the QHA vibrational contribution to the sublimation enthalpy. The plot shows the absolute error in the top panel and percentage error in the bottom panel for each system in X23.

S11 QHA sublimation enthalpies: comparison with previous work

The X23 finite temperature sublimation enthalpies in the QHA limit have been computed with different approaches and DFT functionals in Refs. S2,S7,S8. In the most recent work,[S2] the QHA finite temperature contribution to the sublimation enthalpy, namely $E_{\rm gas}^{\rm vib} - E_{\rm sol}^{\rm vib} + 4RT$ (except for carbon dioxide where the RT contribution is given by $(7/2)RT$, was computed as an average of three different DFT functionals, i.e. PBE+D3, BLYP+D3, and rPBE+D3.

In Fig. S33, we show a comparison between the finite temperature QHA vibrational contributions computed by Dolgonos *et al.* in Ref. S2 and those computed in this work. The error bars on the MLIP estimates are given by the error with respect to the DFT reference calculations reported in Sec. S10 (see Fig. S32). The error bars in Ref. S2 were estimated from the deviation of the three used functionals (PBE+D3, RPBE+D3, and BLYP+D3). Overall, we observe a good agreement between the two estimates. Slightly larger differences of $\sim 1-2$ kJ/mol, such as those for carbon dioxide or triazine, can be ascribed to differences in the DFT functionals and the optimised geometry.

Overall, this analysis confirms that the choice of the DFT functional plays a larger role in the calculation of the zero temperature contribution to the sublimation enthalpy (i.e. the lattice energy) than in the estimation of the vibrational part. Since the zero temperature contribution in the main manuscript is estimated with DMC,[S1] we conclude that the choice of the vdW-DF2 functional (among those that achieve a reliable performance as reported in Sec. S1) plays a minor role in the final sublimation enthalpies reported in the main manuscript.

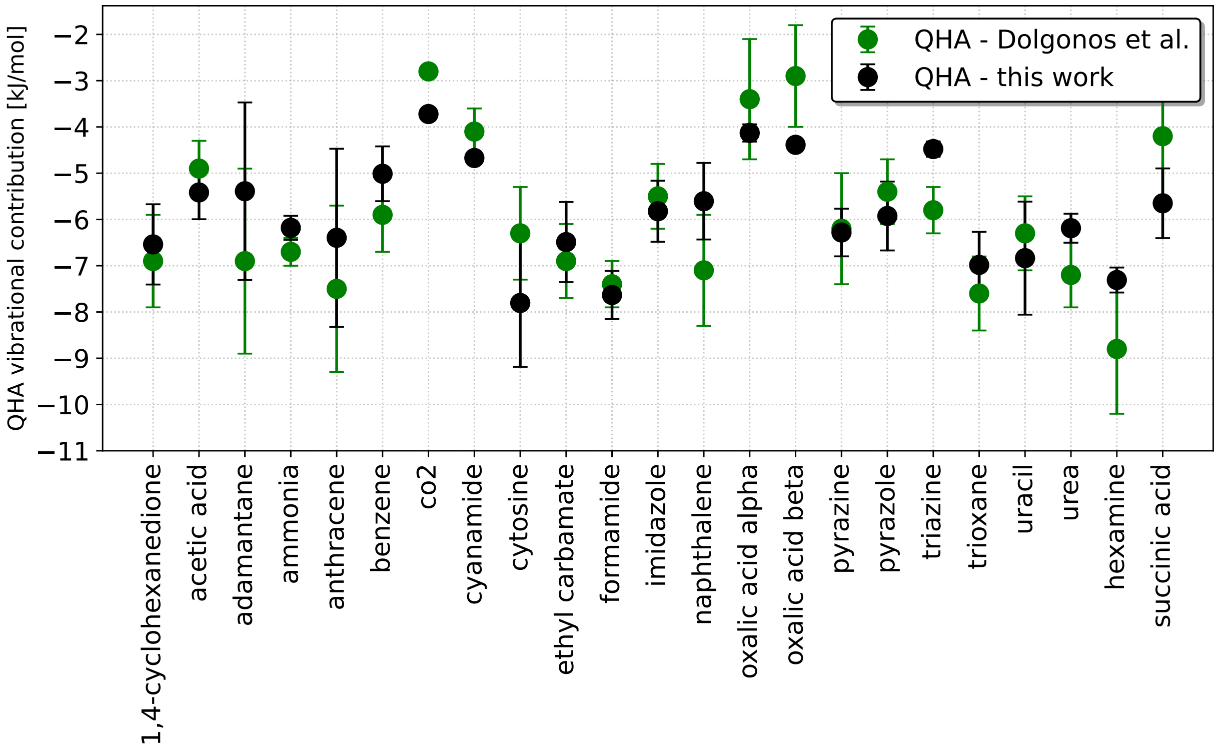

Figure S33: Vibrational contribution to the sublimation enthalpy with the QHA: comparison with previous work. The plot shows the vibrational contribution to the sublimation enthalpy in the QHA computed in this work (black) and in Ref. S2 (green).

S12 Ice polymorphs

In the main manuscript, the framework described to fine tune MACE-MP-0b3 was applied to the 23 crystals of the X23 dataset at the vdW-DF2 level of accuracy. In this section, we show that the framework also work for a different class of molecular crystals, i.e. the ice polymorphs. These tests show that the data efficiency and the accuracy showcased in the main manuscript are not dependent on the choice of the DFT functional or the type of molecular crystals.

The DFT functional used to compute reference energies, forces, and stress for the ice polymorphs is revPBE-D3, which has been showed to reproduce the lattice energies of the ice polymorphs with sub-chemical accuracy against DMC reference values.[S4]

The training set used for the ice polymorphs contains 32 structures for each ice phase of the DMC-ICE13 dataset,[S4] as well as 48 structures of gas phase water clusters (monomer, dimer, trimer, tetramer, pentamer, and hexamer). The training errors for the fine tuned model are reported in Table S33 and are comparable to those obtained for X23 (see Table S27).

Table S33: Training and Validation Errors for Energy (meV/atom), Forces (meV/Å), and Stress (meV/Å^3) of the fine tuned model for the ice polymorphs.

Dataset	RMSE Energy [meV/atom]	RMSE Forces [meV/Å]	RMSE Stress [meV/Å^3]
Training	0.1	3.4	0.5
Validation	0.2	12.6	0.4

The fine tuned model was subsequently tested on the calculation of lattice energies, equations of state, and the vibrational energy (of hexagonal ice).

In Fig. S34 we show the lattice energies of the ice polymorphs computed with the reference DFT functional revPBE-D3 (black) and the fine tuned MACE model (blue). In the left panel, we plot the lattice energies of the 13 ice polymorphs of the DMC-ICE13 dataset, which are included in the training set. In the right panel, we also report the lattice energies of ice IV and XII, which are not included in the training set. The fine tuned model reproduces the

reference lattice energy with a RMSE $\sim$ 0.1 kJ/mol.

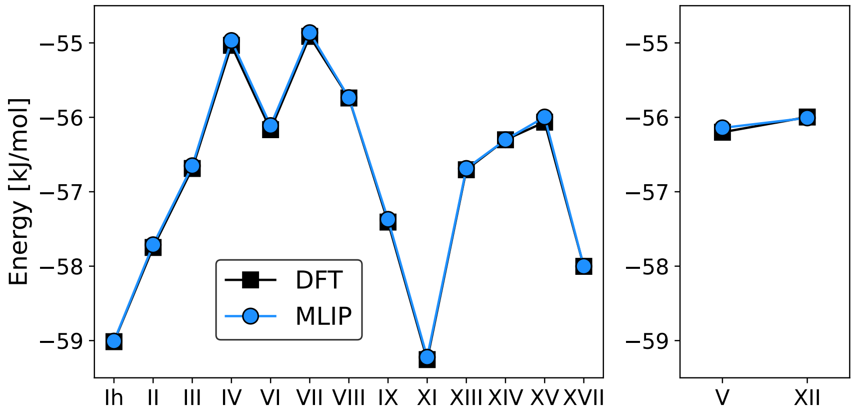

Figure S34: Lattice energies of the ice polymorphs. The figure shows the lattice energy computed with revPBE-D3 (black) and the fine tuned MLIP (blue) for the 13 ice polymorphs of DMC-ICE13[S4] as well as ice IV and XII, which are not included in the training set.

In Fig. S35 we show the EOS of the ice polymorphs computed with the reference DFT functional revPBE-D3 (black) and the fine tuned MLIP (blue). The EOS are correctly reproduced by the fine tuned model, with an energy error as a function of the volume of $\sim$ 0.1 kJ/mol.

In Fig. S36 we show the vibrational energy E_{vib} in the QHA as a function of the temperature, obtained with revPBE-D3 (black) and the fine tuned MACE model (blue). The QHA vibrational energy is reproduced with an error of $<$ 0.1 kJ/mol.

Overall, the reported tests suggest that the framework applied in the main manuscript to X23 allows for a data efficient fine tuning of MACE-MP-0b3 that achieve a sub-chemical accuracy (errors smaller than 1 kJ/mol) description of molecular crystals and is not strongly sensitive to the DFT functional or the type of molecular crystals.

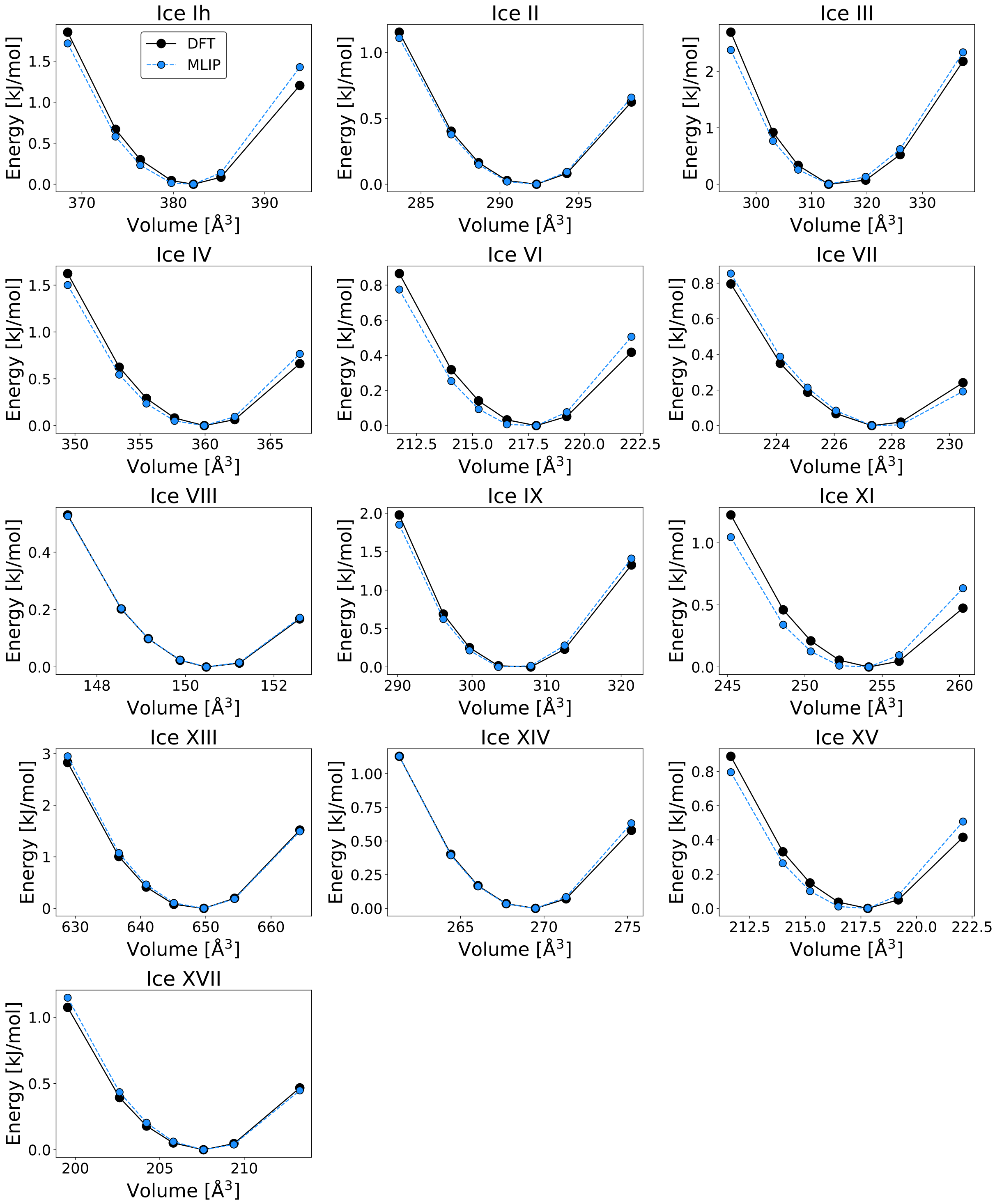

Figure S35: Equations of state of the ice polymorphs. The figure shows the EOS for the 13 ice polymorphs of DMC-ICE13 computed with revPBE-D3 (black) and the fine tuned model (blue).

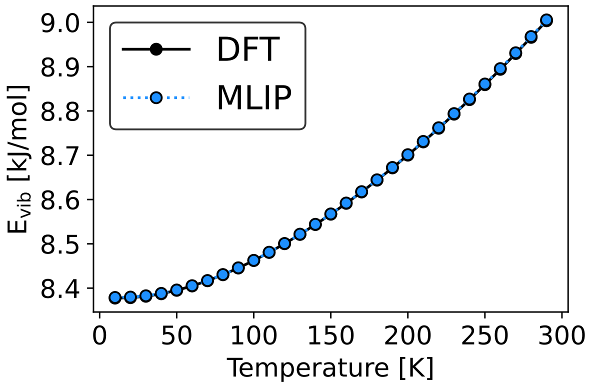

Figure S36: Vibrational energy E_{vib} of hexagonal ice Ih as a function of the temperature computed with revPBE-D3 (black) and the fine tuned model (blue).

S13 General Model vs System Specific Model

The procedure discussed in the main manuscript was used to fine tune 23 individual models, one for each system in the X23 dataset. We will refer to these models as "system specific" models. In this section, we will now show a comparison of the performance between the system specific models and a "general" model. The general model is a single model obtained by fine tuning MACE-MP-0b3 on the joined training sets of the system specific models. The training set of the general model accounts for 4150 structures.

In Table S34, we report the training errors of the general model. Overall, the model achieves low training errors with a validation set RMSE of ~ 0.6 meV/atom on the energy and ~ 17.7 meV/Å on the forces.

Table S34: Training and Validation Errors for Energy (meV/atom), Forces (meV/Å), and Stress (meV/$\mathrm{Å}^3$) of the general model.

Dataset	RMSE Energy [meV/atom]	RMSE Forces [meV/Å]	RMSE Stress [meV/$\mathrm{Å}^3$]
Training	0.1	7.3	0.6
Validation	0.6	17.7	0.6

In Figs. S37 and S38, we report a comparison between the performance of the system specific models (blue), the general model (orange), and the starting foundation model (grey) on the lattice energy and EOS benchmark. The performance of the starting foundation model is reported to highlight the gain in accuracy due to the fine tuning procedure. We note, however, that the large differences reported are expected since the starting model is not trained to reproduce the vdW-DF2 functional. We find that the general model correctly reproduces the lattice energy with a RMSE of ~ 0.15 kJ/mol, compared to ~ 0.10 kJ/mol of the system specific models. The performance of the models on the EOS is again measured with the Δ (see Eq. 12) and ϵ (see Eq. 13) metrics. Overall, the general model also correctly reproduces the EOS, with a Δ RMSE of ~ 0.13 kJ/mol (as opposed to ~ 0.11 kJ/mol of the system specific models) and an average $1 - \epsilon^2 \sim 0.92$ (as opposed to ~ 0.94 of the system specific models).

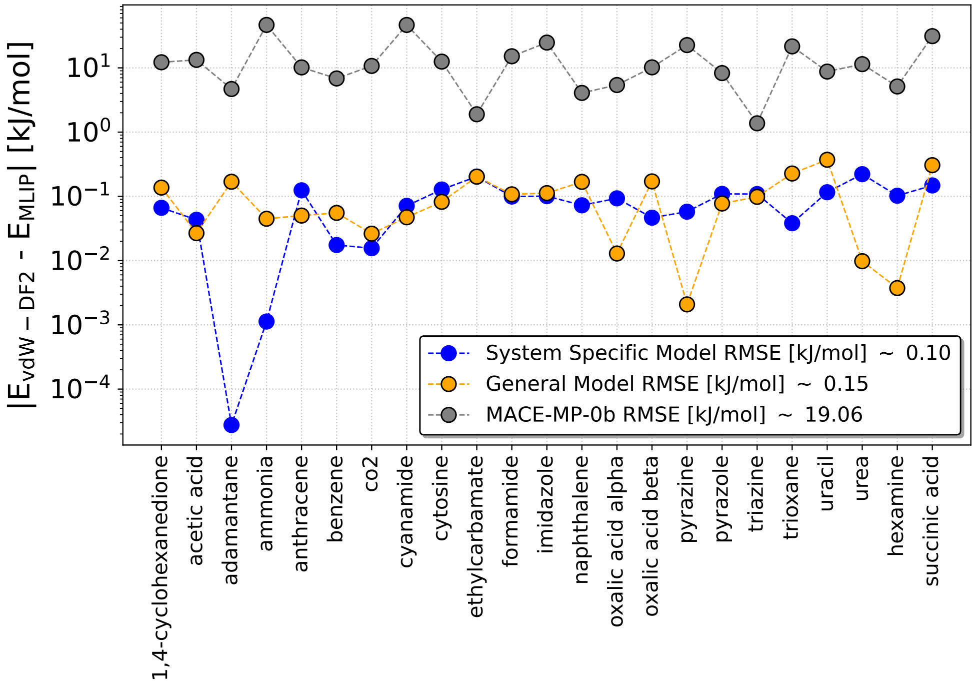

Figure S37: Comparison of the system specific and general models, and the starting foundation model, on the lattice energy benchmark. The plot shows the lattice energy error with respect to the reference DFT functional (vdW-DF2) for the system specific models (blue), the general model (orange), and the starting foundation model (grey).

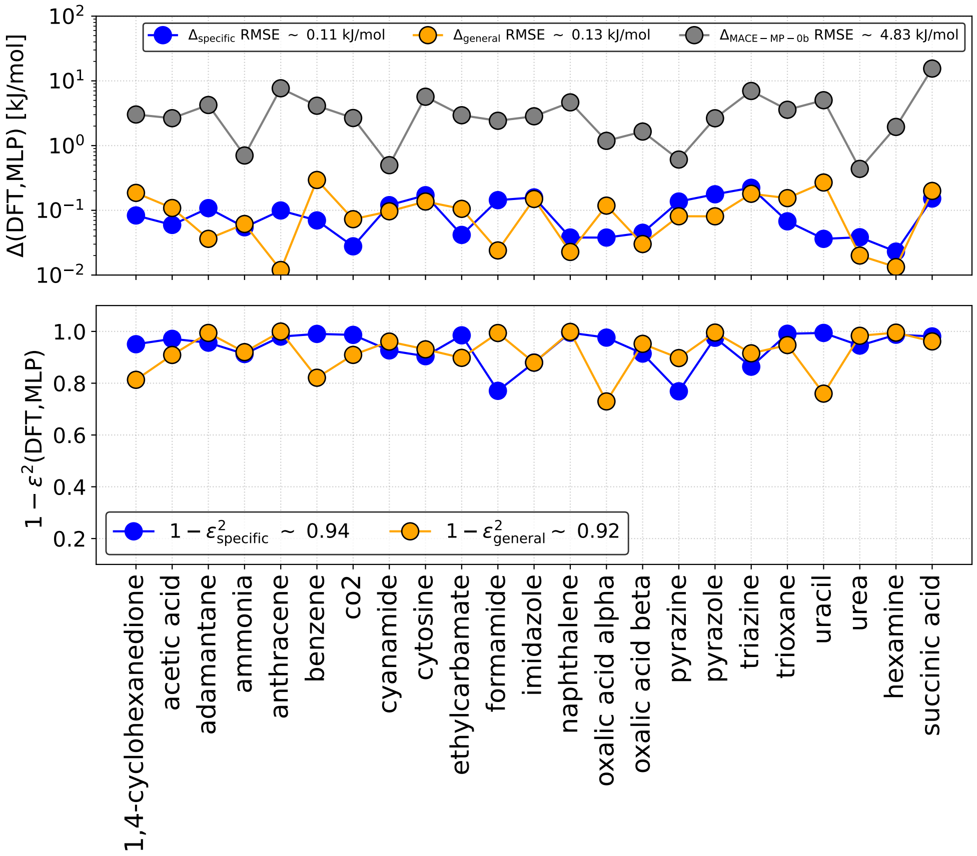

Figure S38: Comparison of the system specific and general models, and the starting foundation model, on the EOS benchmark. The plot shows the EOS metrics Δ (see Eq. 12) and ϵ (see Eq. 13) for the system specific models (blue), the general model (orange), and the starting foundation model (grey). The ϵ metric is not reported for the foundation model.

Finally, in Fig. S39 we report a comparison between the sublimation enthalpies computed with the MD approach by using the system specific models and the general model. Overall, we find that the system specific models and the general model achieve equivalent accuracy, with a difference in the prediction (measured as the MAE of the prediction of the general model against the system specific models) that is ~ 0.65 kJ/mol.

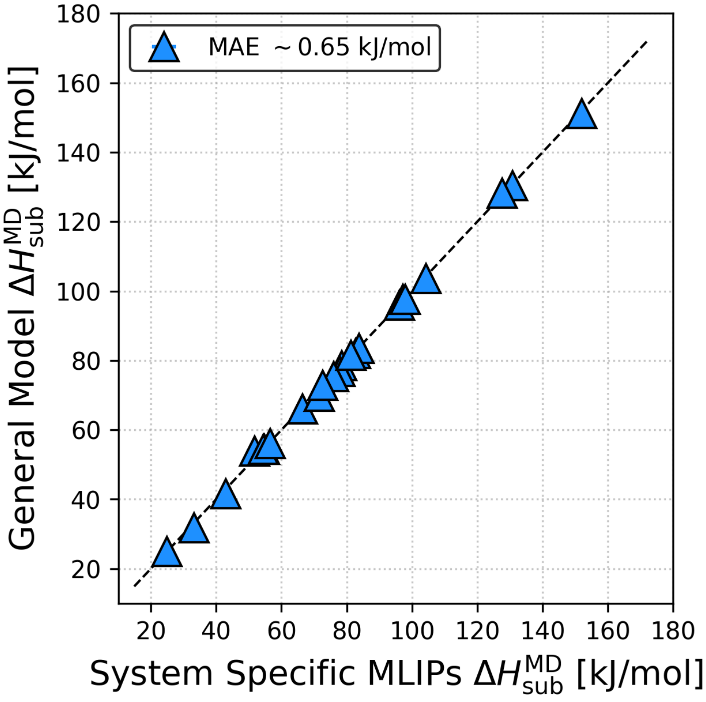

Figure S39: Comparison of the system specific and general models on the MD sublimation enthalpy. We report the scatter plot of the MD sublimation enthalpy (in kJ/mol) computed with the general model (y axis) against the system specific models (x axis).

S14 Anharmonicity in succinic acid

The system in the X23 dataset where anharmonicity plays the larger role is succinic acid. As shown in the main manuscript, the inclusion of anharmonicity and NQEs with the PIMD approach defines a $\sim$ 11 kJ/mol correction to the QHA sublimation enthalpy.

In Fig. S40, we show the torsion (or dihedral) angle of the four carbon atoms of the succinic acid molecule (C1-C2-C3-C4 in the inset) in a $\sim$ 1 ns long MD simulation. In particular, we plot the dihedral angle as a function of the time (left panel), as well as the probability distribution (right panel) estimated as a histrogram of the dihedral angle as a function of the time. Fig. S40 shows that the dihedral angle oscillates over time among $\sim 75°$, $\sim 180°$, and $\sim 290°$. The change over time of the torsion angle is an anharmonic feature, that cannot be described within the harmonic approximation where only small displacements of the atoms are allowed.

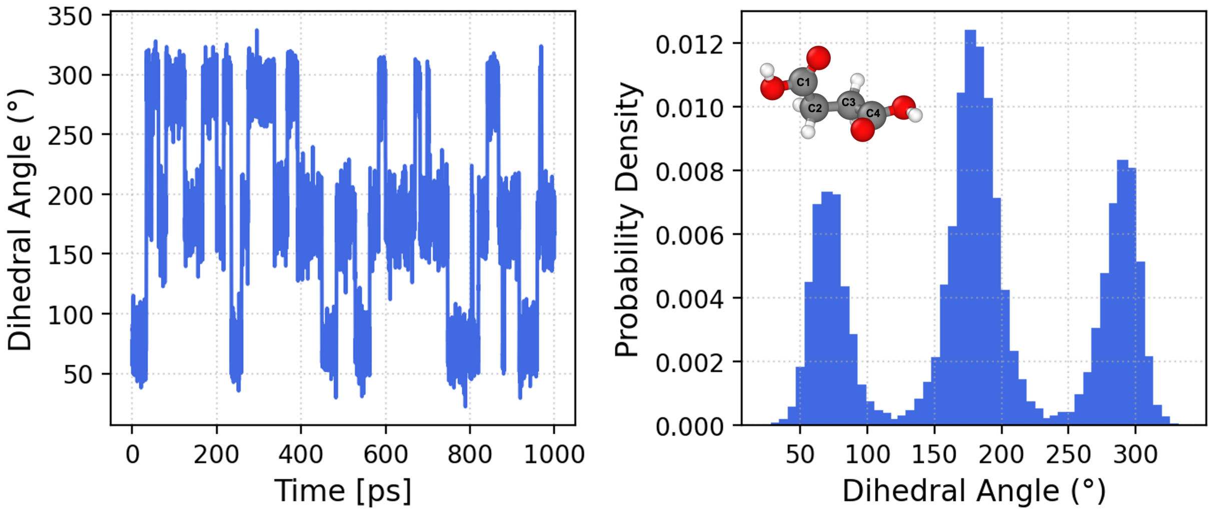

Figure S40: Dihedral angle in gas phase succinic acid. (left panel) Dihedral angle of the 4 carbon atoms in the gas phase succinic acid molecule as a function of time in the classical molecular dynamics simulation at room temperature. (right panel) Probability distribution of the dihedral angle from the simulation in the left panel. The succinic acid molecule is shown in the inset, with the carbon atoms defining the dihedral angle marked as C1, C2, C3, C4.

S15 Paracetamol, Aspirin and Squaric acid

The strategy proposed in this work allows for the data efficient fine tuning of models that achieve sub-chemical accuracy error with respect to the reference method for molecular crystals. As mentioned in the main manuscript, this opens up the road towards simulations with DFT accuracy of systems relevant of pharmaceuticals and biological interest.

In this section, we support this statement by showcasing the framework described in the main manuscript for molecular crystals of pharmaceutical interest, such as paracetamol, aspirin, and squaric acid. In particular, squaric acid was selected due to the importance of the inclusion of NQEs for its accurate description.[S9]

Differently from the fine tuning for the X23 molecular crystals, in this case we generated the initial EOS with the general X23 model described in Sec. S13. Only for squaric acid, the gas phase structures were generated using MACE-MP-0b3. The gas phase of squaric acid was in fact unstable with the general X23 model, probably because of the square ring of carbon atoms that is not contained in any structures of the X23 dataset. We subsequently follow the strategy described in the main manuscript, using the general X23 model for the first iteration of the data generation step (see Fig. ?? of the main manuscript).

In the following, we report the benchmark of the fine tuned models on the EOS and the QHA vibrational properties, as well as the computational details of the fine tuned model and the calculation of the sublimation enthalpies with QHA, MD, and PIMD.

S15.1 Equation of State

In Fig. S41 we report the EOS for form I of paracetamol (a), form I of aspirin (b), and squaric acid (c) with vdW-DF2 (black, DFT in the legend), the fine tuned model (blue, MLP in the legend) and the general X23 model described in Sec. S13 (green, X23 in the legend). The minimum of the energy is set to the lattice energy computed with the respective model (i.e. the difference between the energy per molecule of the solid and the energy of the gas phase).

The fine tuned models correctly reproduces the EOS with sub-kJ/mol errors. The energy errors are instead larger for the general X23 model ($\sim$ 10 kJ/mol), especially for squaric acid.

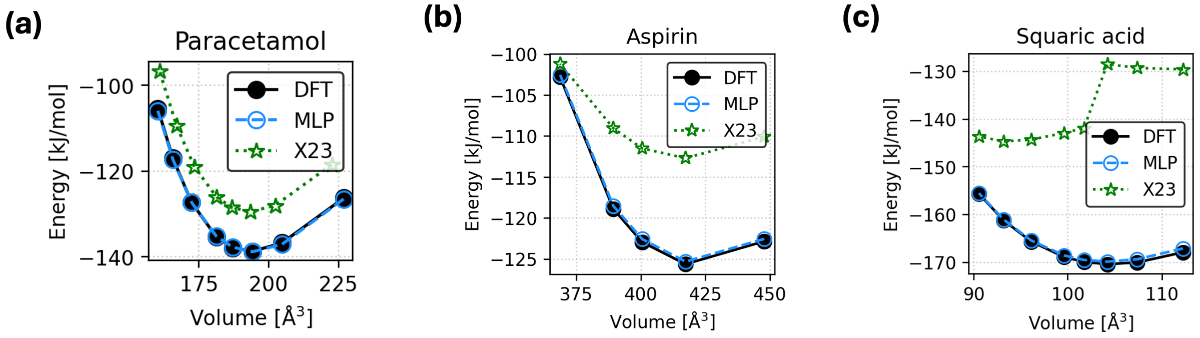

Figure S41: Equation of state of (a) form I of paracetamol, (b) form I of aspirin, and (c) squaric acid. In each plot we show the EOS computed with the reference DFT functional (black), the fine tuned MLIP (blue), and the general X23 model (green). The zero of the energy is set to the lattice energy E_{latt}.

S15.2 QHA vibrational properties

In Figs. S42, S43, and S44, we report respectively report the solid vibrational properties in the QHA for paracetamol, aspirin, and squaric acid. Each figure shows the vibrational density of states (top panel), the vibrational energy (bottom left panel) and the constant volume heat capacity (bottom right panel) with vdW-DF2 (black, DFT in the legend), the fine tuned model (blue, MLP in the legend) and the general X23 model (green, X23 in the legend). The vibrational properties are correctly reproduced with both the fine tuned model and the general X23 model, with errors < 1 kJ/mol on the vibrational energy.

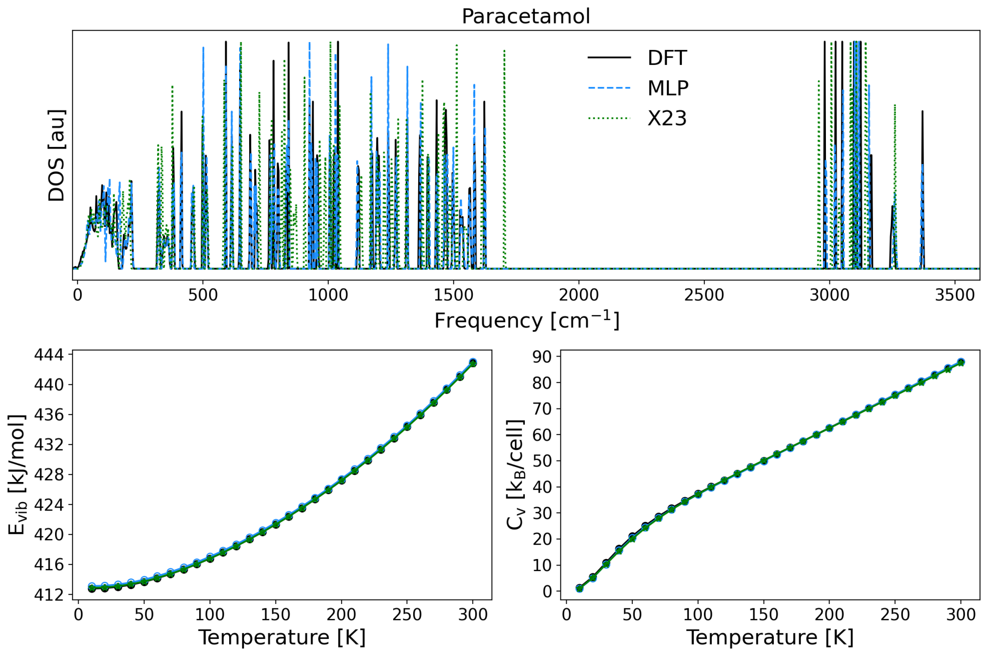

Figure S42: Benchmark of the fine tuned MLIPs on the quasi-harmonic vibrational properties of paracetamol. The plot reports the vibrational density of states (top panel), the vibrational energy (bottom left panel) and the constant volume heat capacity C_V (bottom right panel) computed with vdW-DF2 (black), the fine tuned MLIP (blue), and the general X23 model (green).

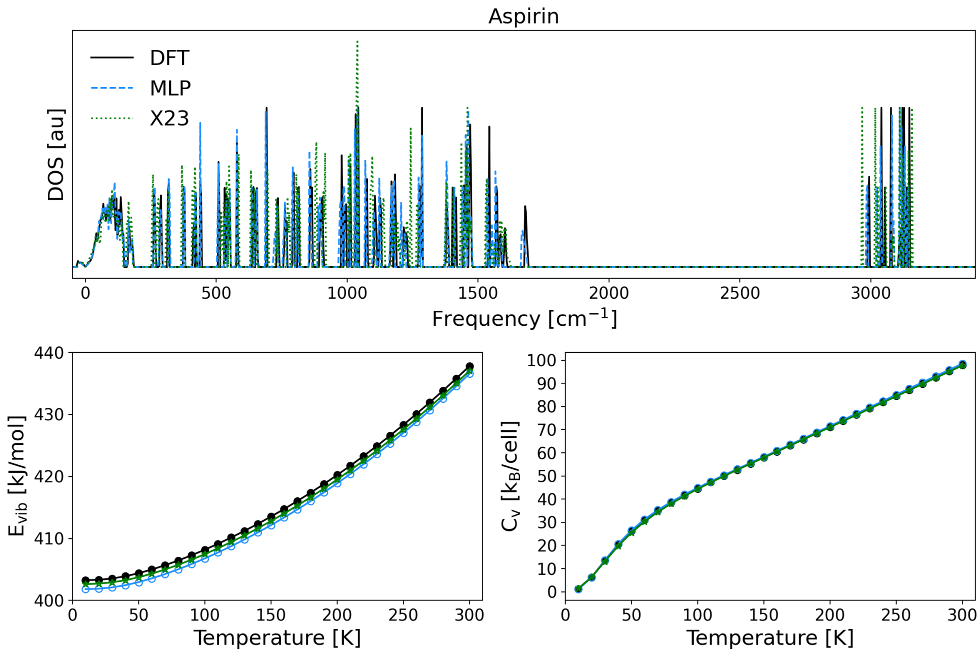

Figure S43: Benchmark of the fine tuned MLIPs on the quasi-harmonic vibrational properties of aspirin. The plot reports the vibrational density of states (top panel), the vibrational energy (bottom left panel) and the constant volume heat capacity C_V (bottom right panel) computed with vdW-DF2 (black), the fine tuned MLIP (blue), and the general X23 model (green).

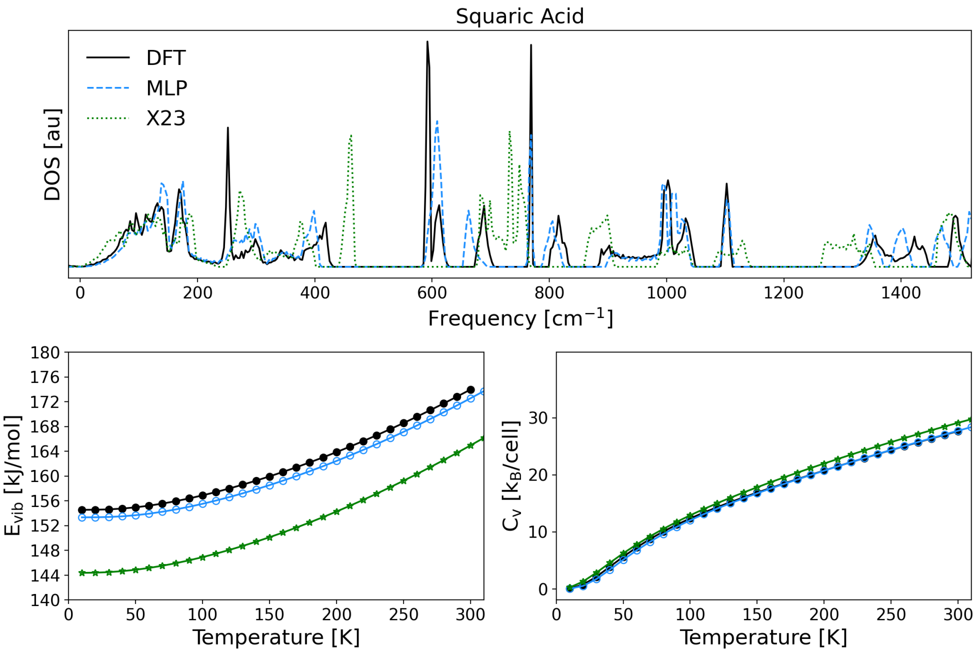

Figure S44: Benchmark of the fine tuned MLIPs on the quasi-harmonic vibrational properties of squaric acid. The plot reports the vibrational density of states (top panel), the vibrational energy (bottom left panel) and the constant volume heat capacity C_V (bottom right panel) computed with vdW-DF2 (black), the fine tuned MLIP (blue), and the general X23 model (green).

S15.3 Computational details

S15.3.1 Fine tuned models

The number of structures in the training set of the fine tuned models are reported in Tab. S35.

The fine tuning errors on the training and validation set for the three fine tuned models are reported in Tab. S36.

Table S35: Number of structures included in the training set of paracetamol, aspirin, and squaric acid.
[a] The number of structures is higher for paracetamol because structures of the polymorph II were also included in the training set.

System	Number of structures
Paracetamol[a]	364
Aspirin	150
Squaric Acid	199

Table S36: Training and Validation Errors for Energy (meV/atom), Forces (meV/Å), and Stress (meV/Å^3) of the fine tuned models for the paracetamol, aspirin, and squaric acid.

Dataset	RMSE Energy [meV/atom]	RMSE Forces [meV/Å]	RMSE Stress [meV/Å^3]
Paracetamol - Training	0.1	3.9	0.5
Paracetamol - Validation	0.2	10.3	0.5
Aspirin - Training	0.1	3.6	0.4
Aspirin - Validation	0.2	21.3	0.5
Squaric acid - Training	0.1	2.4	1.6
Squaric acid - Validation	0.2	17.1	1.8

S15.3.2 Density Functional Theory and QHA

The DFT calculations are performed with VASP[S10–S13] using the same set-up described in the main manuscript. The k-point grid used for the DFT calculations of the EOS are respectively $3 \times 3 \times 3$ for paracetamol, $2 \times 3 \times 2$ for aspirin, and $3 \times 3 \times 3$ for squaric acid. The DFT vibrational properties are computed with the small displacement method using PHON[S14] with a displacement of ~ 0.01 Å. The forces are computed with VASP at the Γ point, using respectively a $2 \times 2 \times 1$ supercell for paracetamol, a $1 \times 2 \times 1$ supercell for

aspirin, and a $3 \times 3 \times 3$ supercell for squaric acid. The vibrational energies are computed by integrated the frequencies over a $20 \times 20 \times 20$ grid.

S15.3.3 MD and PIMD sublimation enthalpies

The MD and PIMD simulations for the sublimation enthalpies are performed with i-PI[S15] using ASE[S16] as the force provider. We use the same barostat-thermostat setting described in the main manuscript for the X23 dataset. Input and output files are provided on GitHub.

Differently from the sublimation enthalpies of the X23 dataset, for paracetamol, aspirin, and squaric acid we do not apply the DMC correction for the lattice energy contribution. Therefore, the sublimation enthalpies are computed as

$$\Delta H_{\text{sub}}^{\text{QHA}} = E_{\text{gas}}^{\text{el,MLIP}} - E_{\text{sol}}^{\text{el,MLIP}} + E_{\text{gas}}^{\text{vib,MLIP}} - E_{\text{sol}}^{\text{vib,MLIP}} + 4RT, \tag{16}$$

with the QHA approach, as

$$\Delta H_{\text{sub}}^{\text{MD}} = \langle U \rangle_{\text{gas}} - \langle U \rangle_{\text{sol}} + \langle K \rangle_{\text{gas}} - \langle K \rangle_{\text{sol}} + \frac{5}{2}RT - p\langle V \rangle_{\text{sol}}, \tag{17}$$

with the MD approach, and

$$\Delta H_{\text{sub}}^{\text{PIMD}} = \langle K_{\text{cv}} + U \rangle_{\text{gas}} - \langle K_{\text{cv}} + U \rangle_{\text{sol}} + RT - p\langle V \rangle_{\text{sol}}, \tag{18}$$

with the PIMD approach.

References

(S1) Della Pia, F.; Zen, A.; Alfè, D.; Michaelides, A. How Accurate Are Simulations and Experiments for the Lattice Energies of Molecular Crystals? Phys. Rev. Lett. **2024**, 133, 046401.

(S2) Dolgonos, G. A.; Hoja, J.; Boese, A. D. Revised values for the X23 benchmark set of molecular crystals. Phys. Chem. Chem. Phys. **2019**, 21, 24333–24344.

(S3) Ceriotti, M.; Manolopoulos, D. E. Efficient First-Principles Calculation of the Quantum Kinetic Energy and Momentum Distribution of Nuclei. Phys. Rev. Lett. **2012**, 109, 100604.

(S4) Della Pia, F.; Zen, A.; Alfè, D.; Michaelides, A. DMC-ICE13: Ambient and high pressure polymorphs of ice from diffusion Monte Carlo and density functional theory. The Journal of Chemical Physics **2022**, 157, 134701.

(S5) Lejaeghere, K.; Bihlmayer, G.; Björkman, T.; Blaha, P.; Blügel, S.; Blum, V.; Caliste, D.; Castelli, I. E.; Clark, S. J.; Corso, A. D.; de Gironcoli, S.; Deutsch, T.; Dewhurst, J. K.; Marco, I. D.; Draxl, C.; Dułak, M.; Eriksson, O.; Flores-Livas, J. A.; Garrity, K. F.; Genovese, L.; Giannozzi, P.; Giantomassi, M.; Goedecker, S.; Gonze, X.; Grånäs, O.; Gross, E. K. U.; Gulans, A.; Gygi, F.; Hamann, D. R.; Hasnip, P. J.; Holzwarth, N. A. W.; Iuşan, D.; Jochym, D. B.; Jollet, F.; Jones, D.; Kresse, G.; Koepernik, K.; Küçükbenli, E.; Kvashnin, Y. O.; Locht, I. L. M.; Lubeck, S.; Marsman, M.; Marzari, N.; Nitzsche, U.; Nordström, L.; Ozaki, T.; Paulatto, L.; Pickard, C. J.; Poelmans, W.; Probert, M. I. J.; Refson, K.; Richter, M.; Rignanese, G.-M.; Saha, S.; Scheffler, M.; Schlipf, M.; Schwarz, K.; Sharma, S.; Tavazza, F.; Thunström, P.; Tkatchenko, A.; Torrent, M.; Vanderbilt, D.; van Setten, M. J.; Speybroeck, V. V.; Wills, J. M.; Yates, J. R.; Zhang, G.-X.; Cottenier, S. Reproducibility in density functional theory calculations of solids. Science **2016**, 351, aad3000.

(S6) Bosoni, E.; Beal, L.; Bercx, M.; Blaha, P.; Blügel, S.; Bröder, J.; Callsen, M.; Cottenier, S.; Degomme, A.; Dikan, V.; Eimre, K.; Flage-Larsen, E.; Fornari, M.; Garcia, A.; Genovese, L.; Giantomassi, M.; Huber, S. P.; Janssen, H.; Kastlunger, G.; Krack, M.; Kresse, G.; Kühne, T. D.; Lejaeghere, K.; Madsen, G. K. H.; Marsman, M.; Marzari, N.; Michalicek, G.; Mirhosseini, H.; Müller, T. M. A.; Petretto, G.; Pickard, C. J.; Poncé, S.; Rignanese, G.-M.; Rubel, O.; Ruh, T.; Sluydts, M.; Vanpoucke, D. E. P.; Vijay, S.; Wolloch, M.; Wortmann, D.; Yakutovich, A. V.; Yu, J.; Zadoks, A.; Zhu, B.; Pizzi, G. How to verify the precision of density-functional-theory implementations via reproducible and universal workflows. Nature Reviews Physics **2024**, 6, 45–58.

(S7) Otero-de-la Roza, A.; Johnson, E. R. A benchmark for non-covalent interactions in solids. The Journal of Chemical Physics **2012**, 137, 054103.

(S8) Reilly, A. M.; Tkatchenko, A. Understanding the role of vibrations, exact exchange, and many-body van der Waals interactions in the cohesive properties of molecular crystals. The Journal of Chemical Physics **2013**, 139, 024705.

(S9) Li, X.-Z.; Walker, B.; Michaelides, A. Quantum nature of the hydrogen bond. Proceedings of the National Academy of Sciences **2011**, 108, 6369–6373.

(S10) Kresse, G.; Hafner, J. Ab initio molecular dynamics for liquid metals. Phys. Rev. B **1993**, 47, 558.

(S11) Kresse, G.; Hafner, J. Ab initio molecular-dynamics simulation of the liquid-metal-amorphous-semiconductor transition in germanium. Phys. Rev. B **1994**, 49, 14251.

(S12) Kresse, G.; Furthmüller, J. Efficiency of ab-initio total energy calculations for metals and semiconductors using a plane-wave basis set. Computational Materials Science **1996**, 6, 15–50.

(S13) Kresse, G.; Furthmüller, J. Efficient iterative schemes for ab initio total-energy calculations using a plane-wave basis set. Phys. Rev. B. **1996**, 54, 11169.

(S14) Alfè, D. PHON: A program to calculate phonons using the small displacement method. Computer Physics Communications **2009**, 180, 2622–2633, 40 YEARS OF CPC: A celebratory issue focused on quality software for high performance, grid and novel computing architectures.

(S15) Litman, Y.; Kapil, V.; Feldman, Y. M. Y.; Tisi, D.; Begušić, T.; Fidanyan, K.; Fraux, G.; Higer, J.; Kellner, M.; Li, T. E.; Pós, E. S.; Stocco, E.; Trenins, G.; Hirshberg, B.; Rossi, M.; Ceriotti, M. i-PI 3.0: A flexible and efficient framework for advanced atomistic simulations. The Journal of Chemical Physics **2024**, 161, 062504.

(S16) Larsen, A. H.; Mortensen, J. J.; Blomqvist, J.; Castelli, I. E.; Christensen, R.; Dułak, M.; Friis, J.; Groves, M. N.; Hammer, B.; Hargus, C.; Hermes, E. D.; Jennings, P. C.; Jensen, P. B.; Kermode, J.; Kitchin, J. R.; Kolsbjerg, E. L.; Kubal, J.; Kaasbjerg, K.; Lysgaard, S.; Maronsson, J. B.; Maxson, T.; Olsen, T.; Pastewka, L.; Peterson, A.; Rostgaard, C.; Schiøtz, J.; Schütt, O.; Strange, M.; Thygesen, K. S.; Vegge, T.; Vilhelmsen, L.; Walter, M.; Zeng, Z.; Jacobsen, K. W. The atomic simulation environment—a Python library for working with atoms. Journal of Physics: Condensed Matter **2017**, 29, 273002.
